# Quinone derivatives isolated from the endolichenic fungus *Phialocephala fortinii* are Mdr1 modulators that combat azole resistance in *Candida albicans*

**DOI:** 10.1038/srep33687

**Published:** 2016-09-21

**Authors:** Fei Xie, Wenqiang Chang, Ming Zhang, Ying Li, Wei Li, Hongzhuo Shi, Sha Zheng, Hongxiang Lou

**Affiliations:** 1 Department of Natural Product Chemistry, Key Lab of Chemical Biology of the Ministry of Education, Shandong University, No. 44 West Wenhua Road, Jinan City, Shandong Province, China

## Abstract

One of the main azole-resistance mechanisms in *Candida* pathogens is the upregulation of drug efflux pumps, which compromises the efficacy of azoles and results in treatment failure. The combination of azole-antifungal agents with efflux pump inhibitors represents a promising strategy to combat fungal infection. High-throughput screening of 150 extracts obtained from endolichenic fungal cultures led to the discovery that the extract of *Phialocephala fortinii* exhibits potent activity for the reversal of azole resistance. From *P. fortinii* cultures, a total of 15 quinone derivatives, comprising 11 new derivatives and 4 known compounds, were obtained. Among these compounds, palmarumycin P3 (**3**) and phialocephalarin B (**8**) specifically modulate the expression of *MDR1* to inhibit the activity of drug efflux pumps and therefore reverse azole resistance. The present study revealed Mdr1 targeting as an alternative mechanism for the discovery of new agents to fight antifungal drug resistance.

*Candida albicans* is the most frequent human commensal opportunistic fungal pathogen, resulting in high morbidity and mortality, particularly in immunosuppressed patients^1^,^2^,^3^. Reflecting the widespread and prolonged usage of antibiotics, the emergence of pathogenic fungi with multidrug resistance (MDR) is also increasing, further complicating antifungal therapy^4^,^5^. Azole antifungal drugs are commonly used for fungal infections, but an increasing incidence of azole resistance is occurring in the clinic^6^. The mechanisms leading to azole resistance include alterations in the sterol biosynthetic pathway, increased expression of the *ERG11* gene encoding the target enzyme of fluconazole (FLC), sterol 14α-demethylase (Erg11), mutations in the *ERG11* gene resulting in the reduced affinity of Erg11 to FLC, and the overexpression of genes encoding membrane transport proteins, which pump FLC out of the cell^7^,^8^,^9^. Among these pumps, the overexpression of drug transporters is a principal mechanism utilized by *Candida* species to alleviate antibiotic stress through a reduction in the intracellular accumulation. In *Candida* species, 2 gene transporter families, the *CDR* genes of the ATP-binding cassette super family and the *MDR* genes of the major facilitators class, encode drug transporters^10^. In *Candida albicans*, Cdr1 and Cdr2 are ATP-binding cassette transporters that use energy derived from ATP hydrolysis to transport drugs outside the cells, while Mdr1, a major facilitator superfamily (MFS) protein, utilizes a proton gradient for drug extrusion^9^. Many FLC-resistant clinical *C. albicans* isolates constitutively overexpress *MDR1*^11^,^12^,^13^,^14^,^15^. The inactivation of *MDR1* in *MDR1*-overexpressing *C. albicans* isolates is an important pathway to increase the susceptibility of these microbes to FLC^16^.

The combination of azoles and other non-antifungal agents, such as specific inhibitors of efflux pumps, is a promising approach to manage resistant *Candida* infections^17^,^18^,^19^,^20^,^21^. Natural products are an important source for the discovery of active agents, reflecting the versatile structures of the products^22^,^23^,^24^. In previous studies, we focused on the development of diversified natural products with antifungal activities from bryophytes or endolichenic fungi^25^,^26^,^27^,^28^. Therefore, we developed both an Alamar Blue assay and an agar diffusion assay to screen a natural products library for hits that chemosensitize *C. albicans* to fluconazole (FLC) treatment.

In the present study, we examined the reversal of azole resistance in 150 extracts from endolichenic fungi cultures, leading to the discovery that the extract of *Phialocephala fortinii* displayed potent activity to reverse azole resistance. Isolation of *P. fortinii* metabolites afforded 15 quinone derivatives. Among the isolated compounds, palmarumycin P3 (**3**) and phialocephalarin B (**8**), which two representatives of the compounds obtained, could specifically modulate the expression of *MDR1* to inhibit the activity of drug efflux pumps and therefore reverse azole resistance.

## Results

### HTSS for antifungal hits from a microbial natural product library

A library of 150 endolichenic fungi was isolated from collected lichens. We prepared microbial fermentation extracts of the endolichenic fungi and screened them to identify the hits using Alamar Blue or agar diffusion assays. These hits should show low antifungal activity by themselves and potent enhancement of the efficacy of FLC against azole-resistant *C. albicans* isolates. Among 150 culture extracts, *P. fortinii* culture demonstrated potent capability of reversal of azole resistance and low cell toxicity (Supplementary Results Fig. S1). Thus, *P. fortinii* was fermented at a large scale for subsequent analysis.

### Identification of single compounds as active components in the hit

**Figure 1:**
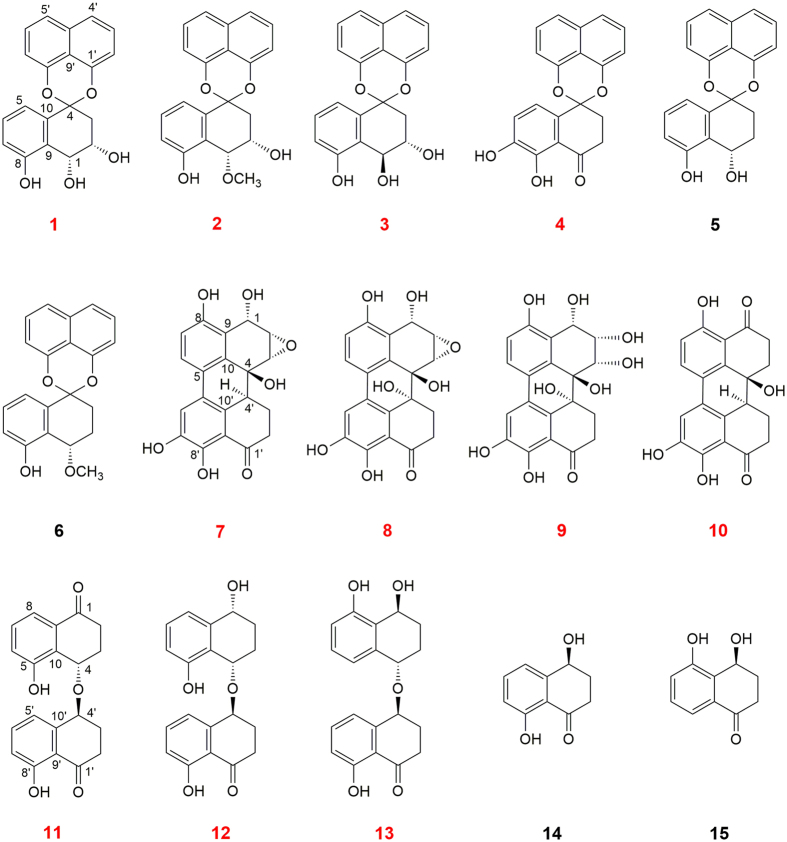
Structures of the compounds isolated from the endolichenic fungal *Phialocephala fortinii*. The new compounds are indicated with red colour.

*P. fortinii* culture was extracted using EtOAc and repeatedly subjected to chromatography over silica gel, Sephadex LH-20, MPLC and further semi-preparative HPLC under bioassay-guided separation, generated fifteen quinone derivatives, including six spirobisnaphthalenes (**1–6**), four perylenequinones (**7–10**) and five naphthalenone (**11–15**). Among these derivatives, eleven compounds were novel compounds, indicated in a red colour (Fig. 1). To elucidate the structures of new compounds, including palmarumycin P1-P4 (**1**–**4**), phialocephalarin A-D (**7–10**), and juglanone C-E (**11**–**13**), HRESIMS, ^1^H and ^13^C NMR, and 2D NMR were performed. The spectra revealed that these compounds have similar structural features as members of the spirobisnaphthalenes, perylenequinones, and naphthalenones, respectively (Supplementary Results Tables S1–S3). By determining HMBC spectra, the planar structures of these compounds were unambiguously established. The absolute configurations of **2**, **3**, **4**, and **7** were further determined based on a single-crystal X-ray diffraction analysis with Cu Kα radiation (Supplementary Results). The absolute configuration assignments of the other new compounds were determined through a comparison of the CD spectra (Supplementary Results Figs S8, 47, 55, 63, 71, 79, and 87). The known compounds were identified through a comparison of the spectroscopic data with previously reported data^29^,^30^,^31^.

**Figure 2:**
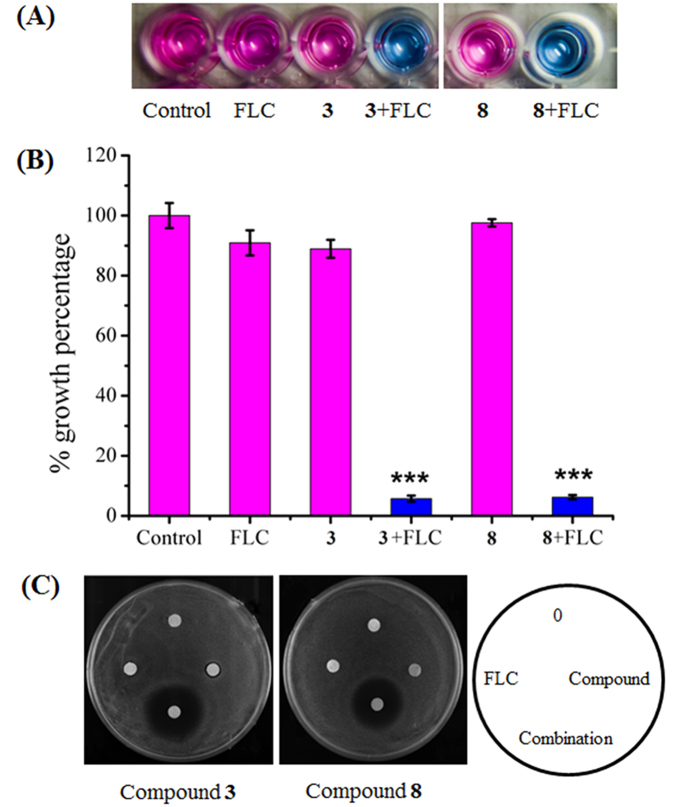
Synergistic effects between compounds and FLC against azole-resistant *C. albicans* 24D. (**A**,**B**) Growth inhibitory effects against *C. albicans* 24D under the indicated treatments were revealed using the Alamar blue assay. The cells were treated with the indicated drugs for 48 h, followed by further analysis using Alamar blue staining for 2 h in the dark, revealing pink supernatant when the cells proliferated (**A**). The growth percentage was measured using a spectrophotometer at 570 nm (**B**). (**C**) The growth inhibitory effect under the indicated treatments was revealed using the disk diffusion assay. For this assay, the individual test organism 24D (1 × 10^6^ CFUs/ml) was plated on Mueller Hinton agar (MHA) medium supplemented with 2% glucose. Cellulose disks impregnated with FLC (2 μg), either compound (64 μg) or a combination of each agent (2 μg of FLC and 16 μg of compound) were placed onto MHA agar plates. Each plate was incubated at 30 °C for 48 h for the agar diffusion assay.

All the pure compounds were assayed for the inhibition of *C. albicans* growth or the reversal of the azole resistance of clinical strain 24D, which displays relatively high transcriptional expression of *MDR1* when incubated with FLC among our collected clinical isolates (unpublished data). The results showed that these compounds alone did not exert any inhibitory activity against the growth of *C. albicans*. However, spirobisnaphthalene and perylenequinone derivatives (**1**–**10**) conferred 64-fold or higher sensitivity on strain 24D to FLC (Fig. 2 and Supplementary Results Table S4). Because compounds **3** and **8** are the major constituents in the EtOAc extract, these compounds were selected for the subsequent studies.

### Inhibition of *C. albicans* multidrug resistance through the modulation of efflux pumps

**Figure 3:**
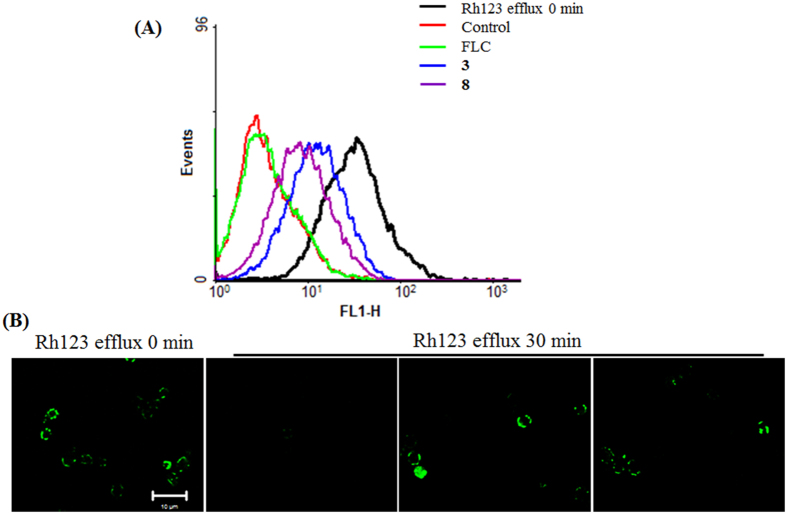
The effects of FLC or the combined compounds 3 and 8 on the activity of efflux pumps. (**A**) The measurement of Rh123 efflux, induced through glucose in the FLC-resistant strain 24D, was assessed using flow cytometry detection. (**B**) The intracellular accumulation of Rh123 in *C. albicans* cells in response to treatment with compound **3** (16 μg/ml) or **8** (16 μg/ml) was revealed through CLSM observation.

Compounds **3** and **8** displayed azole-reversal effects together with FLC against several clinical azole-resistant strains, including 24D, 28I, CA10, CA406, CA417, and CA631 (Table 1). The Alamar Blue assay showed that the addition of **3** or **8** could facilitate inhibition of the growth of *C. albicans* strain 24D by 94.25 ± 1.04% and 93.77 ± 0.79%, respectively, through FLC, whereas FLC alone caused only a 9.11 ± 4.2% reduction in growth (Fig. 2A,B). The agar plate assay further confirmed the observed enhanced inhibitory action when FLC was applied together with compound **3** or **8** (Fig. 2C). The cytotoxicities of these two compounds were evaluated based on the IC_50_ values against the following normal cell lines: human umbilical vein endothelial cells (HUVEC), human bronchial epithelium (HBE) cells and non-neoplastic, immortalized human prostatic epithelial (RWPE-1) cells. The IC_50_ values of compounds **3** and **8** were much higher than the dose used in the combination treatment (Table 2), suggesting low toxicity in the application. Upregulation of drug efflux pumps has been reported as one of the most important factors, conferring azole resistance^10^,^11^. The efflux activity assay revealed that compounds **3** and **8** could facilitate the accumulation of Rh123 based on the flow cytometry analysis and CLSM observation, suggesting an inhibitory effect on the efflux pumps (Fig. 3).

### Compounds 3 and 8 specifically modulate *MDR1* expression in *C. albicans*

**Figure 4:**
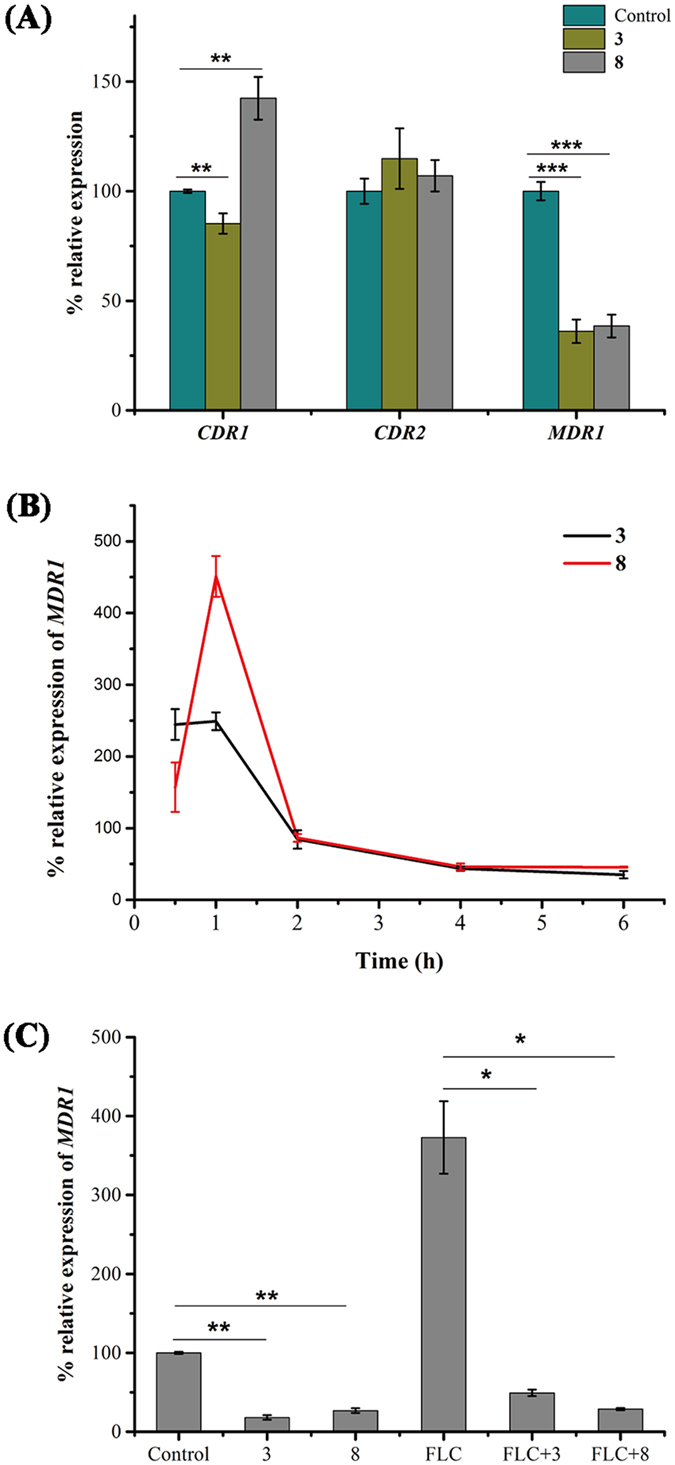
The transcript levels of transporter genes in response to treatment with compound 3 or 8 in *Candida*. (**A,B**) The azole-resistant isolate (24D) was incubated with FLC for 3 h at 30 °C and subsequently cultured in RPMI 1640 medium containing the indicated agents. (**A**) After 3 h of incubation, the relative expression of *CDR1, CDR2* and *MDR1* genes was determined through qPCR and normalized to 18S. (**B**) At indicated time ranging from 0.5 to 6 h, the *MDR1* expression of *C. albicans* in the presence of compound **3** or **8** was monitored by using qPCR. (**C**) *C. tropicalis* FLC-resistant isolate (NPC-T001) was treated with FLC (1 μg/mL), tested compound (8 μg/mL) or their combination. After 12 h of culture, RNA was extracted for *MDR1* transcript analysis by using qPCR. The bars represent the means ± SD. *P < 0.05, **P < 0.01, ***P < 0.001.

Most clinically drug-resistant isolates of *C. albicans* overexpress genes encoding Cdr1, Cdr2 or Mdr1 drug efflux pump proteins^32^,^33^,^34^. Here, we observed that compound **3** or **8** reduced the expression of *MDR1* in azole-resistant strain 24D (Fig. 4A). However, these two compounds had less potent effects on the reduction of *CDR1* or *CDR2* expression (Fig. 4A). The transcriptional expression of *MDR1* with time of exposure to compound **3** or **8** was monitored by using quantitative real-time PCR (qPCR). The results demonstrated that *MDR1* expression was firstly induced by **3** or **8** during the initial 1 hour, and followed by a decrease within the next 5 hours in our test (Fig. 4B). The induced expression at the initial 1 hour probably acts as a feedback of compromised efflux pump function.

To confirm that *MDR1* was the primary effector of compound **3** or **8**, mutant strains deficient in efflux pumps, including DSY488 (*cdrlΔ/Δ*), DSY653 (*cdr2Δ/Δ*), DSY465 (*mdrlΔ/Δ*), and DSY659 (*cdrlΔ/Δ, cdr2Δ/Δ*), were examined for susceptibility to the combination treatment of FLC and compound **3** or **8**. The results showed that the combination treatment displayed synergistic action against mutant strains lacking *CDR1*, *CDR2*, or both genes, while the synergistic index was lower in mutant strain deficient in *MDR1* (Tables 3 and 4). We also measured the effect of the combination treatment on a *CDR1* and *CDR2*-overexpressing strain (YEM15) and an *MDR1*-overexpressing strain (YEM13). We observed that compound **3** or **8** could reverse the drug resistance of YEM13 with a decrease in the minimal inhibitory concentration (MIC) of FLC from 64 to 2–4 μg/ml, whereas a high dose of compound **3** or **8** was required to sensitize YEM15 to FLC (Tables 3 and 4). These results implied that the regulation of *MDR1* was probably the prime target of compounds **3** and **8**.

### Compounds 3 and 8 reverse the FLC resistance of *C. tropicalis* through a reduction in *MDR1* expression

Compounds **3** and **8** could not only reverse the azole resistance of *C. albicans* strains but also sensitize the azole resistance of a *C. tropicalis* strain to FLC. We observed that compound **3** or **8** reduced the MIC of FLC against *C. tropicalis* strain NPC-T001 from 128 to 1 μg/ml when used at the concentration of 8 μg/ml. The qPCR assay revealed that compound **3** or **8** caused 5.54- or 3.73-fold reductions in *MDR1* expression, respectively, compared with the control. When the strain was exposed to FLC, the expression of *MDR1* increased 3.73-fold. However, the addition of compound **3** or **8** reduced the *MDR1* expression in FLC-treated cells by 7.56 or 12.98-fold, respectively (Fig. 4C). These results implied that compounds **3** and **8** could be developed as *MDR1* modulators to reverse the azole resistance in *Candida* species.

## Discussion

MDR in *Candida*, resulting from the overexpression of efflux pumps, is a major obstacle in antifungal chemotherapy. Identifying selective, low-toxicity inhibitors/modulators of MDR might be a promising strategy to combat this problem. Compounds from natural products represent one of the most diverse and novel chemical scaffolds suitable for the development of new inhibitors/modulators^35^,^36^,^37^,^38^. Many researchers have recognized the value of screening for new modulators from natural sources, as natural extracts are typically low in toxicity and well tolerated in the human body. In the present study, HTSS was applied to identify the chemosensitizers from 150 endolichenic fungal extracts. Six spirobisnaphthalene derivatives and four perylenequinone derivatives with eight novel structures were isolated from the fungal extracts and demonstrated to harbour the ability to reverse azole resistance. Compounds **3** and **8**, as the two major constituents of the spirobisnaphthalene and perylenequinone derivatives, were selected to investigate the mode of action. Compounds **3** and **8** inhibited the activity of efflux pumps, and could elevate the intracellular content of FLC when applied for treatment against azole-resistant strains. We subsequently utilized efflux pump-deficient strains and quantitative real-time PCR to verify that compounds **3** and **8** primarily affect the transcriptional levels of *MDR1* and have a less potent effect on the expression of *CDR1* and *CDR2*.

Overexpression of the multidrug efflux pump Mdr1 increased fluconazole resistance in *C. albicans*^10^. The upregulation of *MDR1* is controlled through the transcription factors Mrr1 and Cap1^39^. Gain-of-function mutations in Mrr1 or Cap1 render the transcription factors hyperactive and result in constitutive *MDR1* overexpression^39^,^40^. Mrr1 contains multiple activation and inhibitory domains, which regulate *MDR1* expression^41^. A previous study showed that the transcription factor Mcm1 was required for hyperactive Mrr1, causing *MDR1* overexpression^42^. To date, the direct mutual interaction between *MDR1* and transcription factors has not been elucidated. It is highly likely that compound **3** or **8** interferes with the interaction between the transcription of *MDR1* and the transcription factors of Mrr1 and Cap1, although further evidence is needed.

Several efforts have focused on discovering selective inhibitors or modulators to overcome MDR in cancer chemotherapy over the years^43^,^44^,^45^,^46^. However, little effort has been made to investigate antifungal actions, particularly in clinical application. The results of the present study indicate that quinone derivatives are *MDR1* modulators that can reverse azole resistance in *Candida* species. Compounds **3** and **8**, originating from natural products, represent more selective and potent chemosensitizers to improve FLC in treating fungal infections.

## Methods

### Strains and growth conditions

The fungus *Phialocephala fortinii* used in the present study was isolated from the lichen *Pamelia* sp., collected in Mount Qingliang, Zhejiang Province, China. The fungus was identified using nuclear 18S rDNA sequences (GenBank: AB208110), assigned the accession no. 4537d and deposited in the lichen laboratory in the College of Life Sciences, Shandong Normal University, Jinan. The *C. albicans* isolates used in the present study are shown in Supplementary Results Table S5. *C. albicans* was propagated in yeast-peptone dextrose (YPD) medium in an orbital shaker at 30 °C and assayed in RPMI 1640 medium. The normal cell lines HUVEC, HBE and RWPE-1 were cultured as previously described^27^,^47^,^48^,^49^.

### Crude extract preparation

A total of 150 endolichenic fungal species were provided from Professor Zuntian Zhao in Shandong Normal University. The individual colonies of each strain were streaked onto potato dextrose agar (PDA) plates. After 10 days of culture at 28 °C, the organisms were scraped and extracted using EtOAc for HTCC.

### HTSS for antifungal hits using Alamar Blue and agar diffusion assays

The drug susceptibility test for screening antifungal hits was performed using Alamar Blue and disk diffusion assays as previously described, with slight modifications^50^,^51^. For the Alamar Blue assay, *C. albicans* isolates were cultured in YPD medium (1% yeast extract, 2% bacto peptone and 2% dextrose) at 30 °C with rotational shaking at 200 rpm. Overnight cultured cells were collected, washed and diluted to a cell density of 1 × 10^3^ CFUs/ml in RPMI 1640 medium. Aliquots of 100 μl of the fungal suspension with 16, 32, and 64 μg/ml of the solubilized drugs containing 4 μg/ml FLC were added to the wells of 96-well flat-bottomed microtitration plates. After incubation for 48 h, 10 μl of Alamar blue was added to the wells, the subsequent colour change was photographed after 2 h of incubation in the dark, and the absorbance at 570 nm was measured using a spectrophotometer.

For the agar diffusion assay, overnight cultures were diluted using PBS to 1 × 10^7^ CFUs/ml. Aliquots of 100 μl of yeast suspension were spread onto Mueller Hinton agar (MHA) medium supplemented with 2% glucose. To examine the antifungal activity of each combination of partner drugs and FLC, cellulose disks impregnated with 16, 32, and 64 μg of the solubilized drugs and 4 μg of FLC and the control disk impregnated with the corresponding solvent were placed onto YPD agar plates. After 48 h of incubation at 30 °C, the horizontal and vertical diameters of the growth inhibition areas were recorded.

### Isolation of active compounds

The endolichenic fungus *P. fortinii* was cultivated in three 500-ml Erlenmeyer flasks, each of which contained 100 ml of potato dextrose broth (PDB), at 25 °C on a rotary shaker (120 rpm) for 7 days to prepare the seed culture. Large-scale fermentation was performed in twenty 500-ml Erlenmeyer flasks, each of which contained 80 g of autoclaved rice, and subsequently these cultures were inoculated with the spore inoculum (15 ml) and cultured for 50 days at room temperature. The culture was subsequently extracted three times using EtOAc (6 L), and the organic solvent was evaporated under reduced pressure to generate a crude extract (72.2 g). The EtOAc extract was repeatedly subjected to chromatography over silica gel, Sephadex LH-20, MPLC and further semi-preparative HPLC to afford fifteen compounds. Silica gel (200–300 mesh; Qingdao Haiyang Chemical Co. Ltd., Qingdao, P. R. China) and Sephadex LH-20 gel (25–100 mm; Pharmacia Biotech, Denmark) were used for column chromatography (CC). MPLC was performed on a Leisure EZ Purifier apparatus equipped with a UV-VIS dual wavelength detector (210 and 254 nm) (Leisure Science Corporation) and an ODS column (30 × 130 mm). HPLC was performed on an Agilent 1100 G1310A isopump equipped with a G1322A degasser, a G1314A VWD detector (210 nm), and a ZORBAX SB-C_18_ 5 mm column (9.4 × 250 mm).

### Elucidation of the chemical structures

Optical rotations were obtained using an Anton paar MCP 200 polarimeter. The UV data were recorded on a UV-2450 spectrophotometer (Shimadzu, Japan). The CD spectra were obtained on a Chirascan spectropolarimeter. The IR spectra were measured on a Nicolet iN 10 Micro FTIR spectrometer. The NMR spectra were recorded on a Bruker Avance DRX-600 spectrometer at 600 (^1^H) and 150 (^13^C) MHz, with TMS as an internal standard. HRESIMS was performed on a Finnigan LC-Q^DECA^ mass spectrometer, and x-ray crystallographic analyses were conducted on a Bruker D8 venture or Bruker APEX DUO diffractometer, employing APEX II CCD using Cu Kα radiation.

### Minimum inhibitory concentration determination

The minimum inhibitory concentrations (MIC_80_) of compounds against *Candida* species were determined through broth microdilution according to CLSI M27-A3 guidelines^52^. A susceptibility test of the efflux pump mutant strains was also conducted using the procedures for MIC determination.

### Cytotoxicity detection through the MTT assay

A 3-(4,5-dimethylthiazol-2-yl)-2,5-diphenyl-2H-tetrazoliumbromide (MTT, Sigma) colorimetric assay was used to assess the proliferation and cytotoxicity against HUVEC, HBE and RWPE-1 cells in the presence of compound **3** or **8**^47^,^48^,^49^,^53^. The cells (1 × 10^4^ per well) were seeded onto 96-well plates and incubated at 37 °C in a 5% CO_2_ incubator. After incubation of 24 h, the cells were treated with vehicle or desired concentrations of compounds **3** and **8** for an additional 24 h. After treatment for 24 h, the cells were incubated with MTT for an additional 4 h in the dark. The cell growth response to the chemicals was detected after measuring the light absorbance at 570 nm using a plate reader (Bio-Rad Laboratories, Richmond, CA). The IC_50_ values were calculated based on the percentage of viable cells.

### The interaction of the tested compounds with FLC against *Candida* species

To assess the nature of the *in vitro* interactions between the tested agents and FLC against *C. albicans* and *C. tropicalis* strains, an FICI model was used to characterize the interactions between the tested agents and FLC through analysis of the data obtained from broth microdilution checkerboard assays. The FICI model is described as Σ FIC = FIC_A_ + FIC_B_ = MIC_AB_/MIC_A_ + MIC_BA_/MIC_B_, where MIC_A_ and MIC_B_ are the MICs of drugs A and B when used alone, and MIC_AB_ and MIC_BA_ are the concentrations of drugs A and B in the iso-effective combinations, respectively. According to the results calculated from each dataset, synergy corresponds to a FICI value of ≤0.5, while antagonism reflects a FICI value of >4; otherwise, indifference is concluded^54^.

### Transport Assays

Transport assays were conducted by monitoring rhodamine 123 (Rh123) accumulation. The accumulation of Rh123 in azole-resistant cells was measured using flow cytometry (Becton-Dickinson Immunocytometry Systems) and observed using confocal microscopy. Briefly, overnight-cultured cells were collected, washed and resuspended in YPD medium. After incubation at 30 °C for 4 h with shaking, the cells were pelleted, washed and incubated with 5 μM Rh123 at 30 °C for 30 min in PBS, and subsequently the cells were incubated for an additional 30 min in the absence or presence of the tested compounds. The cells were subsequently harvested, and 10,000 cells were analysed in the acquisition. The analysis was performed using CellQuest software (Becton Dickinson Immunocytometry Systems).

### qPCR analysis

The transcriptional expression of *CDR1*, *CDR2* and *MDR1* in the tested *C. albicans* or *C. tropicalis* isolates was measured using qPCR as previously reported^55^. The primers used are shown in Supplementary Results Table S6. 18S rRNA served as the internal control in *C. albicans*, while *ACT1* was used as an internal control in *C. tropicalis*. The transcript levels of the detected genes were calculated using the formula 2^−ΔΔCT^.

### Statistical analysis

The experimental data were statistically analysed using Student’s t-test. The asterisks indicate critical levels of significance (*p < 0.05, **p < 0.01, and ***p < 0.001).

## Additional Information

**How to cite this article**: Xie, F. *et al*. Quinone derivatives isolated from the endolichenic fungus *Phialocephala fortinii* are Mdr1 modulators that combat azole resistance in *Candida albicans*. *Sci. Rep.* **6**, 33687; doi: 10.1038/srep33687 (2016).

## Supplementary Material

Supplementary Information

## Figures and Tables

**Table 1:** The synergic antifungal test of compounds 3 and 8 with FLC against azole-resistant *C. albicans* strains.

Strains	MIC_80_ (μg/ml)				FICI	Interpretation^a^	MIC_80_ (μg/ml)				FICI	Interpretation
	Alone		In combination				Alone		In combination			
	FLC	3	FLC	3			FLC	8	FLC	8		
24D	>256	>128	2	16	0.133	SYN	>256	>128	2	16	0.133	SYN
28I	>256	>128	2	16	0.133	SYN	>256	>128	2	16	0.133	SYN
CA10	>256	>128	2	16	0.133	SYN	>256	>128	2	16	0.133	SYN
CA406	>256	>128	2	16	0.133	SYN	>256	>128	2	16	0.133	SYN
CA417	>256	>128	2	16	0.133	SYN	>256	>128	2	16	0.133	SYN
CA631	>256	>128	2	16	0.133	SYN	>256	>128	2	16	0.133	SYN

MIC, minimum inhibitory concentration; FICI, fraction inhibited concentration index.

^a^SYN, synergism; IND, indifference. SYN was defined as a FICI of ≤0.5, antagonism was defined as a FICI of > 4.0, and indifference was defined as a FICI of >0.5 to 4.

**Table 2:** The cytotoxicity of compounds 3 and 8 against human normal cell lines.

Compound	IC_50_ (μg/ml)		
	HUVEC	HBE	RWPE-1
**3**	42.52 ± 1.02	16.44 ± 0.72	34.33 ± 1.25
**8**	80.38 ± 1.66	90.45 ± 1.57	87.16 ± 1.84
Amphotericin B	10.32 ± 0.89	22.45 ± 0.17	14.54 ± 0.07

**Table 3:** The susceptibility test of compound 3 alone and in combination against efflux pumps-deficient *C. albicans* strains by checkerboard microdilution assay and drug interaction analysis by FICI model.

Strains	Genotype	MIC_80_ (μg/ml)					
		Alone		In combination		FICI	Interpretation
		FLC	3	FLC	3		
DSY448	▵*cdr1::hisG-URA3-hisG/*▵*cdr1::hisG*	1	>128	0.03125	8	0.094	SYN
DSY653	▵*cdr2::hisG-URA3-hisG/*▵*cdr2::hisG*	0.5	>128	0.03125	8	0.125	SYN
DSY465	▵*mdr1::hisG-URA3-hisG/*▵*mdr1::hisG*	0.5	>128	0.125	64	0.750	IND
DSY659	▵*cdr1::hisG/*▵*cdr1::hisG* ▵*cdr2::hisG-URA3-hisG/*▵*cdr2::hisG*	0.25	>128	0.0156	8	0.125	SYN
YEM13	hyperexpressing *MDR1*	64	>128	2	16	0.156	SYN
YEM15	hyperexpressing *CDR1* and *CDR2*	64	>128	8	64	0.625	IND

**Table 4:** The susceptibility test of compound 8 alone and in combination against efflux pumps-deficient *C. albicans* strains by checkerboard microdilution assay and drug interaction analysis by FICI model.

Strains	Genotype	MIC_80_ (μg/ml)					
		Alone		In combination		FICI	Interpretation
		FLC	8	FLC	8		
DSY448	▵*cdr1::hisG-URA3-hisG/*▵*cdr1::hisG*	1	>128	0.0625	8	0.125	SYN
DSY653	▵*cdr2::hisG-URA3-hisG/*▵*cdr2::hisG*	0.5	>128	0.0625	8	0.188	SYN
DSY465	▵*mdr1::hisG-URA3-hisG/*▵*mdr1::hisG*	0.5	>128	0.125	8	0.313	SYN
DSY659	▵*cdr1::hisG/*▵*cdr1::hisG* ▵*cdr2::hisG-URA3-hisG/*▵*cdr2::hisG*	0.25	>128	0.0156	8	0.125	SYN
YEM13	hyperexpressing *MDR1*	64	>128	16	32	0.500	SYN
YEM15	hyperexpressing *CDR1* and *CDR2*	64	>128	8	64	0.625	IND
