# Supplementary material for: Quinone derivatives isolated from the endolichenic fungus Phialocephala fortinii are Mdr1 modulators that combat azole resistance in Candida albicans
Source: Sci Rep. 2016 Sep 21;6:33687. doi: 10.1038/srep33687 (PMC5030645; doi:10.1038/srep33687)
Supplement: Supplementary Information [file srep33687-s1.pdf]

Supplementary Results

Quinone derivatives isolated from the endolichenic fungus *Phialocephala fortinii* are Mdr1 modulators that combat azole resistance in *Candida albicans*

Fei Xie,[‡] Wenqiang Chang,[‡] Ming Zhang,[‡] Ying Li, Wei Li, Hongzhuo Shi, Sha Zheng, and Hongxiang Lou*

Department of Natural Product Chemistry, Key Lab of Chemical Biology of the Ministry of Education, Shandong University, No. 44 West Wenhua Road, Jinan City, Shandong Province, China

‡ These authors contributed equally to this work.

*Corresponding author: Hongxiang Lou.

Mailing address: School of Pharmaceutical Sciences, Shandong University, 44 West Wenhua Road, Jinan 250012, China.

Phone: 86-531-88382012. Fax: 86-531-88382019.

E-mail: louhongxiang@sdu.edu.cn

Contents

Structure elucidation of natural products **S3-S13**

Table S1. ^{1}H and ^{13}C NMR Data for Compounds **1-4** **S14**

Table S2. ^{1}H and ^{13}C NMR Data for Compounds **7-10** **S15**

Table S3. ^{1}H and ^{13}C NMR Data for Compounds **11-13** **S16**

Table S4. *In vitro* susceptibilities of compounds in *Phialocephala fortinii* and FLC acting alone and in combination against *C. albicans* by checkerboard microdilution assay. **S17**

Table S5. *C. albicans* strains used in this study. **S18**

Table S6. The primers used in this study. **S19**

Figure S1 The potent activity of the hit enhancing the efficacy of FLC against the azole-resistant *C. albicans* isolate 24D. **S20**

Figure S2−S87 X-ray crystallographic structure of compounds **2-4** and **7**, Key ^{1}H−^{1}H COSY and HMBC correlations for new compounds, NMR, and CD spectra of new compounds.

S21−S63

Structure elucidation of natural products

Palmarumycin P1 (**1**) was obtained as a pink powder. An HRESIMS analysis gave a molecular ion at *m/z* 359.0892 ($[M + Na]^+$; calcd 359.0890), which was consistent with the molecular formula $C_{20}H_{16}O_5$. The ^{1}H and ^{13}C NMR spectra (**Table S1**) showed signals for a 1, 2, 3-trisubstituted aromatic ring (δ_H 7.13, δ_C 117.2, CH-5; δ_H 7.25, δ_C 128.9, CH-6; δ_H 6.96, δ_C 116.2, CH-7), two oxymethine (δ_H 4.89, δ_C 62.9, CH-1; δ_H 3.82, δ_C 65.9, CH-2), one methylene (δ_H 2.23 and 2.07, δ_C 36.2, CH_2-3) and a set of signals characteristic of the dioxynaphthalene moiety. The correlations among these units were determined by the analysis of ^{1}H-^{1}H COSY and HMBC spectra (**Figure S2**). The ^{1}H–^{1}H COSY spectrum helped to identify the coupling systems H-1−H-2−H-3 and H-5−H-6−H-7 of structure **1**. The absolute configuration of **1** was determined as 1*R*, 2*S* by the contrary ECD Cotton effects [(Δε) 230 (−2.00)] (**Figure S8**) with those of compound **5** and **6**[1].

Palmarumycin P2 (**2**) was obtained as a pink crystals (MeOH). The molecular formula of compound **2** was determined to be $C_{21}H_{18}O_5$ by HRESIMS analysis (*m/z* 373.1048 ($[M + Na]^+$; calcd 373.1046)). The additional CH_2 in the molecular formula relative to that of **2** and analysis of the ^{1}H and ^{13}C NMR spectra (**Table S1**) indicated that the hydroxyl group of C-1 in **1** was changed to a methoxy group in **2**. This has also been supported by the HMBC correlation from OCH_3-1(δ_H 3.52) to C-1(73.0). The structure of compound **2** could be confirmed by other HMBC correlations (**Figure S9**). Comparison of the CD spectra (**Figure S16**) of compounds **5** and **6**[1], together with the single-crystal X-ray diffraction analysis with Cu Kα radiation (**Figure S10**), the absolute configuration of **2** was established as 1*R*, 2*S*.

Palmarumycin P3 (**3**), a pink crystals (MeOH), was assigned to have the same molecular formula $C_{20}H_{16}O_5$ as Palmarumycin P1 (**3**) on the basis of HRESIMS analysis (*m/z* 359.0890 ([M

+ $Na]^{+}$; calcd 359.0890)) and NMR data (**Table S1**). The 2D NMR data demonstrated that the compounds **1** and **3** have the same gross structure (**Figure S17**). While the C-1 and C-2 were shifted from δ_C 62.9 and 65.9 in **1** to δ_C 71.5 and 68.5 in **3**. Through optical rotation and CD spectra (**Figure S24**) and a single -crystal X-ray diffraction measurement (**Figure S18**), the absolute configuration of **3** could be determined as 1*S*, 2*S*.

Palmarumycin P4 (**4**) was obtained as a pink crystals (MeOH). HRESIMS (*m/z* 333.0761 ([M – $H]^{-}$; calcd 333.0759)) determined its molecular formula as $C_{20}H_{14}O_5$.

The NMR data (**Table S1**) showed the compound **4** was also a spirobisnaphthalenes, and its structure was confirmed by single-crystal X-ray diffraction study (**Figure S25**).

Phialocephalarin A (**7**), a yellow crystals (MeOH), has the molecular formula $C_{20}H_{16}O_7$ as determined by HRESIMS (*m/z* 367.0814 ([M – $H]^{-}$; calcd 367.0813)) and ^{13}C NMR data, revealing 13 degrees of unsaturation. Analysis of ^{1}H, ^{13}C and HSQC NMR spectra (**Table S2**) showed the presence of five exchangeable protons, two methylene, four methines (three oxygenated), one quaternary carbons, 12 aromatic/olefinic carbons (including three oxygenated carbons and three aromatic methine carbons), and one carbonyl carbon. These data accounted for all ^{1}H and ^{13}C NMR resonances and indicated a perylene quinone skeleton with six rings. The ^{1}H–^{1}H COSY spectrum revealed the coupling systems H-1–H-2–H-3 and H-2'–H-3'–H-4'. Through the HMBC spectrum (**Figure S31**), the planar structure of compound **7** was unambiguously established as depicted. The relative configuration of **7** was assigned by analysis of coupling constants of the protons and its NOESY data. In the NOESY spectrum, H-1 was correlated to H-3, which indicated theses protons were in the same orientation. While the NMR data could not provide sufficient information to confirm the complete structure, a single-crystal

X-ray diffraction experiment was performed using Cu K*α* radiation to clarify the uncertain structure details (**Figure S32**). Thus the absolute configuration of compound **7** was determined as 1*S*, 2*S*, 3*S*, 4*S*, 4'*S*.

Phialocephalarin B (**8**), a brown amorphous powder, HRESIMS (*m/z* 383.0761 ($[M – H]^-$; calcd 383.0762)) and ^{13}C NMR data indicated the molecular formula $C_{20}H_{16}O_8$. Analysis of its ^1H, ^{13}C and HSQC NMR spectra (**Table S2**) revealed similar structural features to those of **8**, except for the C-4' was shifted from δ_C 40.3 to δ_C 70.5. The analysis above indicated that one hydroxyl group attached to C-4', which was supported by detailed analysis of the HMBC spectrum of **8** (**Figure S40**).

Phialocephalarin C (**9**) was obtained as a brown amorphous powder with a molecular formula $C_{20}H_{18}O_9$ as determined by the same strategy as compound **7** and **8**. The ^1H, ^{13}C and HSQC NMR data (**Table S2**) were close to those of **8** except for the significantly downfield shifts of C-2 (δ_C 65.0 ppm in **9**; δ_C 50.6 ppm in **8**) and C-3 (δ_C 71.3 ppm in **9**; δ_C 53.4 ppm in **8**) were also different. These evidences indicated that two hydroxyl groups respectively were connected to C-2 and C-3 in **9** (**Figure S48**).

Phialocephalarin D (**10**) was isolated as a brown amorphous powder. HRESIMS and ^{13}C NMR spectra determined its molecular formula as $C_{20}H_{16}O_6$. Analysis of the ^1H, ^{13}C and HSQC data of **10** (**Table S2**) indicated that the presence of two carbonyl carbon and four methylene, one methines, one quaternary carbons, 12 aromatic/olefinic carbons (including three oxygenated carbons and three aromatic methine carbons), which showed that compound **10** was also a novel perylene quinone derivative as **7** (**Figure S56**).

In order to determine the Phialocephalarin B-D (**8-10**) configurations, the NOESY, optical

rotation and CD spectra were detected. From a biosynthetic standpoint, together with comparison of the CD spectra of compounds **7-10** (**Figure S39, S47, S55, S63**), the absolute configuration assignments of **8-10** could be determined (**Figure 1**).

Juglanone C (**11**) was obtained as a brown amorphous powder. Its molecular formula was determined as $C_{20}H_{18}O_5$ by HRESIMS with *m/z* 361.1055 $[M + Na]^+$ (calcd 361.1046). The ^1H and ^{13}C NMR together with HSQC data of 2 (**Table S3**) indicated one chelated phenolic H-atom (δ_H 12.43), two CH-O (δ_H 4.68, δ_C 66.5, C-4 and δ_H 5.74, δ_C 73.4, C-4') and four CH_2 groups (δ_H 2.45 and 2.57, δ_C 36.6, C-2; δ_H 1.86 and 2.12, δ_C 31.2, C-3; δ_H 2.75 and 3.12, δ_C 33.9, C-2';δ_H 2.27 and 2.36, δ_C 27.5, C-3') and six aromatic methines (δ_H 7.23, δ_C 119.8, CH-6; δ_H 7.56, δ_C 133.9, CH-7; δ_H 7.33, δ_C 115.6, CH-8; δ_H 7.09, δ_C 119.0, CH-5'; δ_H 7.53, δ_C 136.6, CH-6'; δ_H 6.95, δ_C 117.5, CH-7'). The NMR data demonstrated that compound **11** have the same gross structure with Juglanone A[2], which was confirmed by HMBC spectrum (**Figure S64**). In the NOESY spectrum of **11**, there is no correlation between H-4 and H4'. The CD spectra of **11** and Juglanone A were similar expect that compound **11** showed a negative Cotton effect at 226 nm (**Figure S71**). From a standpoint of biosynthesis, together with comparison of the CD spectra of compound **11** and Juglanone A, the absolute configuration of **11** was established as 4*S*, 4'*S*. Finally, the structure of compound **11** was unambiguously determined as depicted in **Figure 1**.

Juglanone D (**12**) was obtained as a brown amorphous powder. Its molecular formula was established as $C_{20}H_{20}O_5$ from HRESIMS (*m/z* 363.1201 ($[M + Na]^+$; calcd 363.1203)). The ^1H and ^{13}C NMR spectra of **12** (**Table S3**) indicated that it was also an tetralone dimmer like compound **11** except that the C-1 was changed from an C=O to C-OH at δ_C 60.0. In the NOESY spectrum of **12**, there is a correlation between H-1and H-4. The CD behavior and optical rotation of **12** (**Figure**

S79) were similar to those of **11**, which proved the absolute configuration at C-4 and C-4' were also assigned *S*. Further, the absolute configuration at C-1 was determined *R*. Thus, the structure of compound **12** was determined as shown in **Figure 1**.

Juglanone E (**13**) was isolated as a brown amorphous powder. The compound **13** was analyzed for $C_{20}H_{20}O_5$ by the same strategy as that of **12**. The IR and UV spectra showed the same absorptions which indicated the same skeleton as that of **12**. Careful comparison of their ^1H NMR data (**Table S3**) suggested that the H-atom signals due to an aromatic ring were changed. Further analysis of its ^{13}C NMR, HMQC and HMBC spectra showed that one subunit of **13** is 8-hydroxy-naphthalenol, as shown in **Figure S79**. NOESY correlations of H-1 with H-4' proved that they were in the same orientation. The absolute configuration of **13** was established as 1*S*, 4*S*, 4'*S* based on comparing the CD spectra (**Figure S87**) and optical rotation of compounds **11**, **12** and **13**.

The known compounds were identified as CJ-12,371(**5**), Palmarumycins CP_{19} (**6**), (-)-Regiolone (**14**), Sclerone (**15**), by comparison of their spectroscopic data with previously reported data. [1,3.4]

Fraction D (1.2 g) was subjected to a Sephadex LH-20 CC and eluted with CH_2Cl_2/MEOH (1:1) to obtain five subfractions (D_1-D_5). Fraction D_5 (146.1mg) was fractionated using MPLC (ODS, MeOH/H_2O from 50:50 to 100:0) to yield six subfractions (D_{5A}–D_{5F}). Then, **14** (t_R 17.2 min; 49.6 mg) was isolated from D_{5A} (87.4 mg) by HPLC (51% MeOH/H_2O, 1.5 mL/min). D_{5B} (16.7 mg) was purified by HPLC (81% MeOH/H_2O, 1.5 mL/min) to yield **6** (t_R 23.0 min; 4.1 mg).

Fraction E (1.2 g) was separated using Sephadex LH-20 CC by elution with CH_2Cl_2/MeOH (1:1) to afford four subfractions (E_1–E_4). Fraction E_4 (212.4 mg) was separated to subfractions E_{4A}–E_{4G} by MPLC (ODS, MeOH/H_2O from 30:70 to 100:0). Fraction E_{4A} (24.1 mg) was further purified

using HPLC (30% MeOH /H_2O, 1.5 mL/min) to yield **15** (1.8 mg, t_R = 27.2 min). Separation of fraction E_{4E} following a similar procedure to that used for fraction D_{5A} afforded **2** (HPLC, 67% MeOH/H_2O, 1.5 mL/min; 2.7 mg, t_R = 40.7 min). Further purification of E_{4D} (31.9 mg) with HPLC (60% MeOH/H_2O, 1.5 mL/min) yielded **11** (t_R 17.0 min; 1.4 mg) and **10** (t_R 30.0 min; 3.9 mg). Fraction E_{4F} was purified to give **4** (t_R 23.0 min; 2.6 mg) and **5** (t_R 19.0 min; 1.0 mg) by using HPLC (80% MeOH/H_2O, 1.5 mL/min).

Fraction F (1.6 g) was fractionated using MPLC (ODS, MeOH/H_2O from 40:60 to 100:0) to yield three subfractions (F_1–F_3). Fraction F_1 (21.9 mg) was subjected to HPLC using 50% aqueous MeOH (1.5 mL/min) to yield **13** (1.2 mg, t_R = 32.0 min). Fraction F_2 (56.0 mg) was purified by HPLC using MeOH/H_2O (70:30, 1.5 mL/min) to afford **12** (1.8 mg, t_R = 15.1 min), **1** (1.4 mg, t_R = 23.3 min) and **3** (38.0 mg, t_R = 30.0 min).

Fraction G (2.2 g) was also separated using Sephadex LH-20 CC by elution with CH_2Cl_2/MeOH (1:1) to afford three subfractions (G_1-G_3). Fraction G3 was further purified using HPLC(40% MeOH/H_2O, 1.5 mL/min) afforded **7** (10.0mg, t_R = 35.5 min). Fraction H (3.9 g) was purified to give **8** (39.1 mg; t_R = 15.0 min) and **9** (1.9 mg; t_R = 18.7 min) by using HPLC (25% MeOH/H_2O, 1.5 mL/min).

*Palmarumycin P1 (**1**):* pink powder; $[\alpha]_D^{20}$ –26.0 (c 0.1, MeOH); ECD (MeOH) λ_{max} ($\Delta\varepsilon$) 230 (–2.00); UV (MeOH) λ_{max} (log ε) 227 (3.97), 285 (3.15), 300 (3.08), 315 (3.00), 327 (2.96) nm; IR (KBr) v_{max} 3375, 2921, 2851, 1635, 1605, 1582, 1456, 1410, 1377, 1269, 1165, 1122, 1059, 955, 746 cm^{-1}; for ^1H and ^{13}C NMR data, see Table S1; HRESIMS *m/z* 359.0892 $[M + Na]^+$ (calcd for $C_{20}H_{16}O_5Na$ 359.0890).

*Palmarumycin P2 (**2**):* pink crystals; mp > 250 ℃; $[\alpha]_D^{20}$ –89.1 (c 0.1, MeOH); ECD (MeOH)

λ_{max} ($\Delta\varepsilon$) 227 (–9.16); UV (MeOH) λ_{max} (log ε) 227 (4.79), 287 (3.92), 300 (3.86), 315 (3.77), 327 (3.43) nm; IR (KBr) v_{max} 3226, 2938, 2837, 1633, 1604, 1471, 1410, 1377, 1323, 1272, 1124, 1054, 940, 753 cm^{-1}; for ^1H and ^{13}C NMR data, see Table S1; HRESIMS *m/z* 373.1048 $[M + Na]^+$ (calcd for $C_{21}H_{18}O_5Na$ 373.1046).

*Palmarumycin P3 (**3**):* pink crystals; mp > 250 ℃; $[\alpha]_D^{20}$ +1.2 (c 0.05, MeOH); ECD (MeOH) λ_{max} ($\Delta\varepsilon$) 226 (+1.83); UV (MeOH) λ_{max} (log ε) 227 (4.41), 287 (3.61), 300 (3.60), 315 (3.51), 327 (3.30) nm; IR (KBr) v_{max} 3422, 3370, 3005, 2924, 2853, 1634, 1607, 1462, 1413, 1379, 1270, 1119, 1063, 943, 756 cm^{-1}; for ^1H and ^{13}C NMR data, see Table S1; HRESIMS *m/z* 359.0890 $[M + Na]^+$ (calcd for $C_{20}H_{16}O_5Na$ 359.0890).

*Palmarumycin P4 (**4**):* pink crystals; mp > 250 ℃; UV (MeOH) λ_{max} (log ε) 227 (4.41), 287 (3.61), 300 (3.60), 315 (3.51), 327 (3.30) nm; IR (KBr) v_{max} 3422, 3370, 3005, 2924, 2853, 1634, 1607, 1462, 1413, 1379, 1270, 1119, 1063, 943, 756 cm^{-1}; for ^1H and ^{13}C NMR data, see Table S1; HRESIMS *m/z* 333.0761 $[M – H]^-$ (calcd for $C_{20}H_{13}O_5$ 333.0759).

*Phialocephalarin A (**7**):* yellow crystals; mp > 250 ℃; $[\alpha]_D^{20}$ –291.2 (c 0.006, MeOH); ECD (MeOH) λ_{max} ($\Delta\varepsilon$) 209 (+18.85), 236 (–10.00), 260 (–4.69), 309 (–3.51); UV (MeOH) λ_{max} (log ε) 210 (4.41), 260 (4.12), 278 (4.15), 382 (3.34) nm; IR (KBr) v_{max} 3354, 2957, 1639, 1596, 1458, 1361, 1281, 1167, 1023 cm^{-1}; for ^1H and ^{13}C NMR data, see Table S2; HRESIMS *m/z* 367.0814 ($[M – H]^-$ (calcd for $C_{20}H_{15}O_8$ 367.0813).

*Phialocephalarin B (**8**):* brown amorphous powder; $[\alpha]_D^{20}$ –365.4 (c 0.1, MeOH); ECD (MeOH) λ_{max} ($\Delta\varepsilon$) 209 (+34.69), 236 (–18.28), 261 (–10.10), 308 (–8.52); UV (MeOH) λ_{max} (log ε) 210 (4.60), 261 (4.35), 280 (4.39), 382 (3.49) nm; IR (KBr) v_{max} 3540, 3211, 1638, 1493, 1393, 1336, 1291, 1248, 1181, 1038 cm^{-1}; for ^1H and ^{13}C NMR data, see Table S2; HRESIMS *m/z* 383.0761

([M – H]$^-$ (calcd for $C_{20}H_{15}O_8$ 383.0762).

*Phialocephalarin C (**9**):* brown amorphous powder; $[\alpha]_D^{20}$ –136.0 (c 0.01, MeOH); ECD (MeOH) λ_{max} ($\Delta\varepsilon$) 207 (+10.23), 236 (–8.33), 259 (–2.77), 311 (–2.56); UV (MeOH) λ_{max} (log ε) 210 (4.36), 261 (3.93), 280 (3.97), 382 (3.03) nm; IR (KBr) v_{max} 3439, 2919, 1724, 1628, 1586, 1434, 1368, 1239, 1042 cm^{-1}; for ^{1}H and ^{13}C NMR data, see Table S2; HRESIMS *m/z* 401.0871 [M – H]$^-$ (calcd for $C_{20}H_{17}O_9$ 401.0867).

*Phialocephalarin D (**10**):* brown amorphous powder; $[\alpha]_D^{20}$ –282.8 (c 0.025, MeOH); ECD (MeOH) λ_{max} ($\Delta\varepsilon$) 217 (+7.07), 259 (–5.29); UV (MeOH) λ_{max} (log ε) 216 (4.12), 250 (4.02), 259 (4.00), 340 (3.29) nm; IR (KBr) v_{max} 3392, 2922, 1627, 1544, 1451, 1336, 1244, 1169, 1024 cm^{-1}; for ^{1}H and ^{13}C NMR data, see Table S2; HRESIMS *m/z* 351.0855 [M – H]$^-$ (calcd for $C_{20}H_{15}O_6$ 351.0864).

*Juglanone C (**11**):* brown amorphous powder; $[\alpha]_D^{20}$ –62.8 (*c* 0.05, MeOH); ECD (MeOH) λ_{max} ($\Delta\varepsilon$) 226 (–4.67), 245 (+3.65), 263 (–5.53), 310 (–1.64); UV (MeOH) λ_{max} (log ε) 213 (3.46), 258 (3.03), 321 (2.62) nm; IR (KBr) v_{max} 3396, 2924, 2854, 1635, 1588, 1454, 1243, 1162, 1098, 805, 747 cm^{-1}; for ^{1}H and ^{13}C NMR data, see Table S3; HRESIMS *m/z* 361.1055 [M + Na]$^+$ (calcd for $C_{20}H_{18}O_5Na$ 361.1046).

*Juglanone D (**12**):* brown amorphous powder; $[\alpha]_D^{20}$ –108.4 (*c* 0.03, MeOH); ECD (MeOH) λ_{max} ($\Delta\varepsilon$) 226 (–2.18), 265 (–3.33); UV (MeOH) λ_{max} (log ε) 212 (3.26), 257 (2.83), 330 (2.39) nm; IR (KBr) v_{max} 3401, 2925, 2855, 1727, 1639, 1582, 1456, 1248, 1165, 1099, 806, 744 cm^{-1}; for ^{1}H and ^{13}C NMR data, see Table S3; HRESIMS *m/z* 363.1201 [M + Na]$^+$ (calcd for $C_{20}H_{20}O_5Na$ 363.1203).

Juglanone E (**13**)*:* brown amorphous powder; $[\alpha]_D^{20}$ –53.6 (*c* 0.05, MeOH); ECD (MeOH) λ_{max} ($\Delta\varepsilon$) 265 (–2.13), 332 (–0.56); UV (MeOH) λ_{max} (log ε) 213 (4.31), 257 (3.76), 330 (2.43) nm; IR (KBr) v_{max} 3376, 2925, 2852, 1633, 1582, 1454, 1245, 1162, 1011, 799, 745 cm^{-1}; for ^1H and ^{13}C NMR data, see Table S3; HRESIMS *m/z* 363.1204 $[M + Na]^+$ (calcd for $C_{20}H_{20}O_5Na$ 363.1203).

X-ray Crystallographic Analysis of Compound 2. $C_{21}H_{17}O_5$, M_r = 349.35, monoclinic system, space group *P2(1)*; unit cell dimensions were determined to be *a* = 11.7030(6) Å, *b* = 9.1801(5) Å, *c* = 16.8493(8) Å, *V* = 1714.96(15) $Å^3$, *Z* = 4, D_{calcd} = 1.353 Mg/mm^3, μ (Cu K*α*) = 0.797 mm^{-1}, *F* (000) = 732, *T* = 299(2) K, 31389 reflections measured, 5731 unique reflections (R_{int} = 0.05) which were used in all calculations. A crystal of dimensions 0.30 × 0.15 × 0.10 mm^3 was selected for measurements on a Bruker D8 venture diffractometer employing APEX II CCD using Cu K*α* radiation. APEX2 Software Suite was used for cell refinement and data reduction. The structure was refined with full-matrix least-squares calculations on F^2 using SHELXL-2014/7 (Sheldrick, 2014).The final stage converged to R_1 = 0.0373 (wR_2 = 0.0820) for 4696 observed reflections [with I > 2σ(I)] and 473 variable parameters, R_1 = 0.0516 (wR_2 = 0.0896) for all unique reflections, and goodness-of-fit = 1.039. The Flack parameter is 0.02(10).

X-ray Crystallographic Analysis of Compound 3. $C_{21}H_{20}O_6$, M_r = 368.37, monoclinic system, space group *P2(1)*; unit cell dimensions were determined to be *a* = 4.98480(10) Å, *b* = 21.1663(6) Å, *c* = 8.4299(2) Å, *V* = 872.38(4) $Å^3$, *Z* = 2, D_{calcd} = 1.402 Mg/mm^3, μ (Cu K*α*) = 0.854 mm^{-1}, *F* (000) = 388, *T* = 300(2) K, 11835 reflections measured, 3151 unique reflections (R_{int} = 0.0367) which were used in all calculations. A crystal of dimensions 0.46 × 0.17 × 0.13 mm^3 was selected for measurements on a Bruker D8 venture diffractometer employing APEX II CCD using Cu K*α* radiation. APEX2 Software Suite was used for cell refinement and data reduction. The structure

was refined with full-matrix least-squares calculations on F^2 using SHELXL-2014/7 (Sheldrick, 2014).The final stage converged to R_1 = 0.0336 (wR_2 = 0.0852) for 2983 observed reflections [with I > 2σ(I)] and 256 variable parameters, R_1 = 0.0361 (wR_2 = 0.0873) for all unique reflections, and goodness-of-fit = 1.088. The Flack parameter is 0.17(7).

X-ray Crystallographic Analysis of Compound 4. $C_{20}H_{14}O_5$, M_r = 334.31, monoclinic system, space group *P2(1)*; unit cell dimensions were determined to be *a* = 9.3210(7) Å, *b* = 7.9839(6) Å, *c* = 20.5119(15) Å, *V* = 1518.5(2) Å^3, *Z* = 4, D_{calcd} = 1.462 Mg/mm^3, μ (Cu K*α*) = 0.876 mm^{-1}, *F* (000) = 696, *T* = 299(2) K, 20936 reflections measured, 2683 unique reflections (R_{int} = 0.0457) which were used in all calculations. A crystal of dimensions 0.26 × 0.18 × 0.12 mm^3 was selected for measurements on a Bruker D8 venture diffractometer employing APEX II CCD using Cu K*α* radiation. APEX2 Software Suite was used for cell refinement and data reduction. The structure was refined with full-matrix least-squares calculations on F^2 using SHELXL-2014/7 (Sheldrick, 2014).The final stage converged to R_1 = 0.0391 (wR_2 = 0.0920) for 2105 observed reflections [with I > 2σ(I)] and 234 variable parameters, R_1 = 0.0544 (wR_2 = 0.1006) for all unique reflections, and goodness-of-fit = 1.021.

X-ray Crystallographic Analysis of Compound 7. $C_{41}H_{41}O_{18}$, M_r = 821.74, monoclinic system, space group *P2(1)*; unit cell dimensions were determined to be *a* = 12.7755(7) Å, *b* = 7.2748(3) Å, *c* = 20.3792(13) Å, *V* = 1811.55(17) Å^3, *Z* = 2, D_{calcd} = 1.506 Mg/mm^3, μ (Cu K*α*) = 1.014 mm^{-1}, *F* (000) = 862, *T* = 293(2) K, 5942 reflections measured, 4043 unique reflections (R_{int} = 0.0409) which were used in all calculations. A crystal of dimensions 0.26 × 0.12 × 0.10 mm^3 was selected for measurements on a Bruker APEX DUO diffractometer employing APEX II CCD using Cu K*α* radiation. APEX2 Software Suite was used for cell refinement and data reduction.

The structure was refined with full-matrix least-squares calculations on F^2 using SHELXL-97 (Sheldrick, 1997). The final stage converged to R_1 = 0.0564 (wR_2 = 0.1014) for 2477 observed reflections [with $I > 2\sigma(I)$] and 534 variable parameters, R_1 = 0.1034 (wR_2 = 0.1147) for all unique reflections, and goodness-of-fit = 1.043. The Flack parameter is 0.3(4).

Crystallographic data for these structures have been deposited with the Cambridge Crystallographic Data Centre as CCDC 1441582 for **2**, CCDC 1441583 for **3**, CCDC 1441584 for **4**, and CCDC 1441585 for **7**. Copies of the data can be obtained free of charge at www.ccdc.cam.ac.uk/conts/retrieving.html (or from the Cambridge Crystallographic Data Centre, 12 Union Road, Cambridge CB2 1EZ, UK; fax: (+44) 1223-336-033; e-mail: deposit@ccdc.cam.ac.uk).

Table S1. ^{1}H (600 MHz) and ^{13}C (150 MHz) NMR Data for Compounds 1-4 in DMSO-d_6

Position	**1**		**2**		**3**		**4**	
	d_C, type	d_H, mult. (J in Hz)	d_C, type	d_H, mult. (J in Hz)	d_C, type	d_H, mult. (J in Hz)	d_C, type	d_H, mult. (J in Hz)
1	62.9, CH	4.89, br s	73.0, CH	4.62, d (3.0)	71.5, CH	4.78, d (7.2)	204.1, C	
2	65.9, CH	3.82, br d(10.8)	66.7, CH	3.93, br d (10.2)	68.5, CH	3.94, t (7.8)	34.0, CH_2	2.77, t (6.0)
3a	32.8, CH_2	2.24, m	33.7, CH_2	2.22, t (12.6)	36.2, CH_2	2.34, dd (13.2, 3.6)	28.9, CH_2	2.38, t(6.0)
3b		2.07, m		2.12, dd (12.6, 3.6)		1.93, dd (13.2, 10.8)		
4	101.2, C		101.0, C		100.0, C		98.5, C	
5	117.6, CH	7.13, d (7.8)	118.0, CH	7.13, d (7.8)	117.7, CH	7.17, d (7.8)	116.6, CH	7.19, m
6	128.9, CH	7.25, t (7.8)	129.3, CH	7.27, d (7.8)	128.8, CH	7.27, t (7.8)	121.5, CH	7.19, m
7	116.1, CH	6.96, d (7.8)	116.1, CH	6.99, d (7.8)	116.9, CH	6.93, d (7.8)	147.3, C	
8	155.6, C		155.5, C		156.4, C		150.3, C	
9	125.5, C		123.7, C		123.9, C		115.5, C	
10	135.2, C		135.3, C		135.1, C		130.1, C	
1'	147.8, C		147.7, C		147.5, C		147.2, C	
2'	109.3, CH	7.01, d (7.8)	109.4, CH	7.02, d (7.8)	109.3, CH	6.98, t (8.4)	109.4, CH	7.06, d (7.8)
3'	127.7, CH	7.51, m	127.8, CH	7.50, q (8.4,)	127.7, CH	7.51, m	127.8, CH	7.52, t (7.8)
4'	120.4, CH	7.61, m	120.5, CH	7.59, dd (8.4, 3.0)	120.4, CH	7.59, d (8.4)	120.7, CH	7.62, d (7.8)
5'	120.4, CH	7.61, m	120.5, CH	7.59, dd (8.4, 3.0)	120.4, CH	7.59, d (8.4)	120.7, CH	7.62, d (7.8)
6'	127.7, CH	7.51, m	127.8, CH	7.50, q (8.4,)	127.7, CH	7.51, m	127.8, CH	7.52, t (7.8)
7'	109.4, CH	6.93, d (7.8)	109.5, CH		109.5, CH	6.98, t (8.4)	109.4, CH	6.98, t (8.4)
8'	147.6, C		147.7, C		147.5, C		147.2, C	
9'	112.9, C		112.9, C		112.9, C		112.8, C	
10'	133.8, C		133.8, C		133.7, C		133.7, C	
OCH_3-1			59.7, CH_3	3.52, s				
HO-C(8)		9.75, s		9.93, s		9.83, br s		

Table S2. ^{1}H (600 MHz) and ^{13}C (150 MHz) NMR Data for Compounds 7-10 in DMSO-d_6

Position	**7**		**8**		**9**		**10**	
	d_C, type	d_H, mult. (*J* in Hz)	d_C, type	d_H, mult. (*J* in Hz)	d_C, type	d_H, mult. (*J* in Hz)	d_C, type	d_H, mult. (*J* in Hz)
1	65.8, CH	5.32, s	66.5, CH	5.29, d (1.8)	69.8, CH	4.21, s	205.9, C	
2a	54.1, CH	3.65, m	50.6, CH	3.45, q (2.4)	65.0, CH	4.85, s	33.4, CH_2	3.06, m
2b								2.59, m
3a	54.4, CH	3.65, m	53.4, CH	3.65, d (4.8)	71.3, CH	4.36, d (3.0)	32.2, CH_2	2.39, m
3b								2.14, m
4	66.9, C		69.4, C		69.7, C		66.2, C	
5	124.1, C		123.6, C		123.8, C		123.5, C	
6	124.3, CH	7.51, d (8.4)	124.1, CH	7.49, d (8.4)	124.3, CH	7.47, d (8.4)	132.7, CH	7.93, d (8.4)
7	116.3, CH	6.84, d (8.4)	116.0, CH	6.77, d (8.4)	114.5, CH	6.87, d (8.4)	117.4, CH	7.02, d (8.4)
8	156.0, C		156.5, C		156.2, C		160.8, C	
9	118.4, C		120.1, C		122.4, C		114.0, C	
10	133.0, C		132.3, C		132.9, C		140.4, C	
1'	206.1, C		206.5, C		206.3, C		206.1, C	
2'a	37.2, CH_2	2.91, m	33.3, CH_2	3.12, m	33.3, CH_2	3.12, m	37.3, CH_2	2.81, m
2'b		2.68, m		2.60, m		2.64, m		2.70, m
3'a	21.9, CH_2	2.44, m	28.2, CH_2	2.56, m	28.1, CH_2	2.66, m	21.0, CH_2	2.28, m
3'b		2.21, m		2.35, m		2.20, m		2.14, m
4'	40.3, CH	3.03, dd (12.0, 4.4)	70.5, C		71.8, C		42.8, CH	2.93, dd (12.0, 4.2)
5'	124.1, C		123.7, C		124.6, C		123.1, C	
6'	117.2, CH	7.47, s	117.4, CH	7.43, s	117.6, CH	7.45, s	117.4, CH	7.53, s
7'	144.2, C		145.6, C		145.6, C		144.4, C	
8'	149.9, C		149.6, C		149.0, C		150.2, C	
9'	116.7, C		115.5, C		115.5, C		116.6, C	
10'	128.6, C		128.9, C		128.2, C		129.7, C	
HO-C(4)		5.21, s		5.35, s		5.41, s		5.15, s
HO-C(8)								12.68, s
HO-C(8')		12.56, s		12.64, s				

Table S3. ^{1}H (600 MHz) and ^{13}C (150 MHz) NMR Data for Compounds 11-13 in DMSO-*d*$_6$

Position	11		12		13	
	d$_C$, type	*d*$_H$, mult. (*J* in Hz)	*d*$_C$, type	*d*$_H$, mult. (*J* in Hz)	*d*$_C$, type	*d*$_H$, mult. (*J* in Hz)
1	195.61, C		60.0, CH	4.83, d (2.4)	59.7, CH	4.71, d (3.0)
2a	36.6, CH_2	2.57, m	29.8, CH_2	1.84, m	26.2, CH_2	1.93, m
2b		2.45, m		1.68, m		1.61, m
3a	31.2, CH_2	2.12, m	26.8, CH_2	1.96, m	26.0, CH_2	2.10, m
3b		1.86, m		1.77, m		1.59, m
4	66.5, CH	4.68, m	67.9, CH	4.45, m	65.1, CH	4.52, d (3.0)
5	156.5, C		155.3, C		122.4, CH	6.94, d (7.8)
6	119.8, CH	7.23, d (7.8)	119.4, CH	7.18, d (7.8)	127.9, CH	7.24, t (7.8)
7	133.9, CH	7.56, t (7.8)	127.7, CH	7.25, t (7.8)	112.3, CH	7.13, d (7.8)
8	115.6, CH	7.33, d (7.8)	111.4, CH	7.09, d (7.8)	155.1, C	
9	149.8, C		143.3, C		128.1, C	
10	121.8, C		127.9, C		140.1, C	
1'	205.1, C		204.7, C		205.3, C	
2'a	33.9, CH_2	3.12, m	34.8, CH_2	2.89, m	33.9, CH_2	3.13, m
2'b		2.75, m		2.84, m		2.70, m
3'a	27.5, CH_2	2.36, m	27.8, CH_2	2.38, m	27.0, CH_2	2.37, m
3'b		2.27, m		2.16, m		2.34, m
4'	73.4, CH	5.74, m	72.6, CH	5.73, dd (8.4, 3.0)	72.3, CH	5.72, t (4.2)
5'	119.0, CH	7.09, d (7.8)	118.1, CH	7.33, d (7.8)	118.8, CH	7.00, d (7.8)
6'	136.6, CH	7.53, t (7.8)	136.9, CH	7.57, t (7.8)	136.9, CH	7.57, t (7.8)
7'	117.5, CH	6.95, d (7.8)	116.8, CH	6.93, d (7.8)	117.3, CH	6.96, d (7.8)
8'	161.6, C		161.6, C		161.7, C	
9'	115.5, C		115.4, C		115.5, C	
10'	142.6, C		144.3, C		143.3, C	
HO-C(8')		12.43, s		12.43, s		12.46, s

Table S4. ***In vitro*** **susceptibilities of compounds in** ***Phialocephala fortinii*** **and FLC acting alone and in combination against** ***C. albicans*** **by checkerboard microdilution assay**

Compounds	MIC$_{80}$ (μg/ml)					
	Alone		In combination		FICI	Interpretation[a]
	Azole	Compounds	Azole	Compounds		
**1**	>256	>128	2	32	<0.258	SYN
**2**	>256	>128	2	32	<0.258	SYN
**3**	>256	>128	2	16	<0.133	SYN
**4**	>256	>128	1	32	<0.254	SYN
**5**	>256	>128	1	32	<0.254	SYN
**6**	>256	>128	4	32	<0.266	SYN
**7**	>256	>128	1	16	<0.129	SYN
**8**	>256	>128	2	16	<0.133	SYN
**9**	>256	>128	1	16	<0.129	SYN
**10**	>256	>128	1	16	<0.129	SYN
**11**	>256	>128	>128	>64	>1	IND
**12**	>256	>128	>128	>64	>1	IND
**13**	>256	>128	>128	>64	>1	IND
**14**	>256	>128	>128	>64	>1	IND
**15**	>256	>128	>128	>64	>1	IND

MIC, minimum inhibitory concentration; FICI, fraction inhibited concentration index.

[a] SYN, synergism; IND, indifference. SYN was defined as a FICI of ≤0.5, antagonism was defined as a FICI of >4.0.

Table S5. *C. albicans* strains used in this study

Strains	Genotype	Source
DSY448	△*cdr1::hisG-URA3-hisG/*△*cdr1::hisG*	Reference 5
DSY653	△*cdr2::hisG-URA3-hisG/*△*cdr2::hisG*	Reference 6
DSY465	△*mdr1::hisG-URA3-hisG/*△*mdr1::hisG*	Reference 5
DSY659	△*cdr1::hisG/*△*cdr1::hisG* △*cdr2::hisG-URA3-hisG/*△*cdr2::hisG*	Reference 6
DSY1050	△*cdr1::hisG/*△*cdr1::hisG* △*cdr2::hisG/*△*cdr2::hisG* △*mdr1::hisG-URA3-hisG/*△*mdr1::hisG*	Reference 7
YEM13	hyperexpressing *MDR1*	Reference 8
YEM15	hyperexpressing *CDR1* and *CDR2*	Reference 8
24D	*C. albicans* FLC-resistant isolate	From hospital of Stomatology in Shandong university
28I	*C. albicans* FLC-resistant isolate	From hospital of Stomatology in Shandong university
CA10	*C. albicans* FLC-resistant isolate	From hospital of Stomatology in Shandong university
CA406	*C. albicans* FLC-resistant isolate	From hospital of Stomatology in Shandong university
CA417	*C. albicans* FLC-resistant isolate	From hospital of Stomatology in Shandong university
CA631	*C. albicans* FLC-resistant isolate	From hospital of Stomatology in Shandong university
NPC-T001	*C. tropicalis* FLC-resistant isolate	From hospital of Stomatology in Shandong university

Table S6. The primers used in this study.

Primers	Sequences (5'-3')
18S-F	AATTACCCAATCCCGACAC
18S-R	TGCAACAACTTTAATATACGC
CDR1-F	TAACACTTATGGTTTCCACAT
CDR1-R	AGCATAAGTTTCTCTGTCGA
CDR2-F	GAGTGTTGGTGATACTTTGG
CDR2-R	CACTCAAAGAAGCTTCAGCA
MDR1-F	AGATAATCAAGGTGAACCCAA
MDR1-R	GCTGATCCCATATAAACTGAA
CT-*ACT1*-F	ATGGACGGGGGTATGTTTCA
CT-*ACT1*-R	GACATAAGTAATTTCCAATGTG
CT-*MDR1*-F	CCCAGAAGTTTTCATTCCA
CT-*MDR1*-R	CCCCAAGCAACAGGATAAT

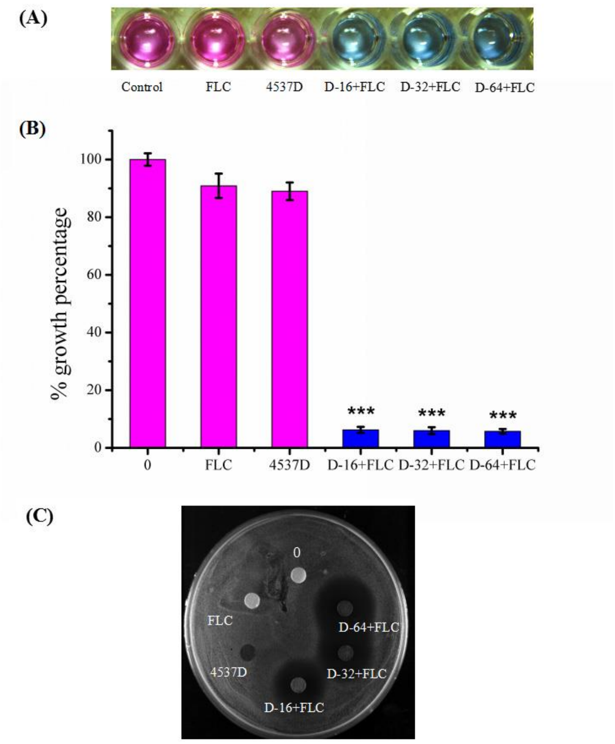

Figure S1. The potent activity of the hit enhancing the efficacy of FLC against the azole-resistant *C. albicans* isolate 24D. (**A**,**B**) The Alarm Blue assay results. Cells were stained with Alamar Blue for 2 hours in dark after treated with drugs for 48h, the supernatant would become pink when the cells proliferate, otherwise turn into blue (**A**) and the growth of *C.albicans* was measured with a spectrophotometer at 570 nm(**B**). The test was performed in quadruplicate. (**C**) The disk diffusion assay results. The assays were performed by plating the individual test organism 24D (1.5×10^6) on Mueller Hinton agar (MHA) medium supplemented with 2% glucose. Cellulose disks impregnated with FLC (4 μg), extrct (64 μg) and combination of each agent (4 μg FLC and 16 μg extrct; 4 μg FLC and 32 μg extrct; 4 μg FLC and 64 μg extrct) were placed onto MHA agar plates. Each plate was incubated at 30 ℃ for 48 h for the agar diffusion assay.

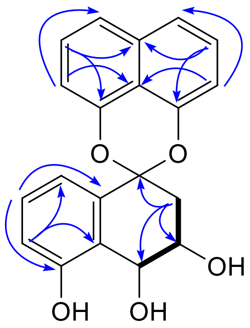

Figure S2. Key ^{1}H–^{1}H COSY (bold lines), HMBC (H→C) correlations for compound **1**.

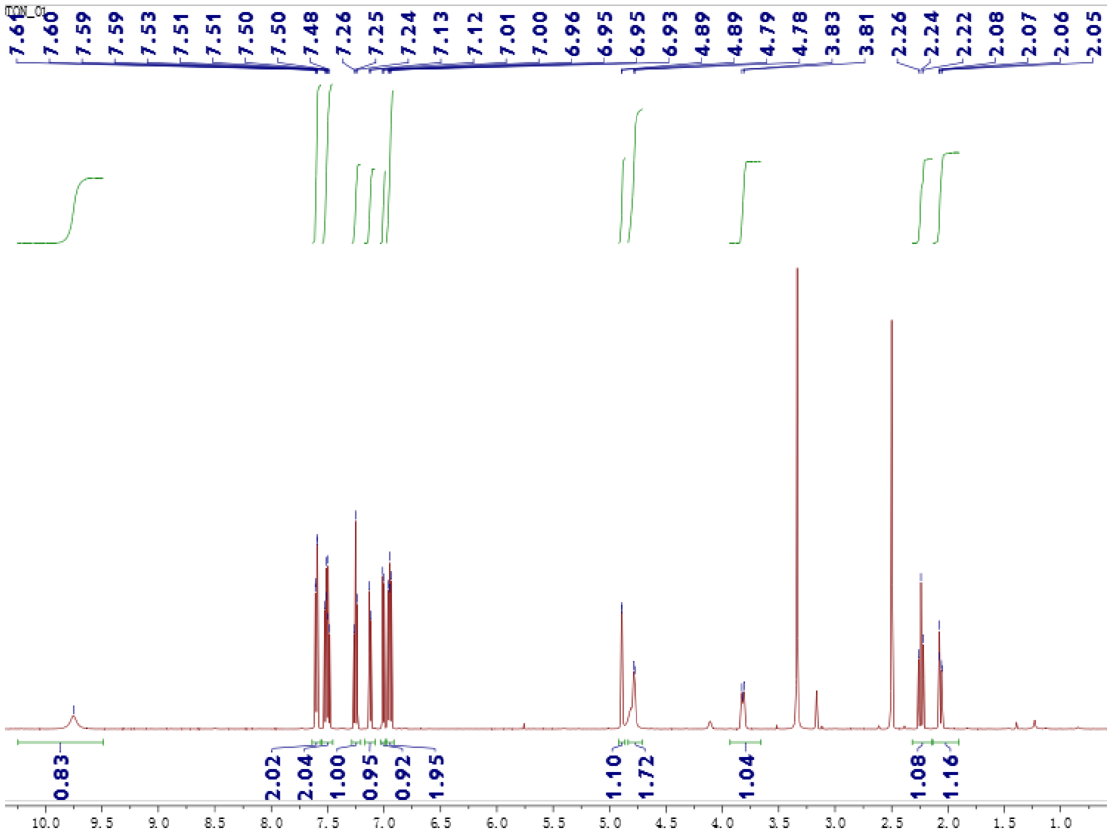

Figure S3. ^{1}H NMR spectrum (600 MHz) of **1** in DMSO-d_6.

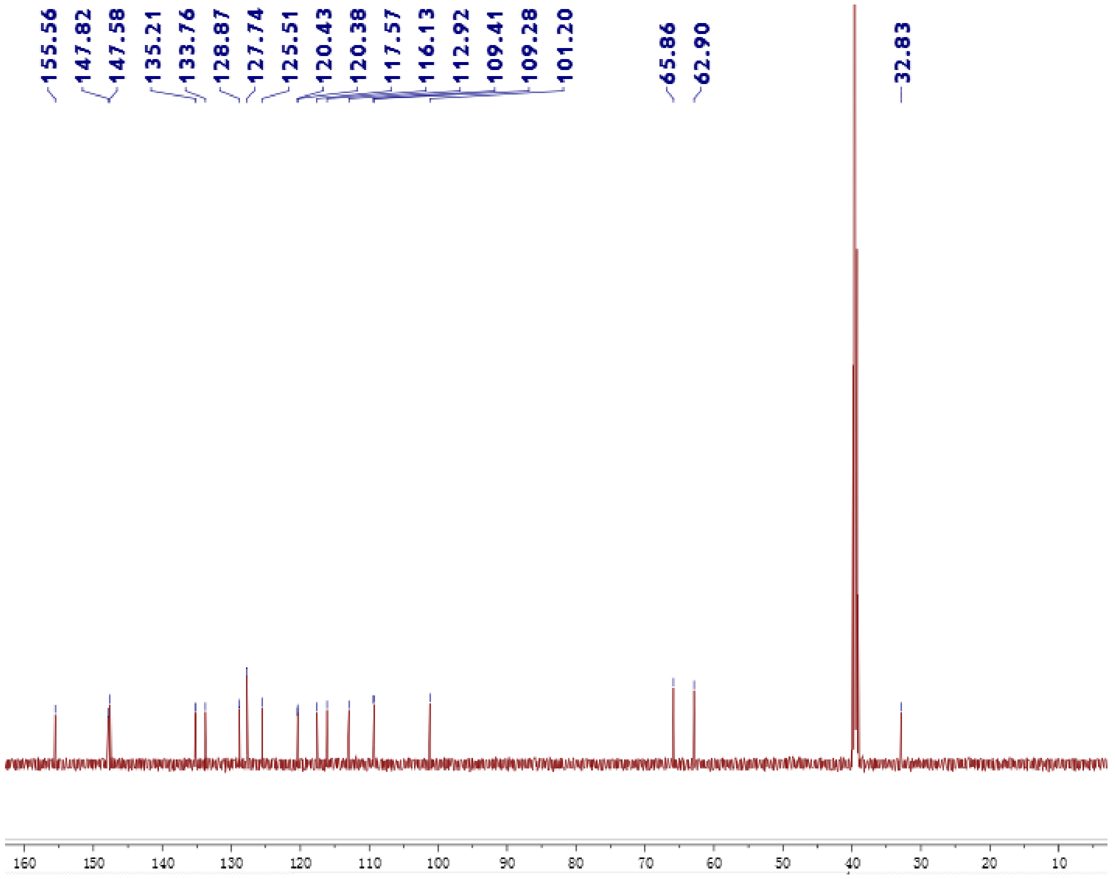

Figure S4. ^{13}C NMR spectrum (150 MHz) of **1** in DMSO-d_6.

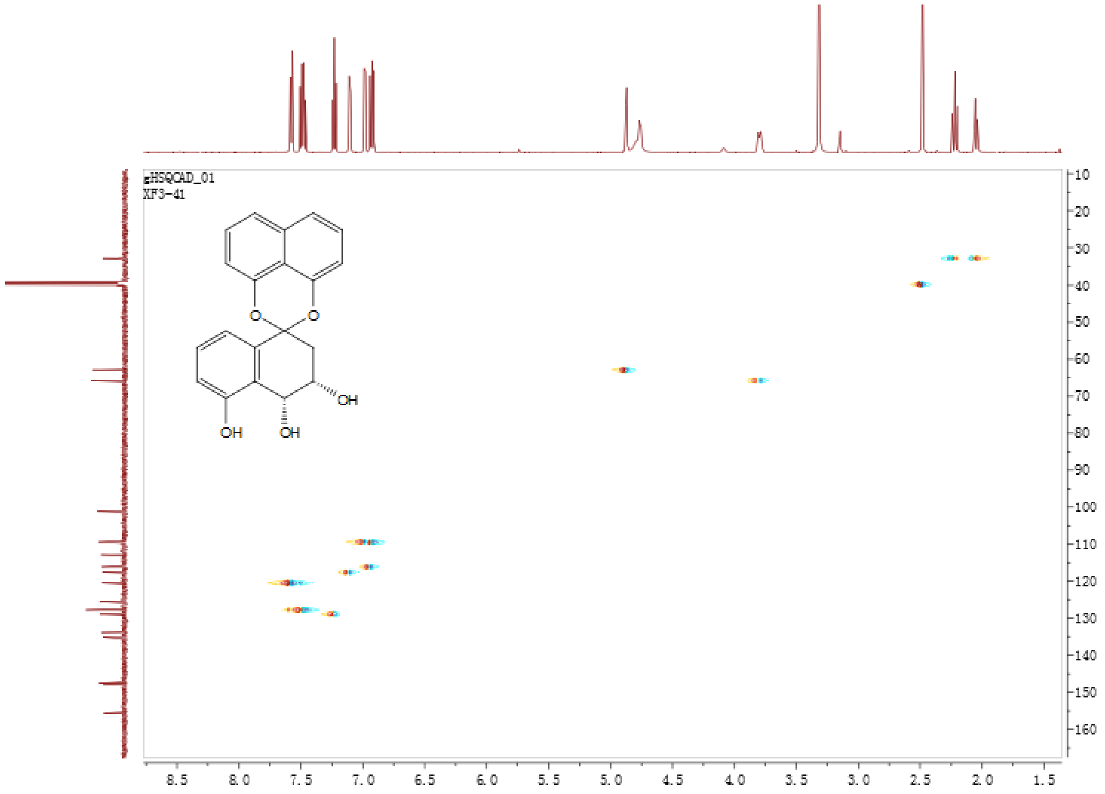

Figure S5. HSQC spectrum (600 MHz) of **1** in DMSO-d_6.

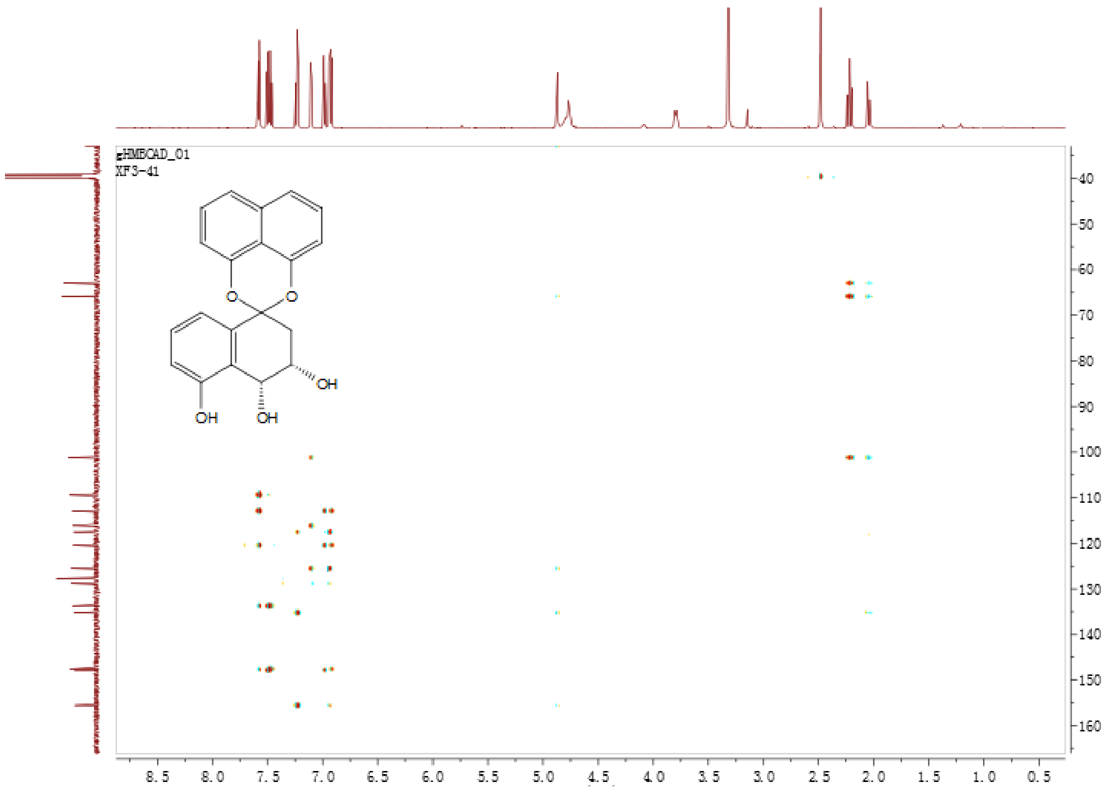

Figure S6. HMBC spectrum (600 MHz) of **1** in DMSO-d_6.

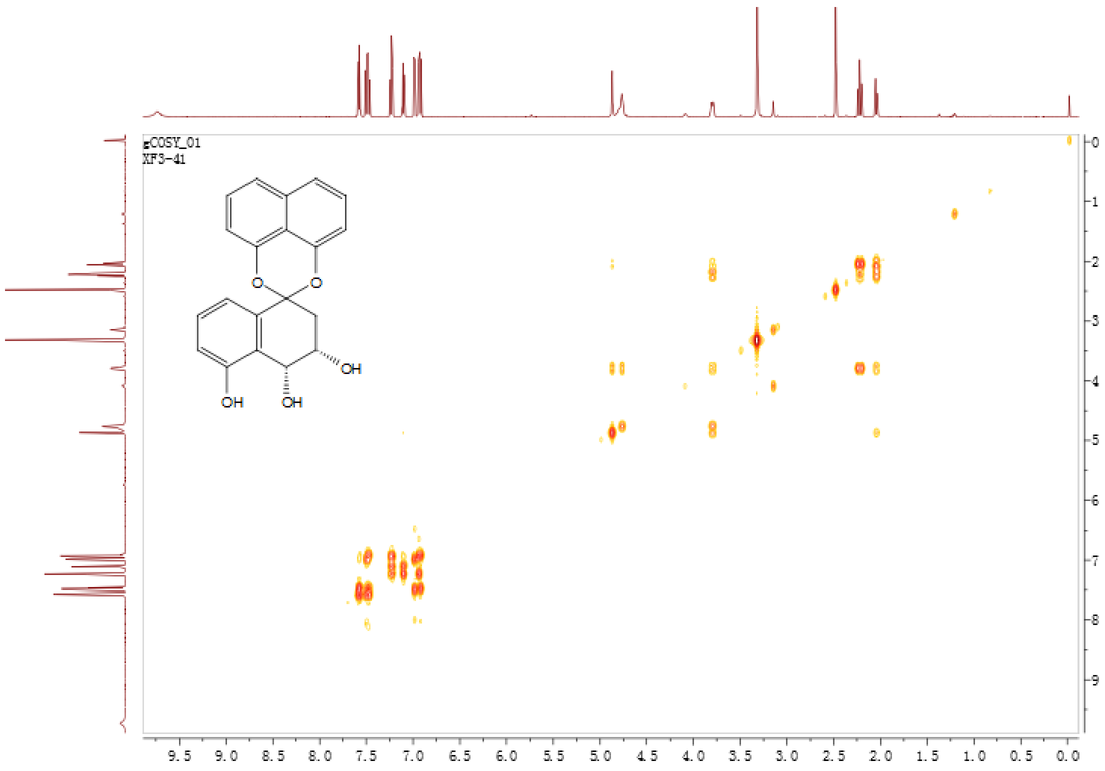

Figure S7. ^{1}H-^{1}H COSY spectrum (600 MHz) of **1** in DMSO-d_6.

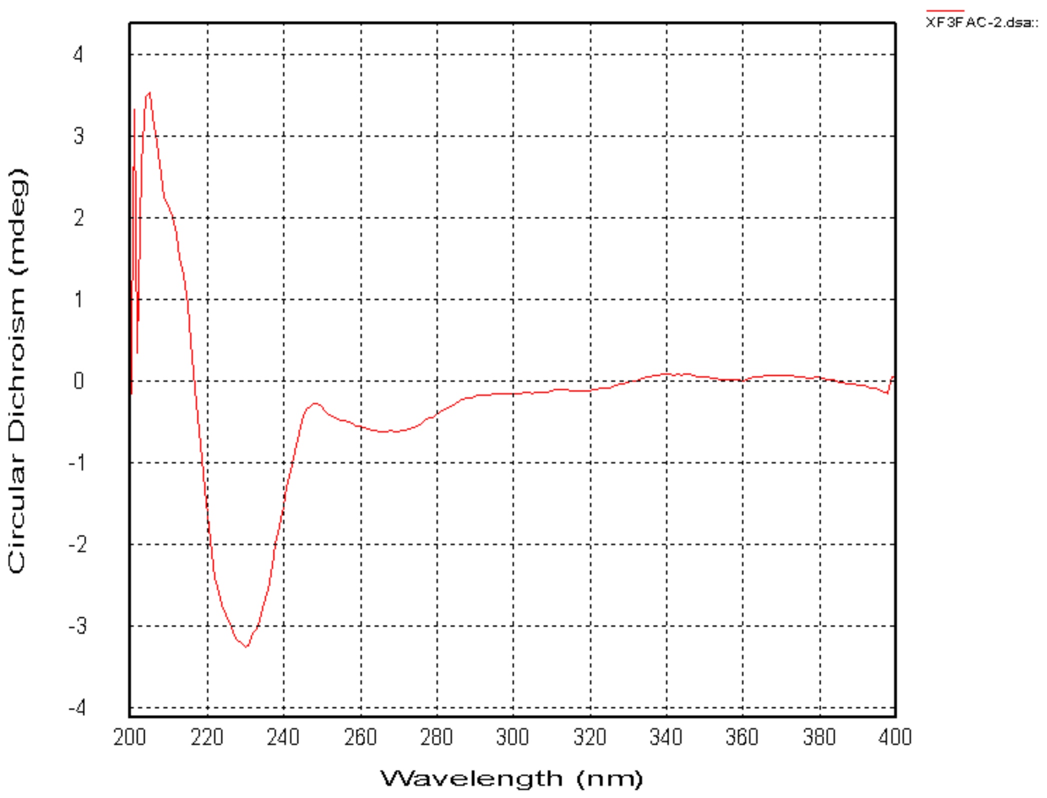

Figure S8. CD spectrum of **1**.

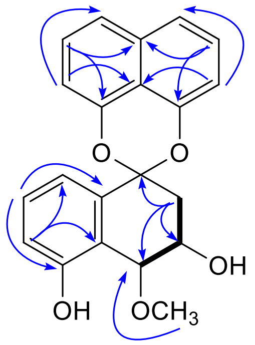

Figure S9. Key ^{1}H–^{1}H COSY (bold lines), HMBC (H→C) correlations for compound **2**.

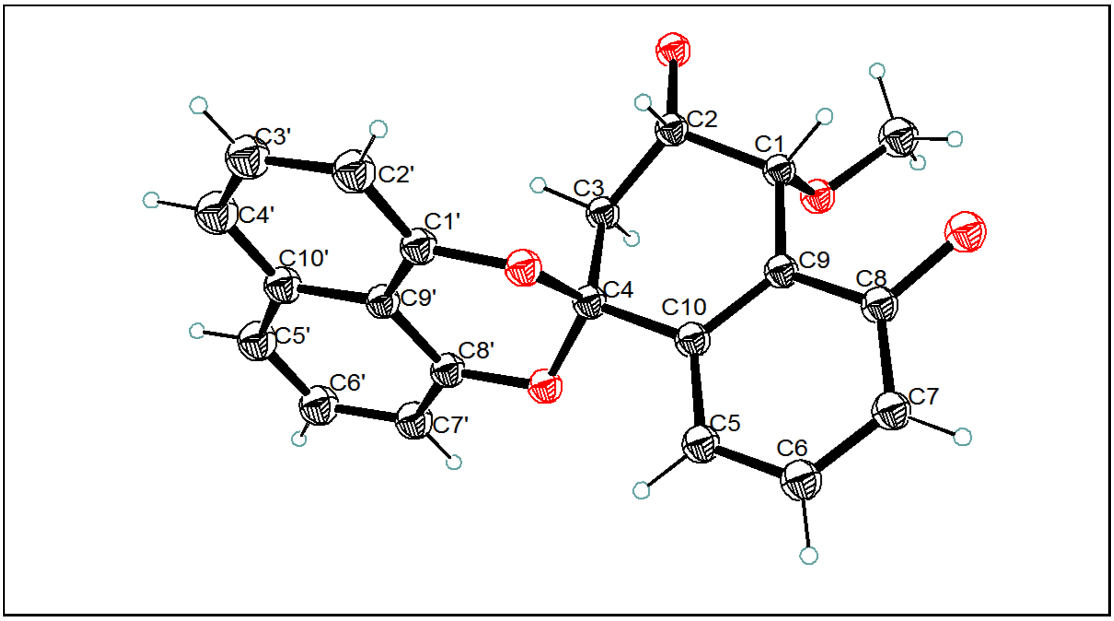

Figure S10. X-ray crystallographic structure of **2**.

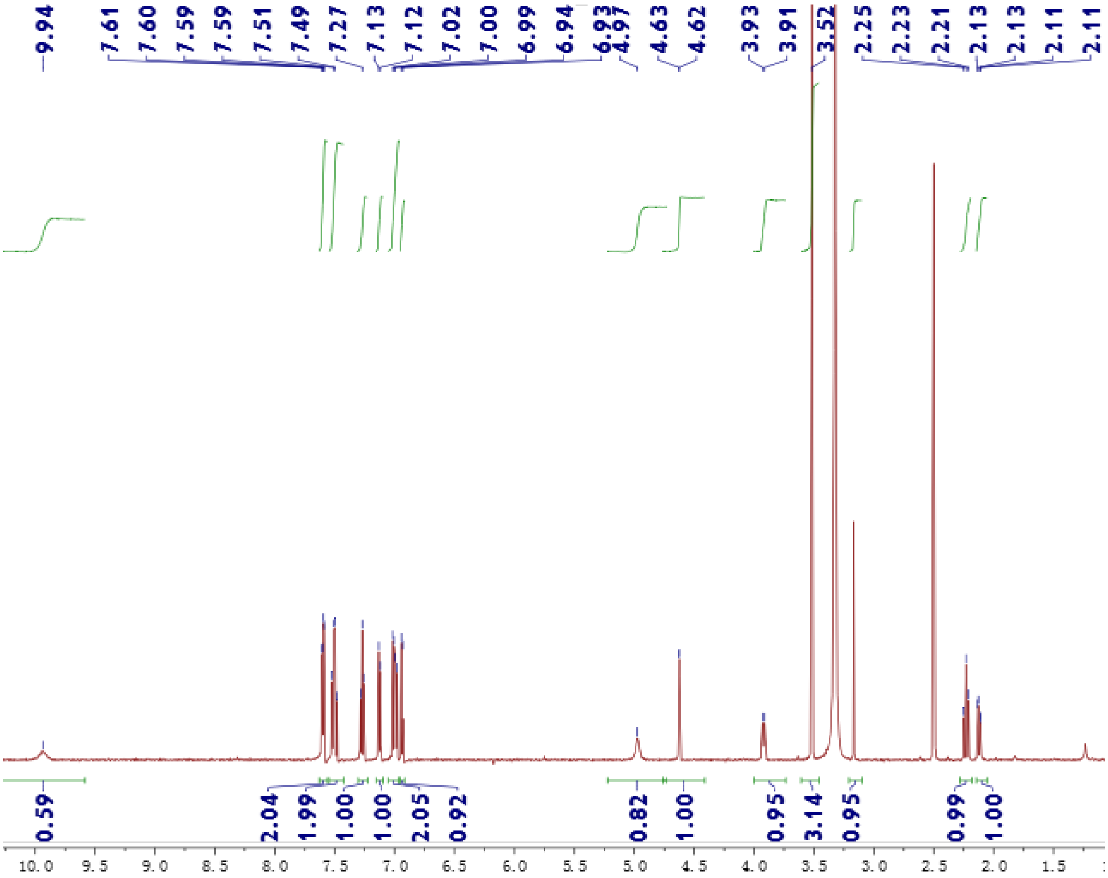

Figure S11. ^{1}H NMR spectrum (600 MHz) of **2** in DMSO-d_6.

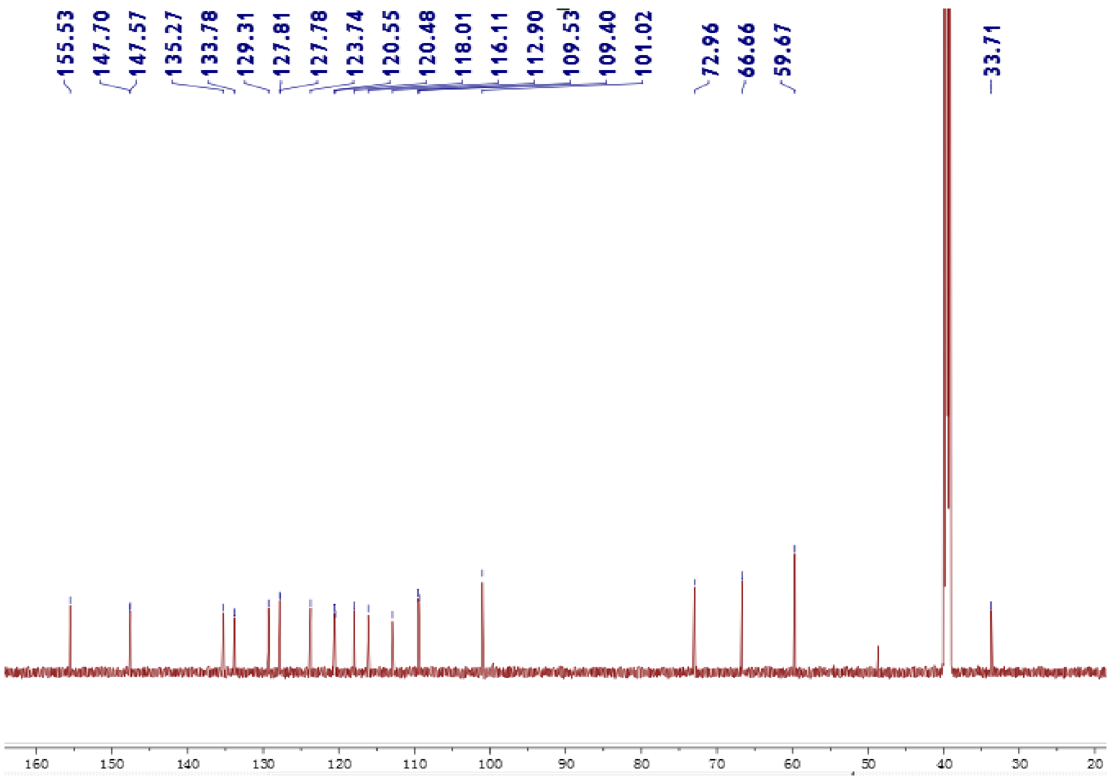

Figure S12. ^{13}C NMR spectrum (150 MHz) of **2** in DMSO-d_6.

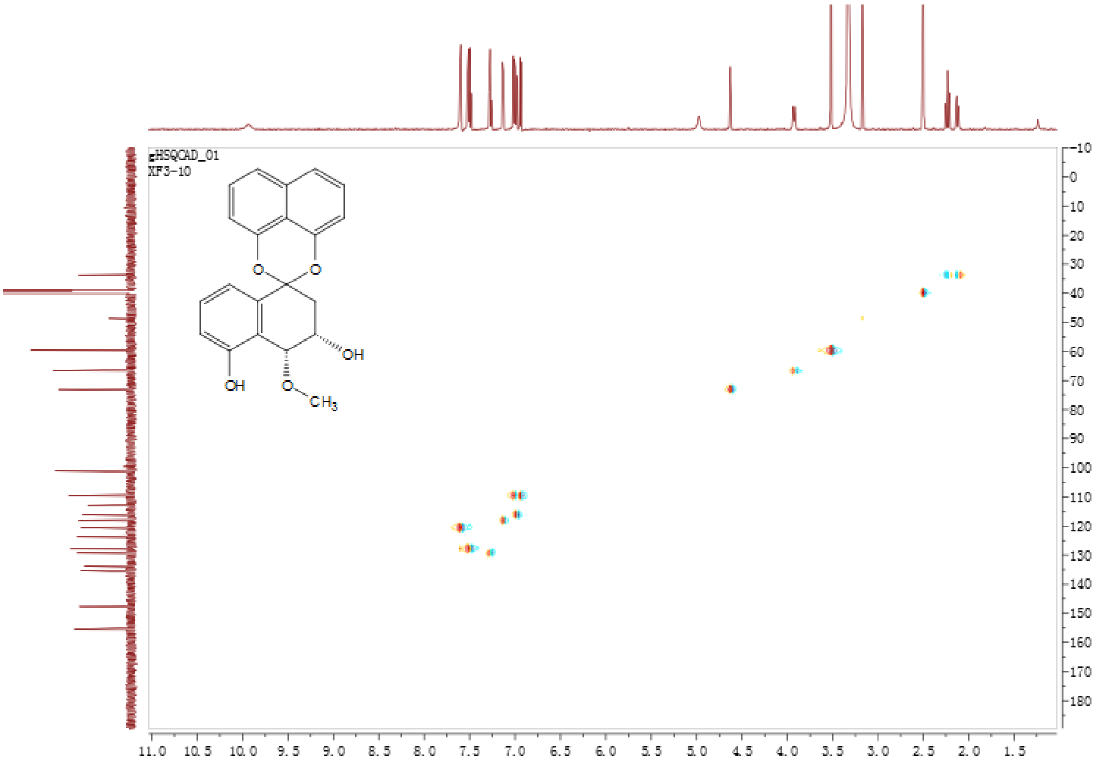

Figure S13. HSQC spectrum (600 MHz) of **2** in DMSO-d_6.

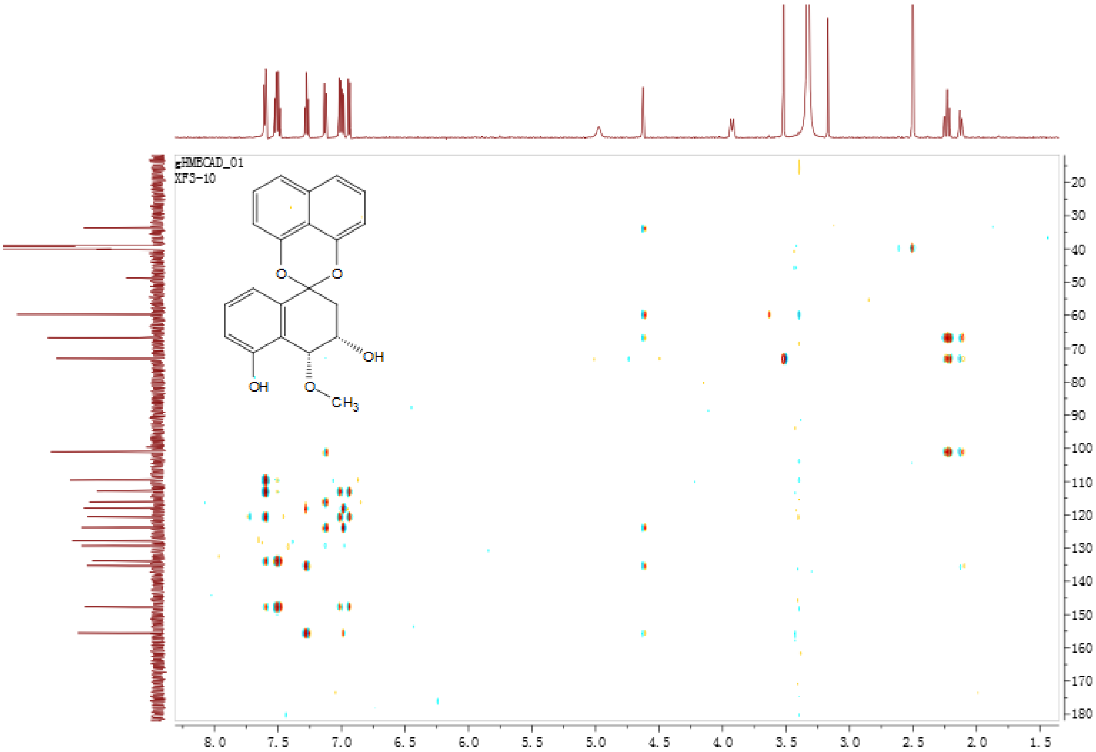

Figure S14. HMBC spectrum (600 MHz) of **2** in DMSO-d_6.

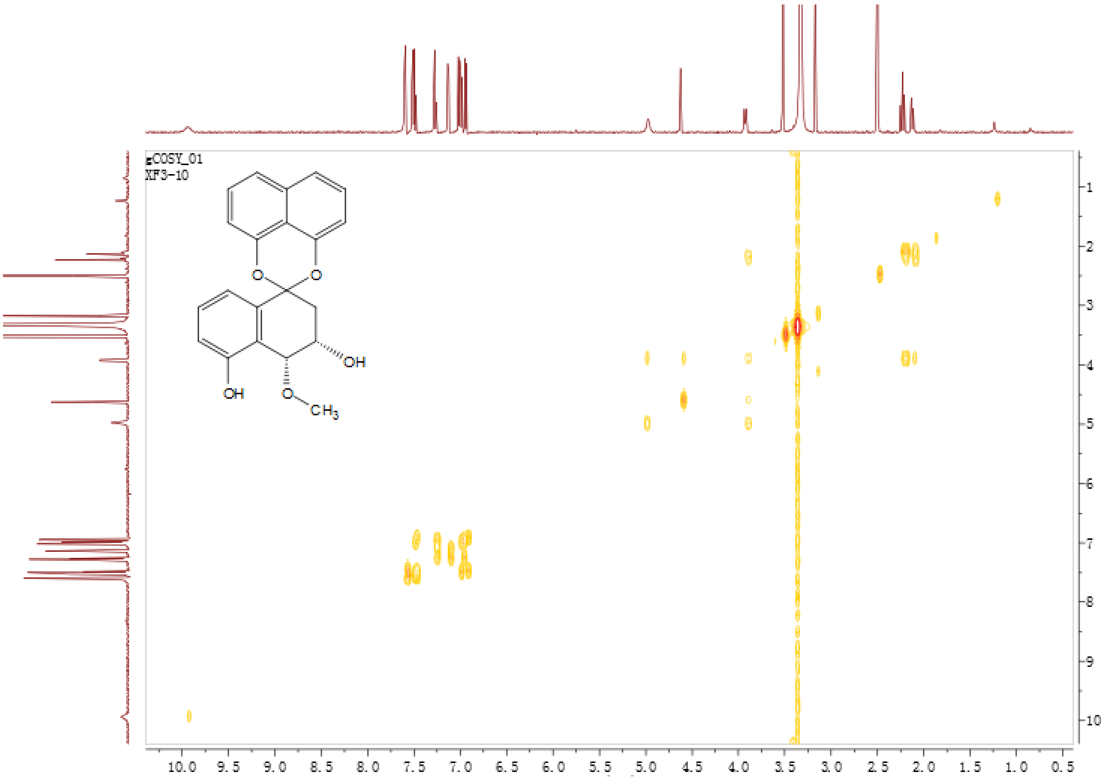

Figure S15. ^{1}H-^{1}H COSY spectrum (600 MHz) of **2** in DMSO-d_6.

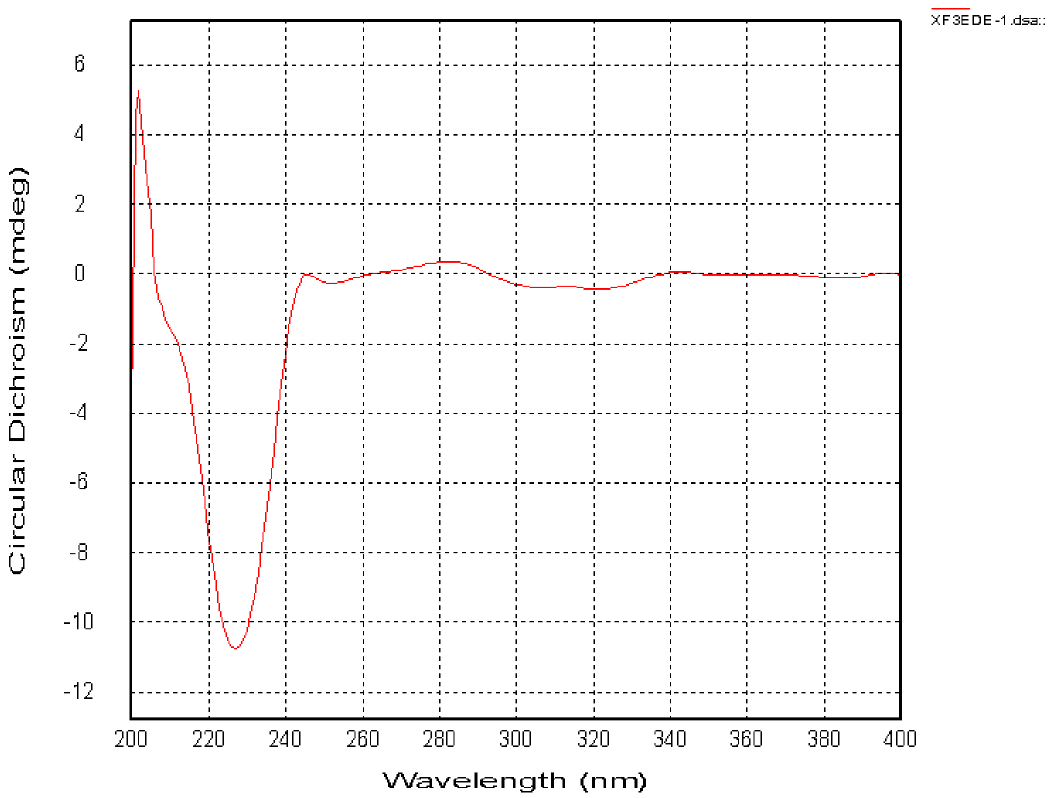

Figure S16. CD spectrum of **2**.

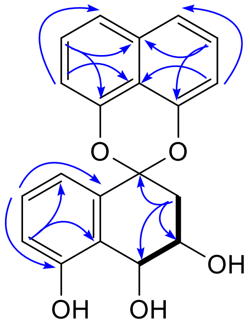

Figure S17. Key 1H−1H COSY (bold lines), HMBC (H→C) correlations for compound **3**.

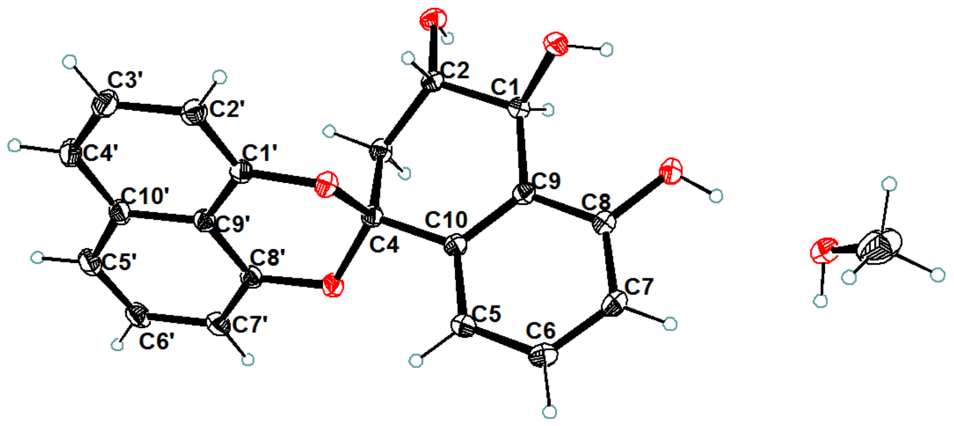

Figure S18. X-ray crystallographic structure of **3**.

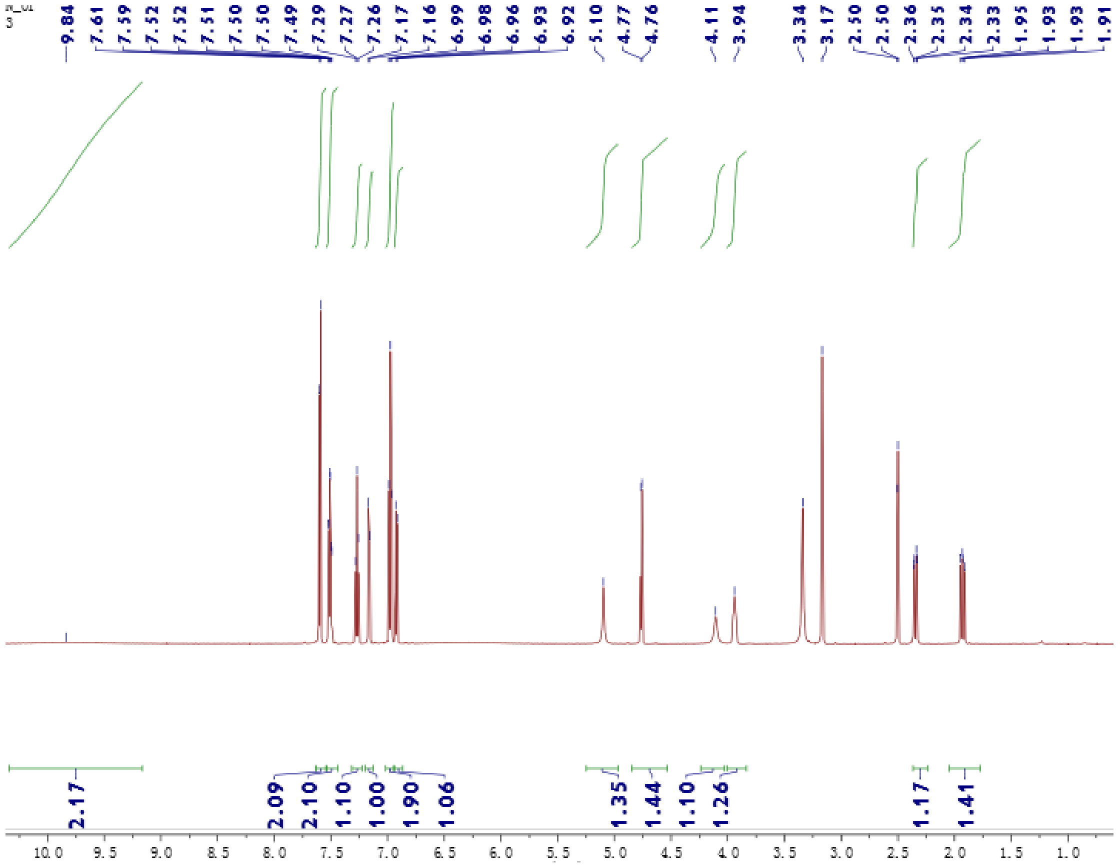

Figure S19. ^{1}H NMR spectrum (600 MHz) of **3** in DMSO-d_6.

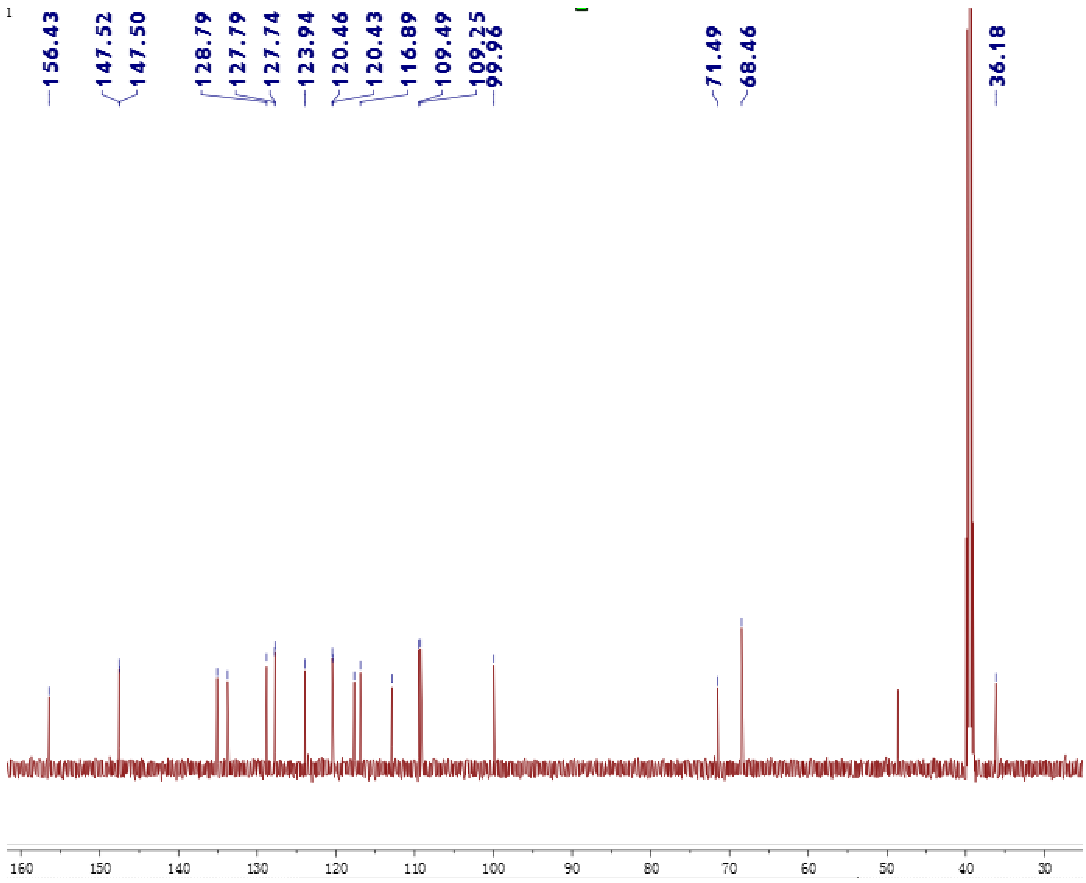

Figure S20. ^{13}C NMR spectrum (150 MHz) of **3** in DMSO-d_6.

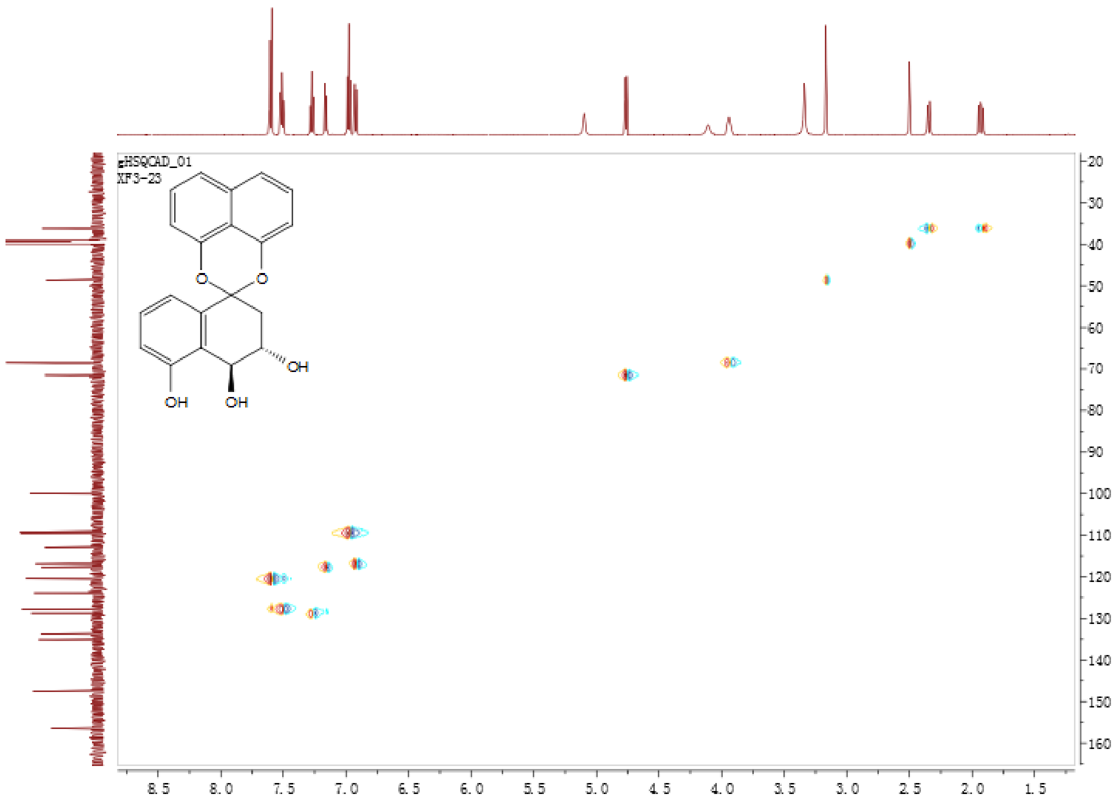

Figure S21. HSQC spectrum (600 MHz) of **3** in DMSO-d_6.

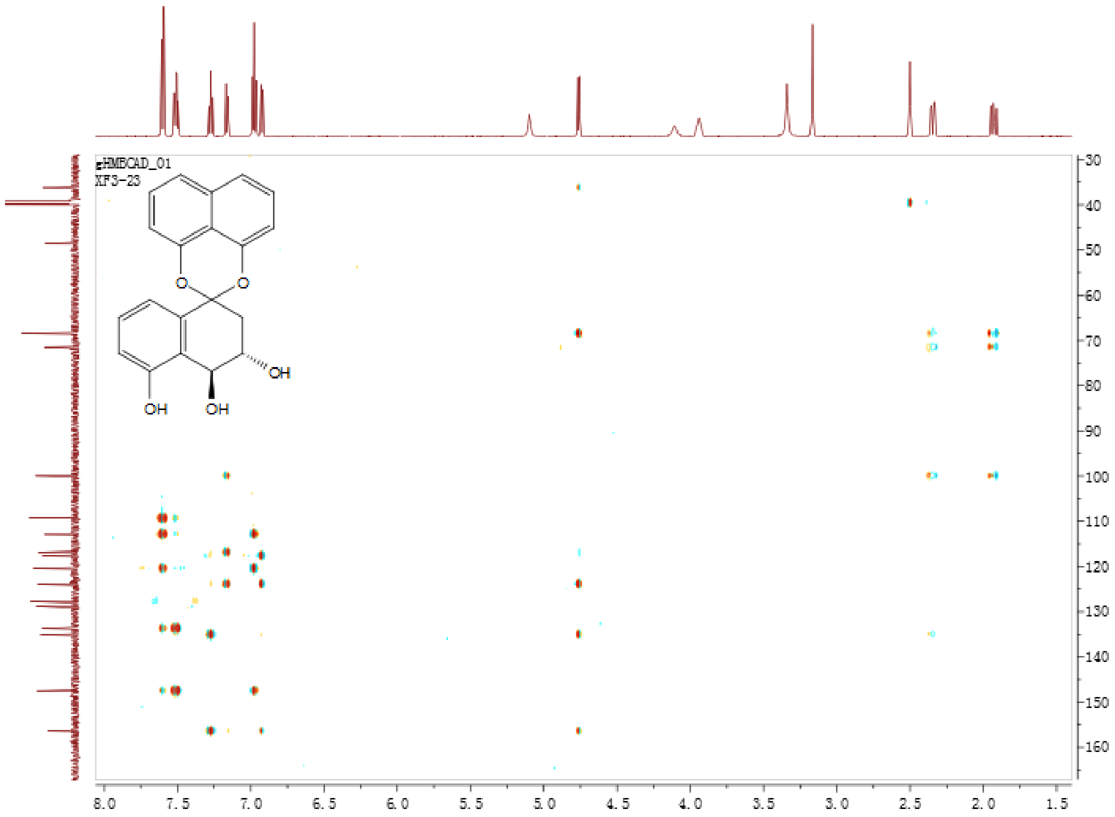

Figure S22. HMBC spectrum (600 MHz) of **3** in DMSO-d_6.

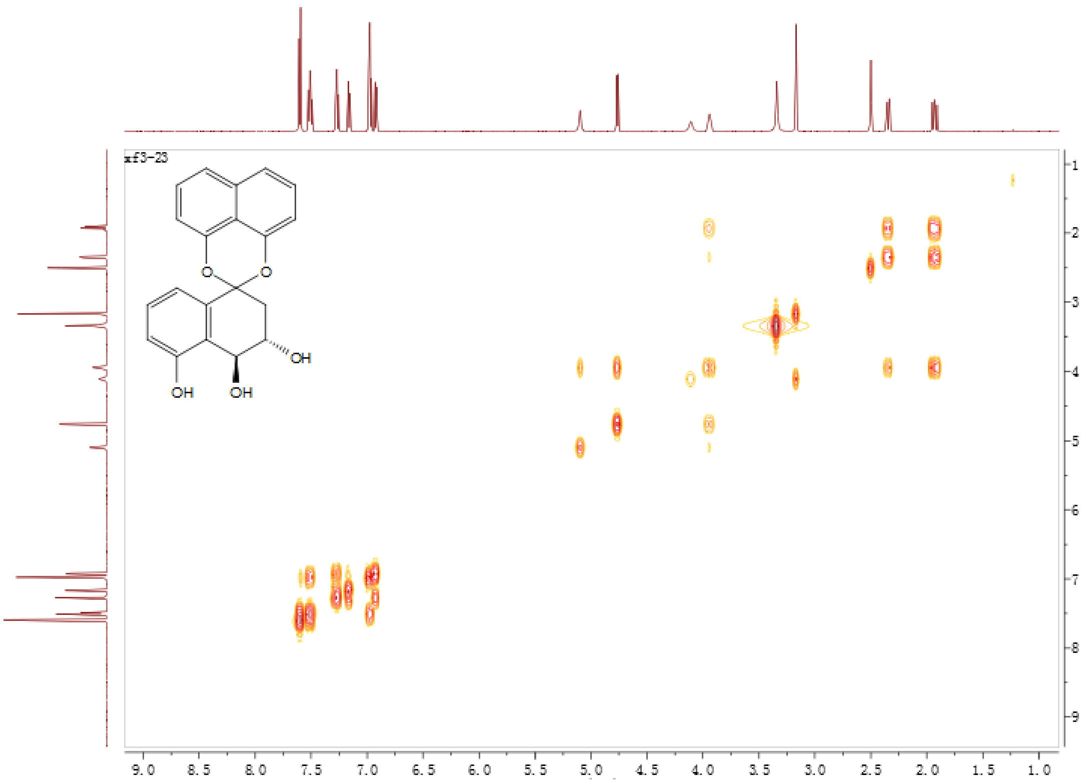

Figure S23. ^{1}H-^{1}H COSY spectrum (600 MHz) of **3** in DMSO-d_6.

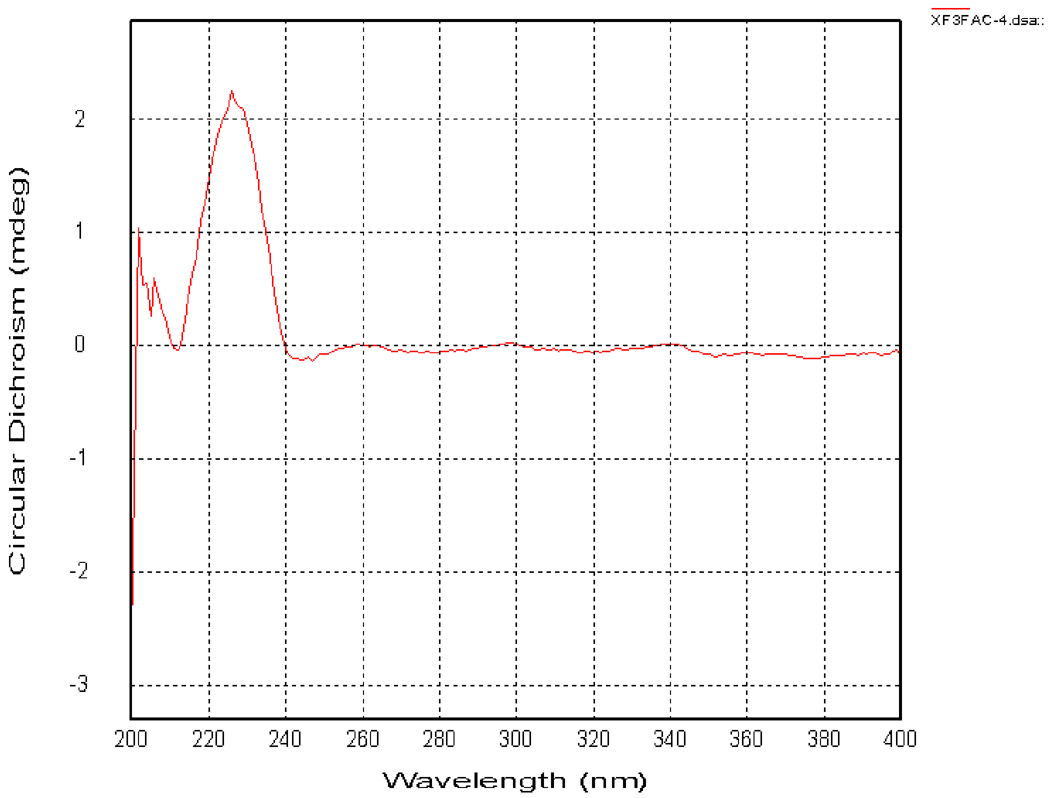

Figure S24. CD spectrum of **3**.

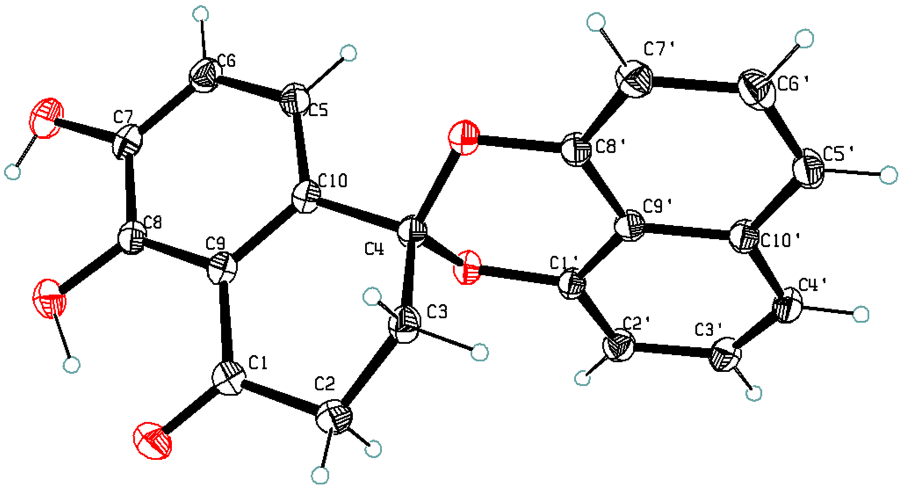

Figure S25. X-ray crystallographic structure of **4**.

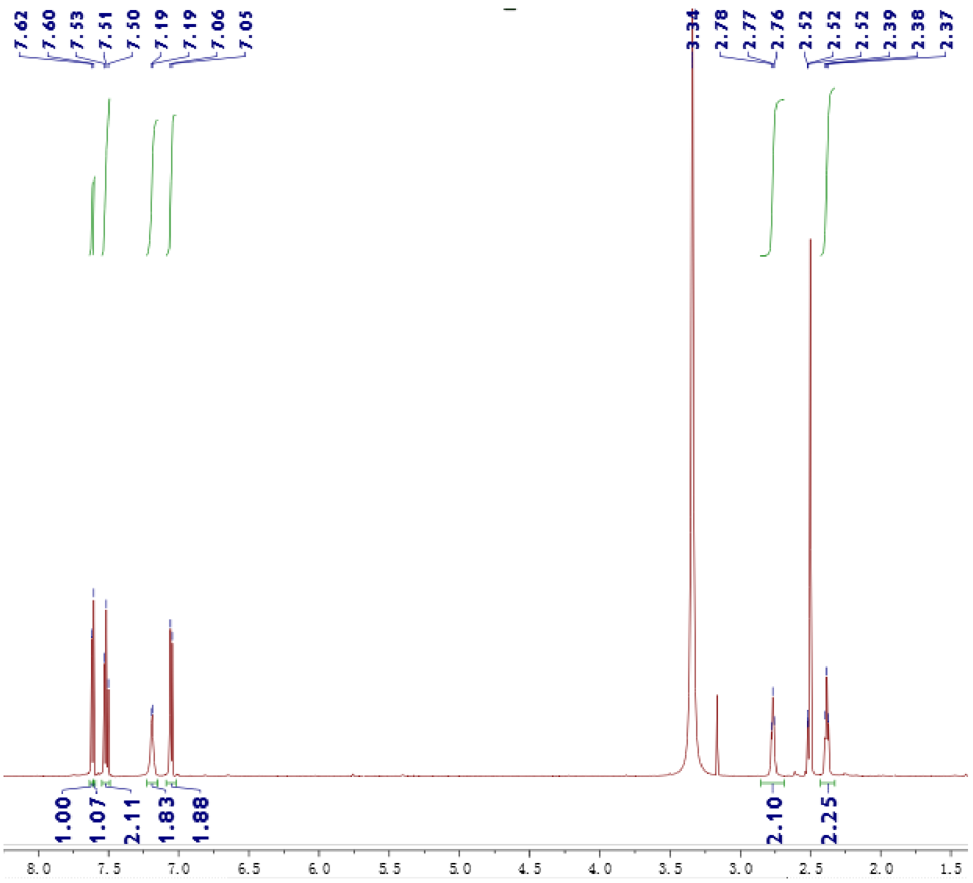

Figure S26. ^{1}H NMR spectrum (600 MHz) of **4** in DMSO-d_6.

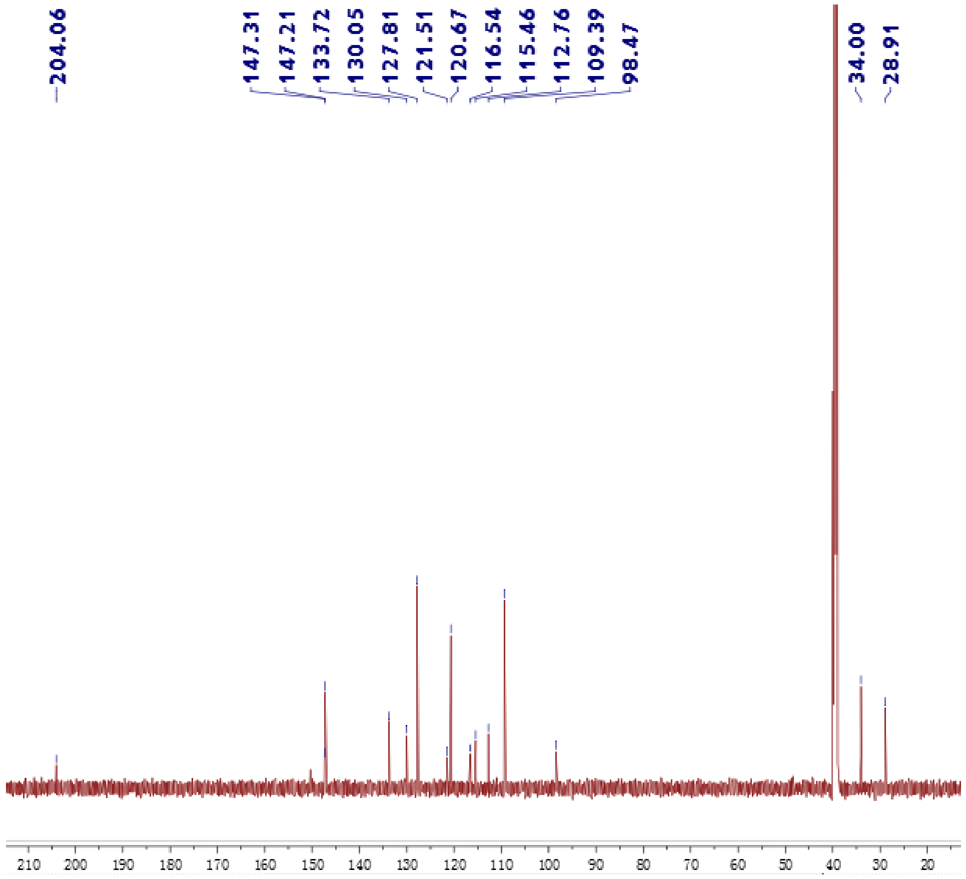

Figure S27. ^{13}C NMR spectrum (150 MHz) of **4** in DMSO-d_6.

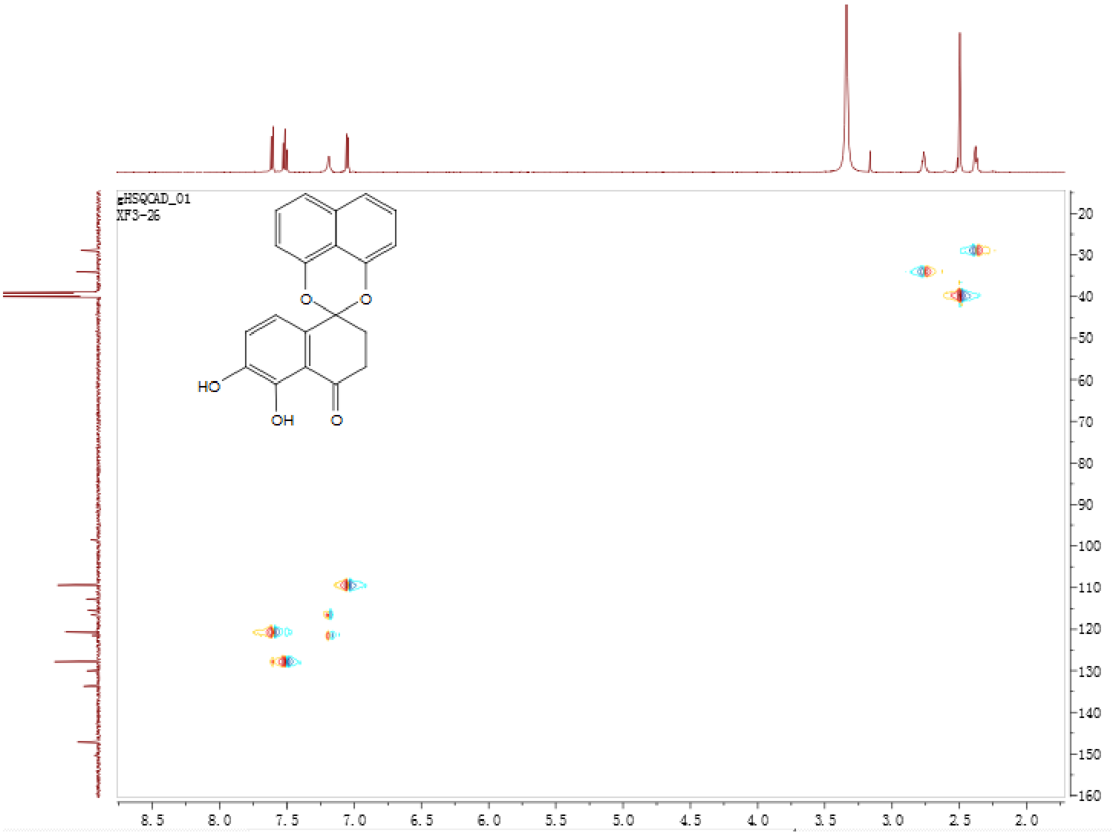

Figure S28. HSQC spectrum (600 MHz) of **4** in DMSO-d_6.

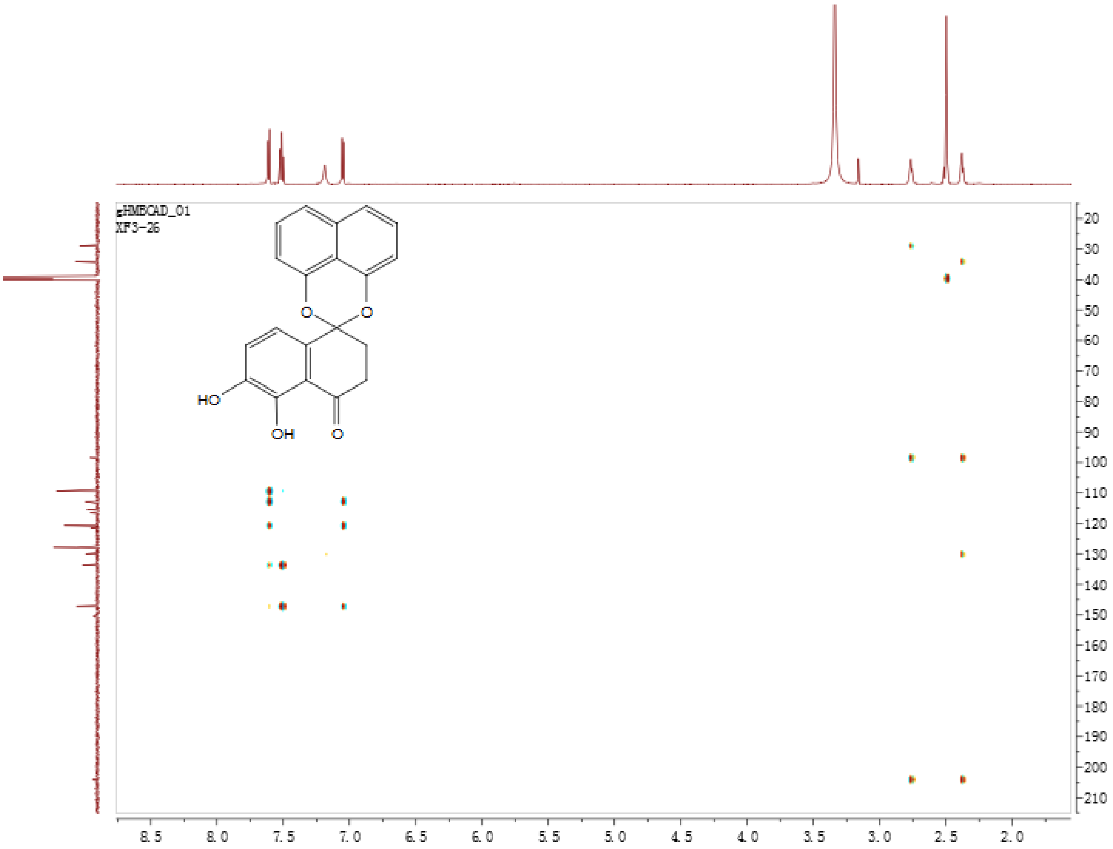

Figure S29. HMBC spectrum (600 MHz) of **4** in DMSO-d_6.

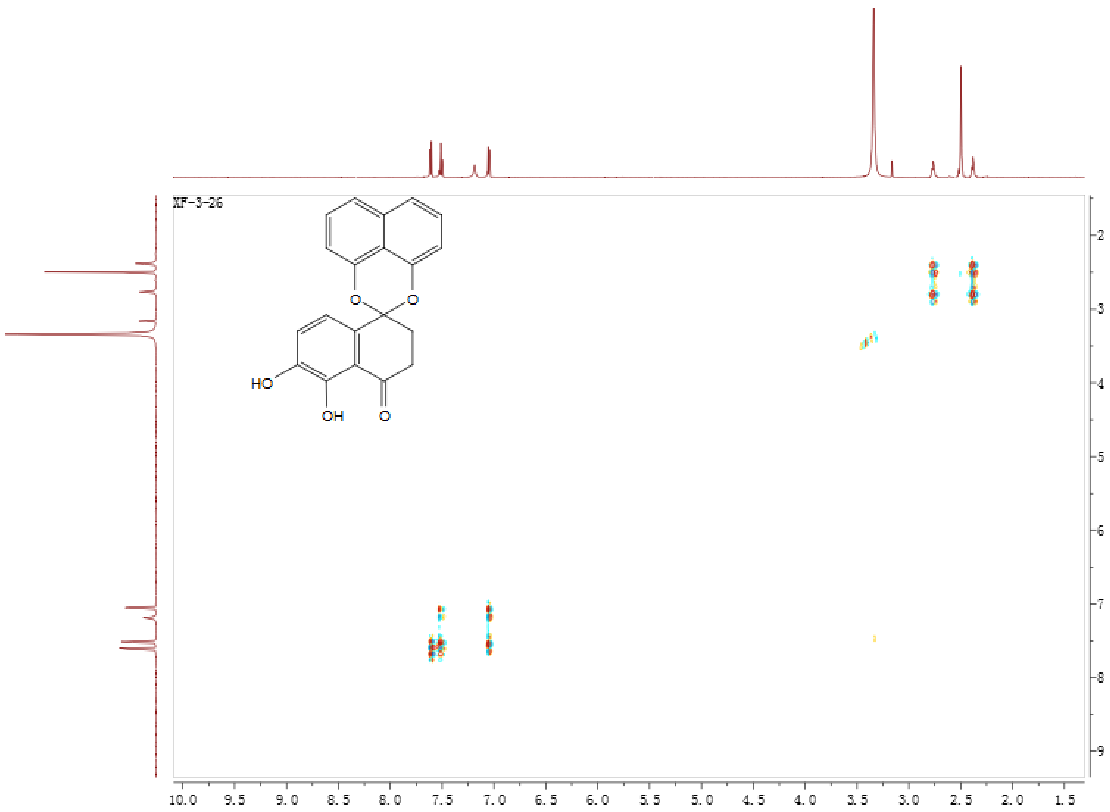

Figure S30. ^{1}H-^{1}H COSY spectrum (600 MHz) of **4** in DMSO-d_6.

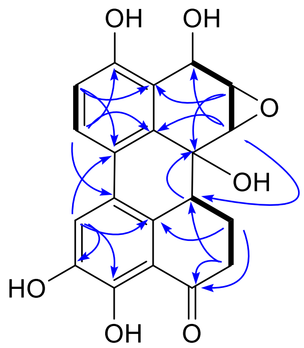

Figure S31. Key ^{1}H–^{1}H COSY (bold lines), HMBC (H→C) correlations for compound **7**.

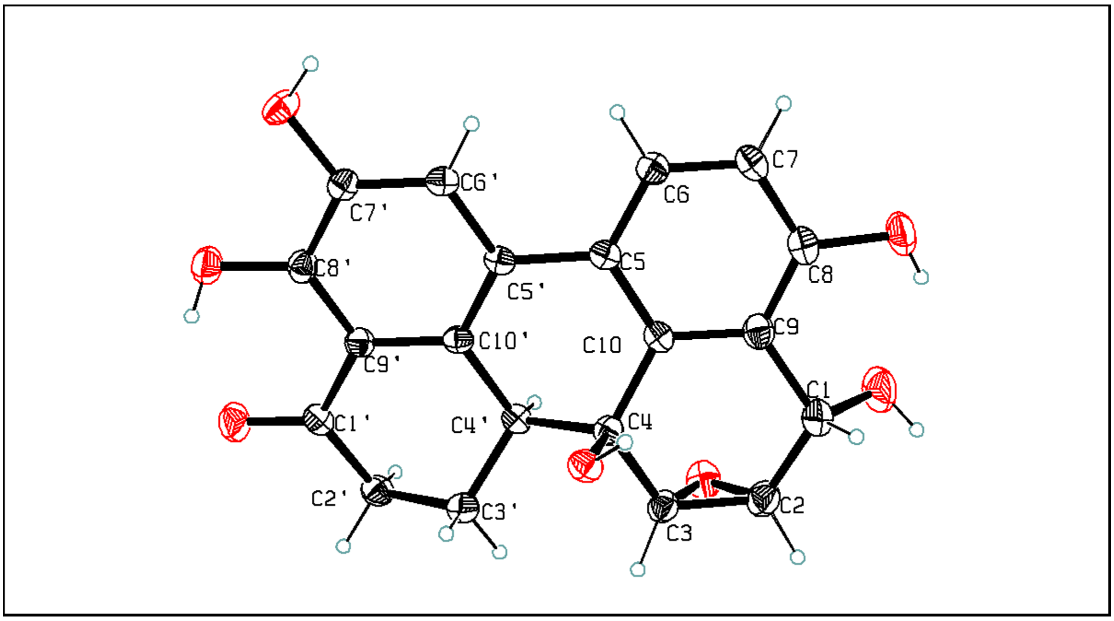

Figure S32. X-ray crystallographic structure of **7**.

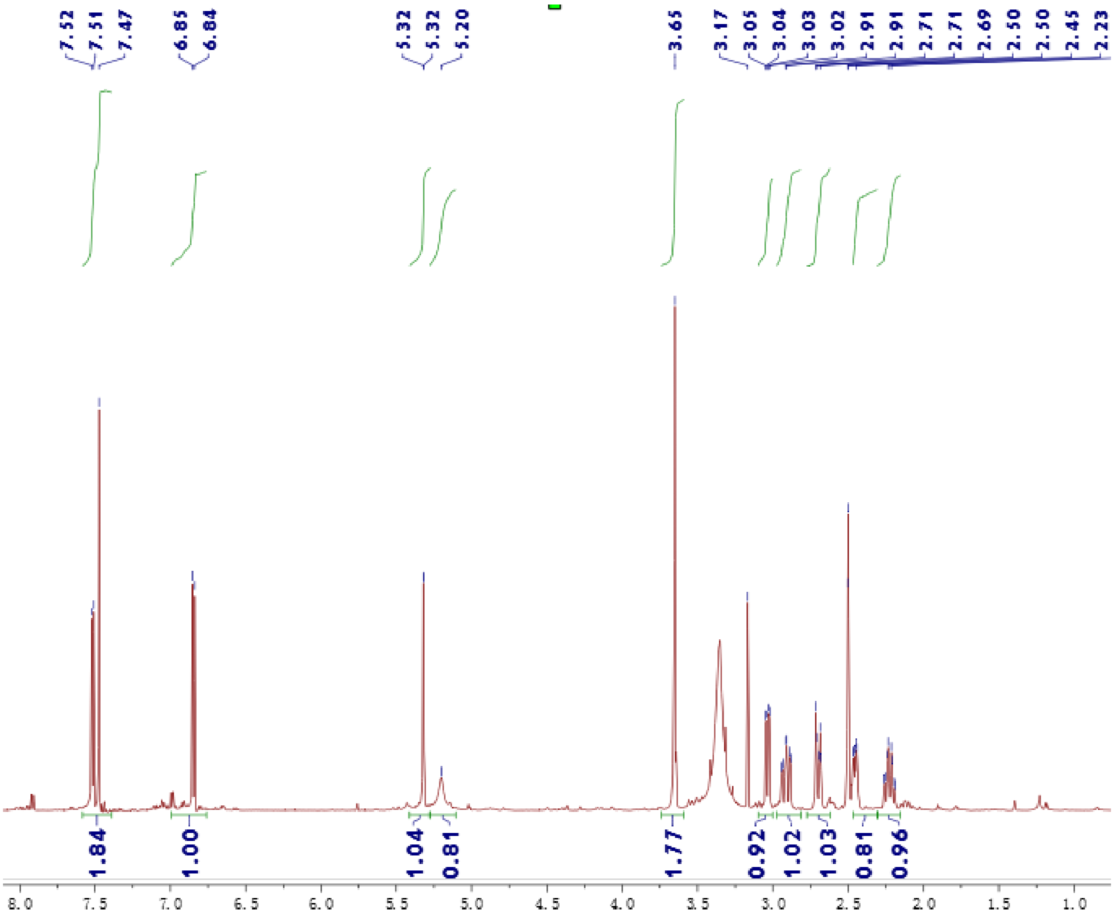

Figure S33. ^{1}H NMR spectrum (600 MHz) of **7** in DMSO-d_6.

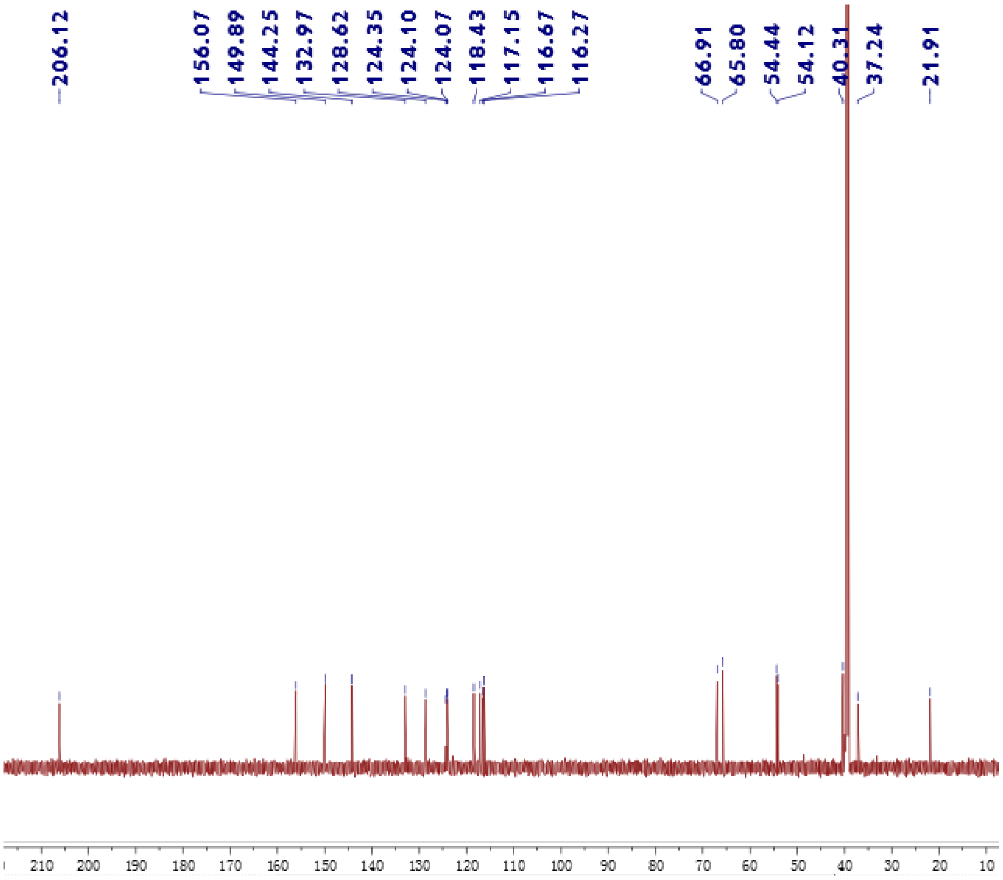

Figure S34. ^{13}C NMR spectrum (150 MHz) of **7** in DMSO-d_6.

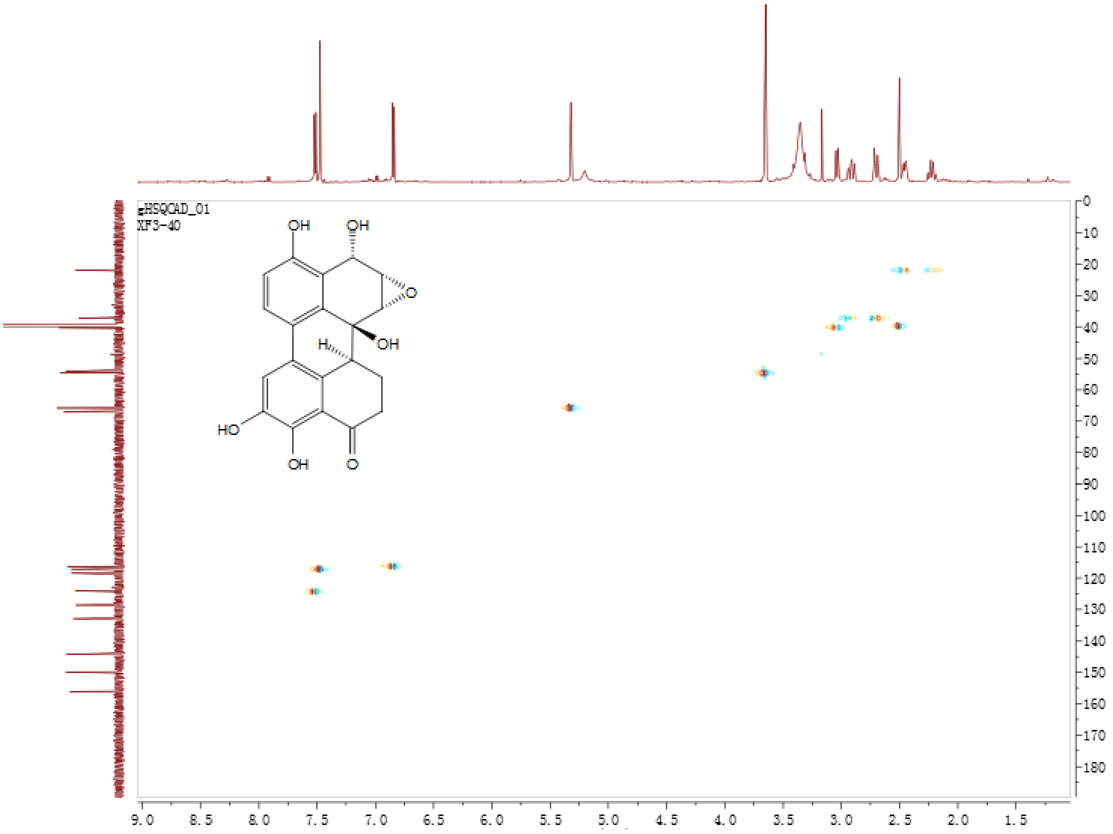

Figure S35. HSQC spectrum (600 MHz) of **7** in DMSO-d_6.

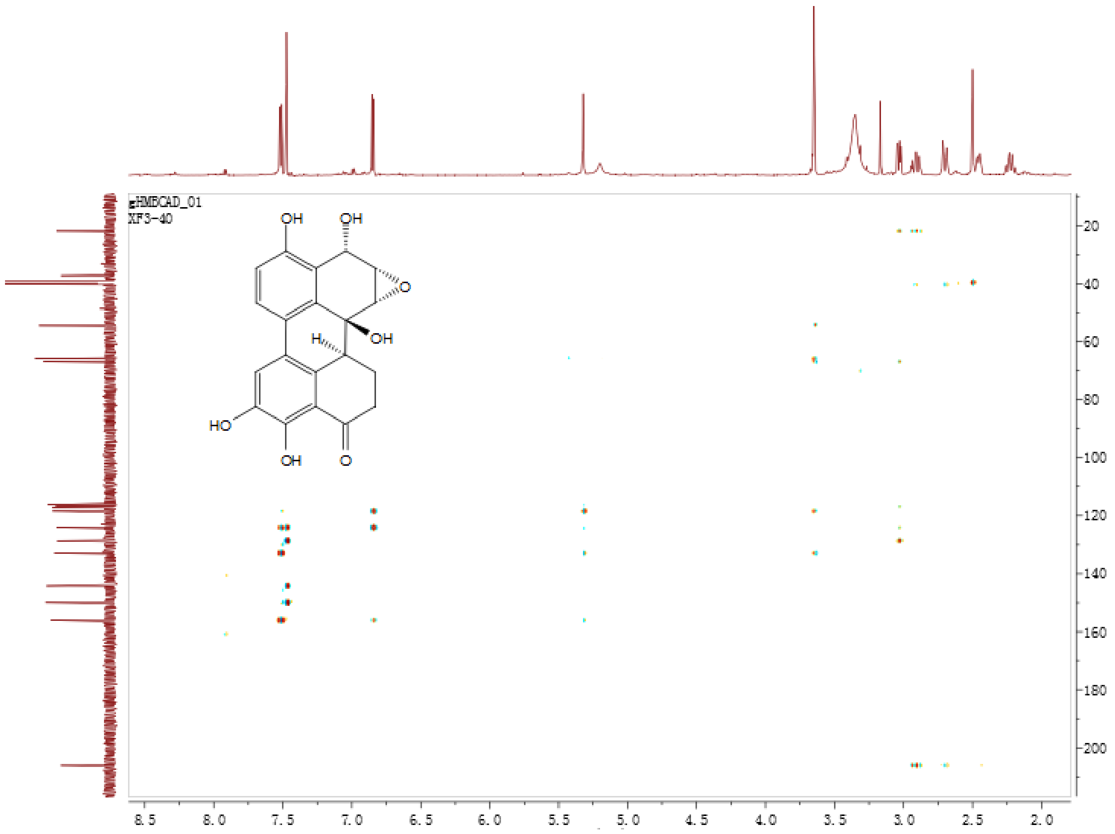

Figure S36. HMBC spectrum (600 MHz) of **7** in DMSO-d_6.

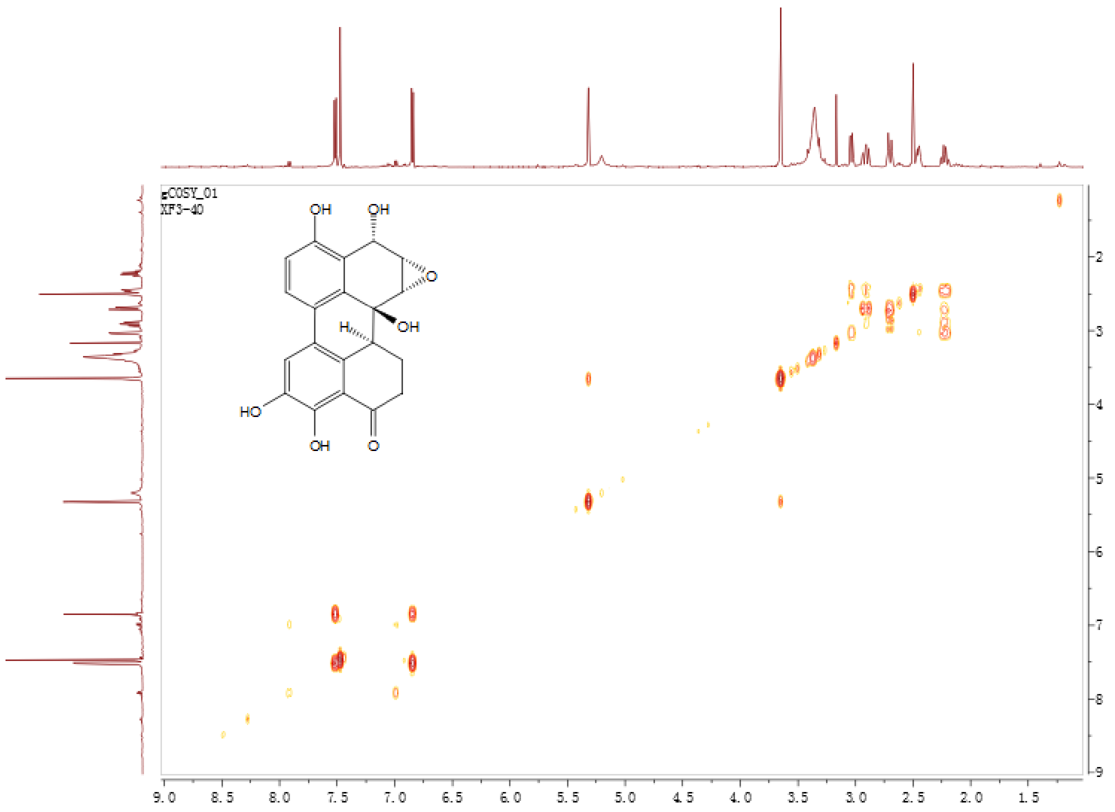

Figure S37. ^{1}H-^{1}H COSY spectrum (600 MHz) of **7** in DMSO-d_6.

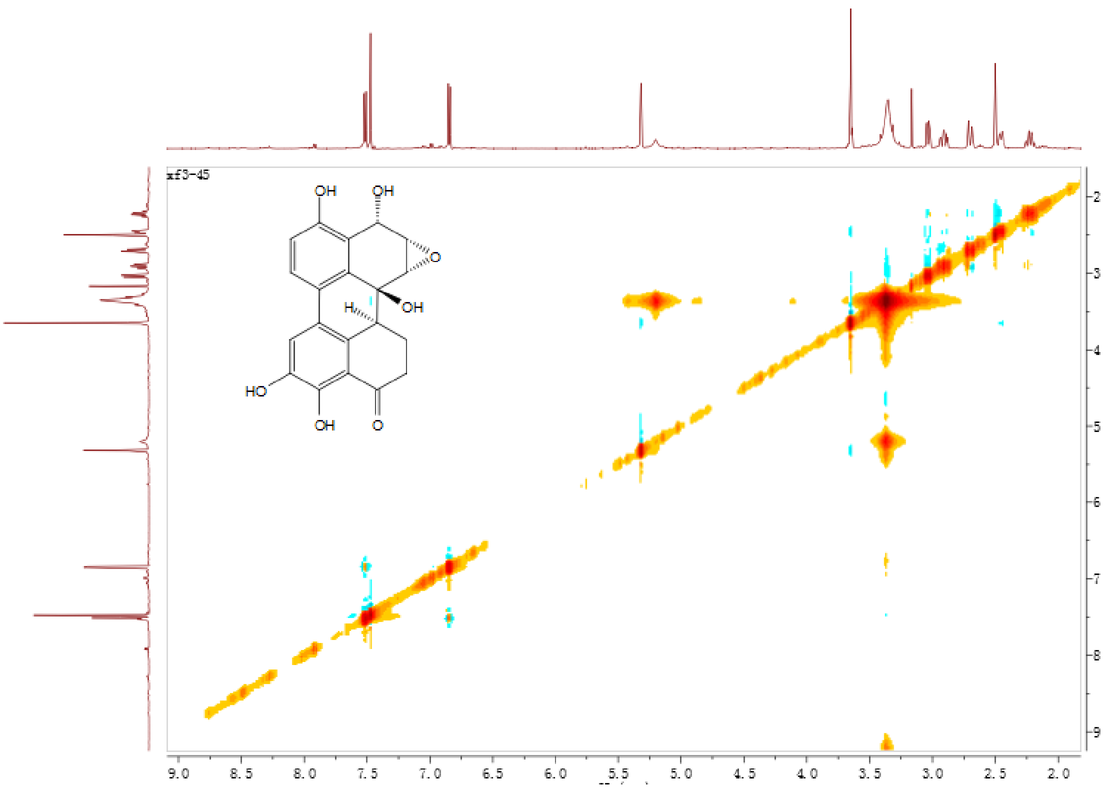

Figure S38. NOESY spectrum (600 MHz) of **7** in DMSO-d_6.

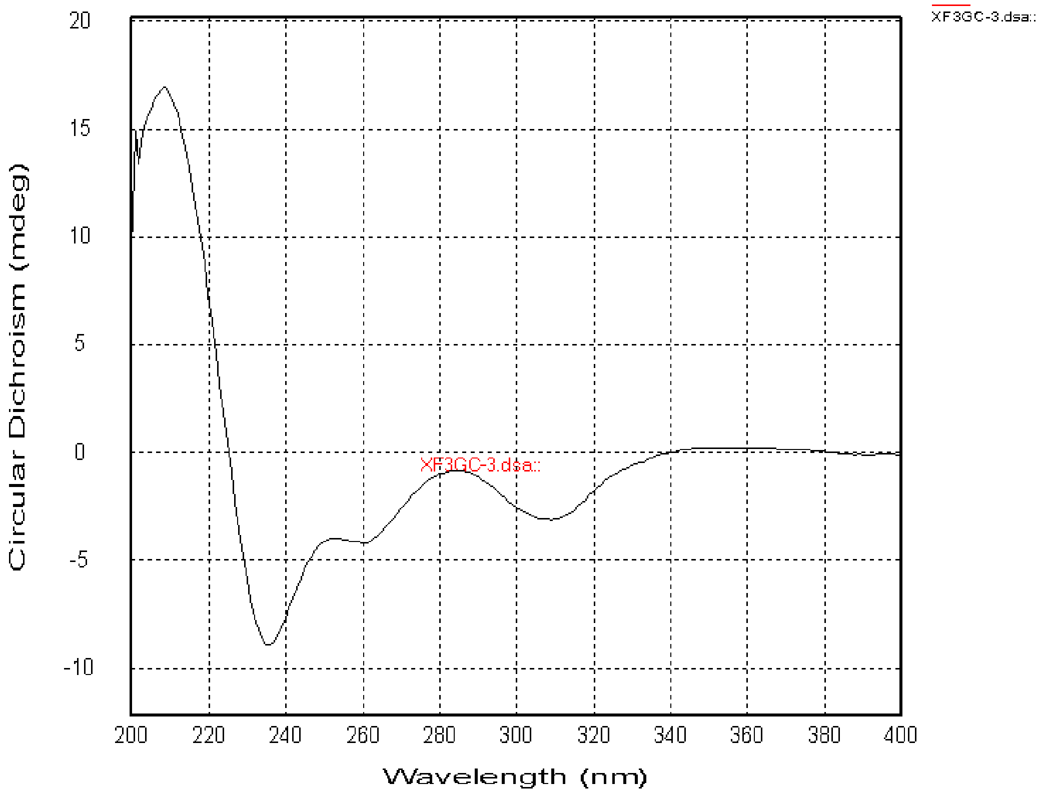

Figure S39. CD spectrum of **7**.

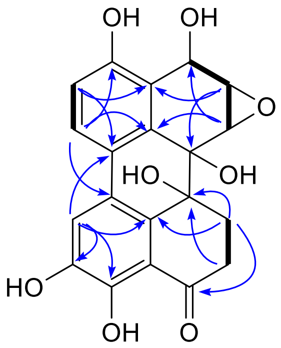

Figure S40. Key ^{1}H–^{1}H COSY (bold lines), HMBC (H→C) correlations for compound **8**.

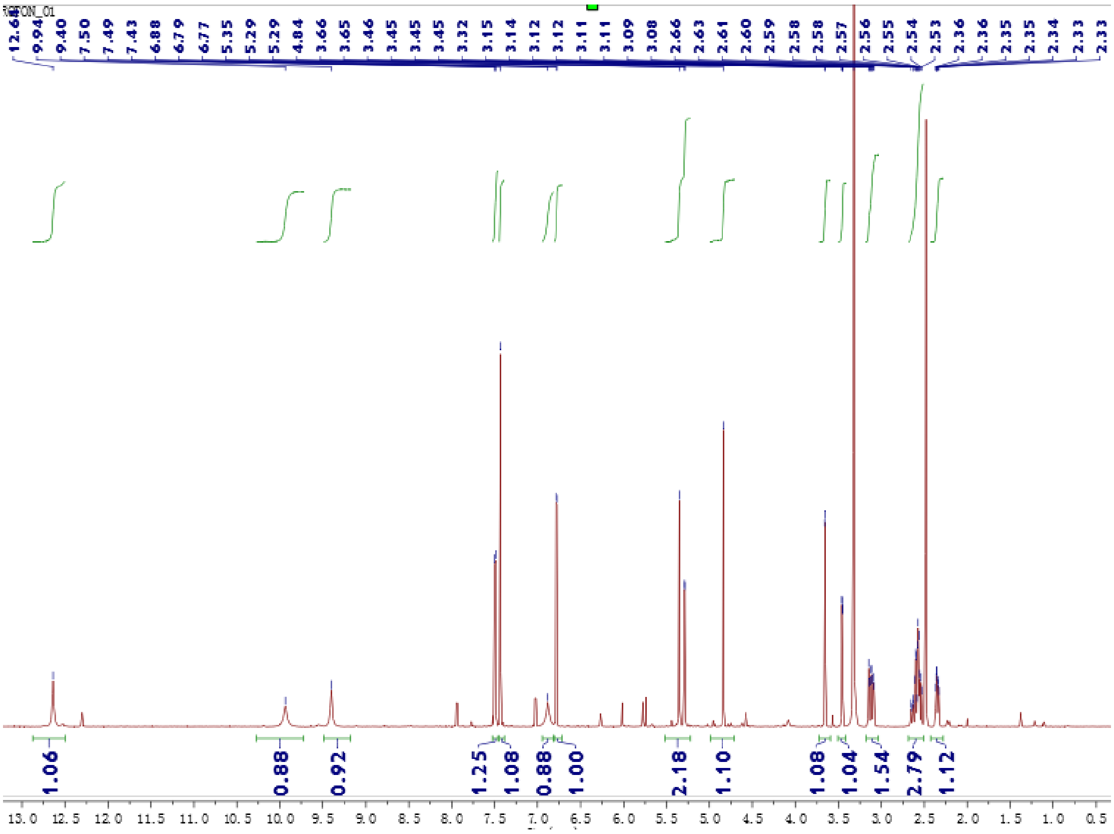

Figure S41. ^{1}H NMR spectrum (600 MHz) of **8** in DMSO-d_6.

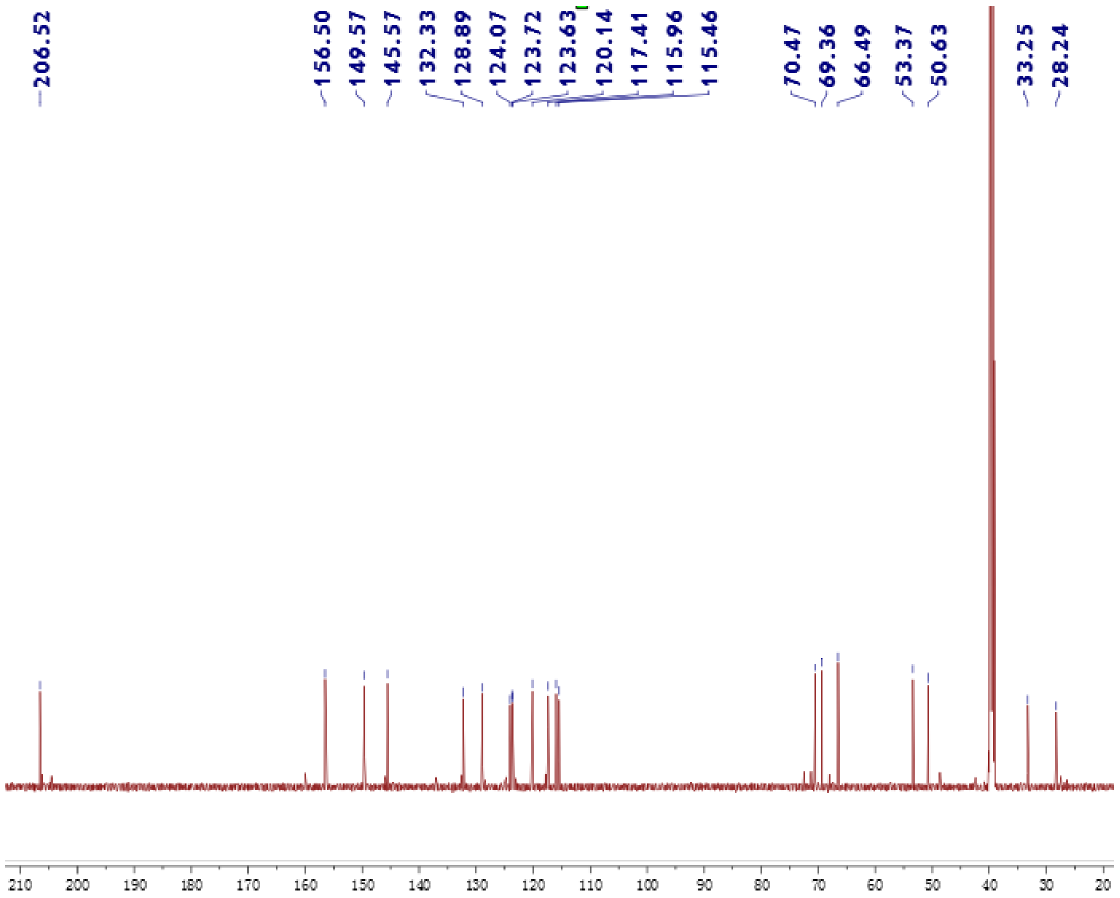

Figure S42. ^{13}C NMR spectrum (150 MHz) of **8** in DMSO-d_6.

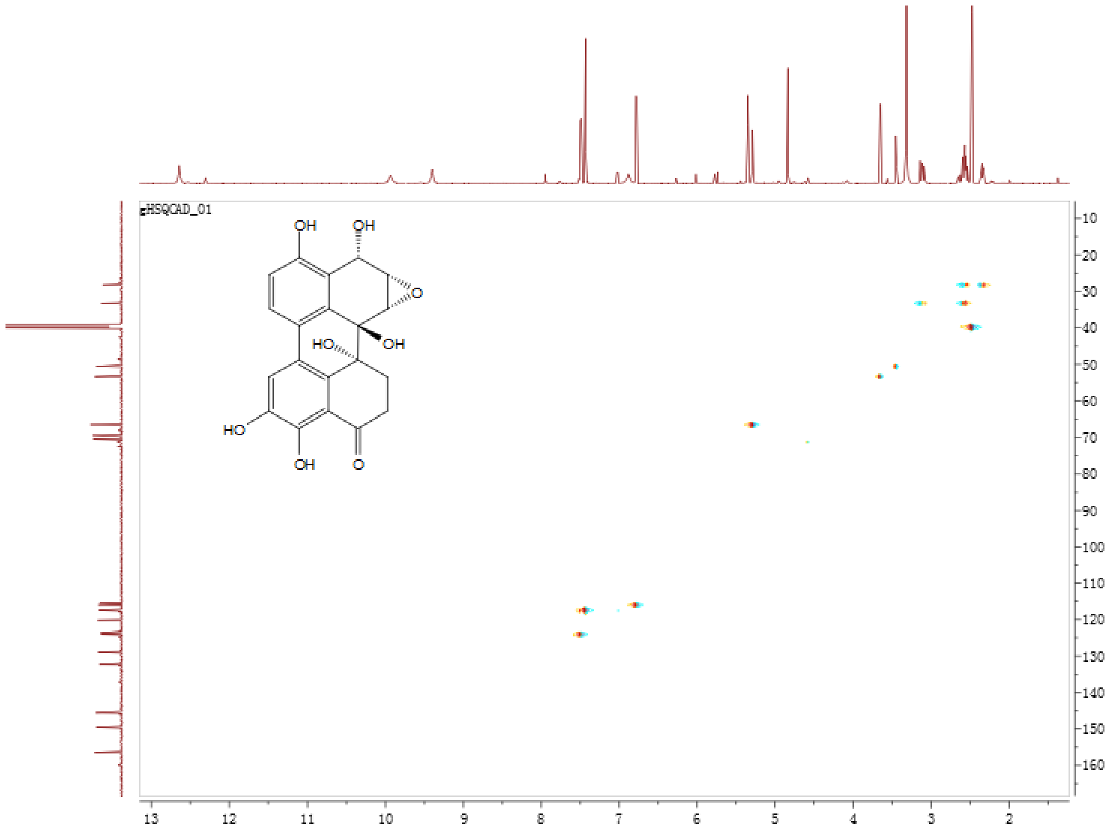

Figure S43. HSQC spectrum (600 MHz) of **8** in DMSO-d_6.

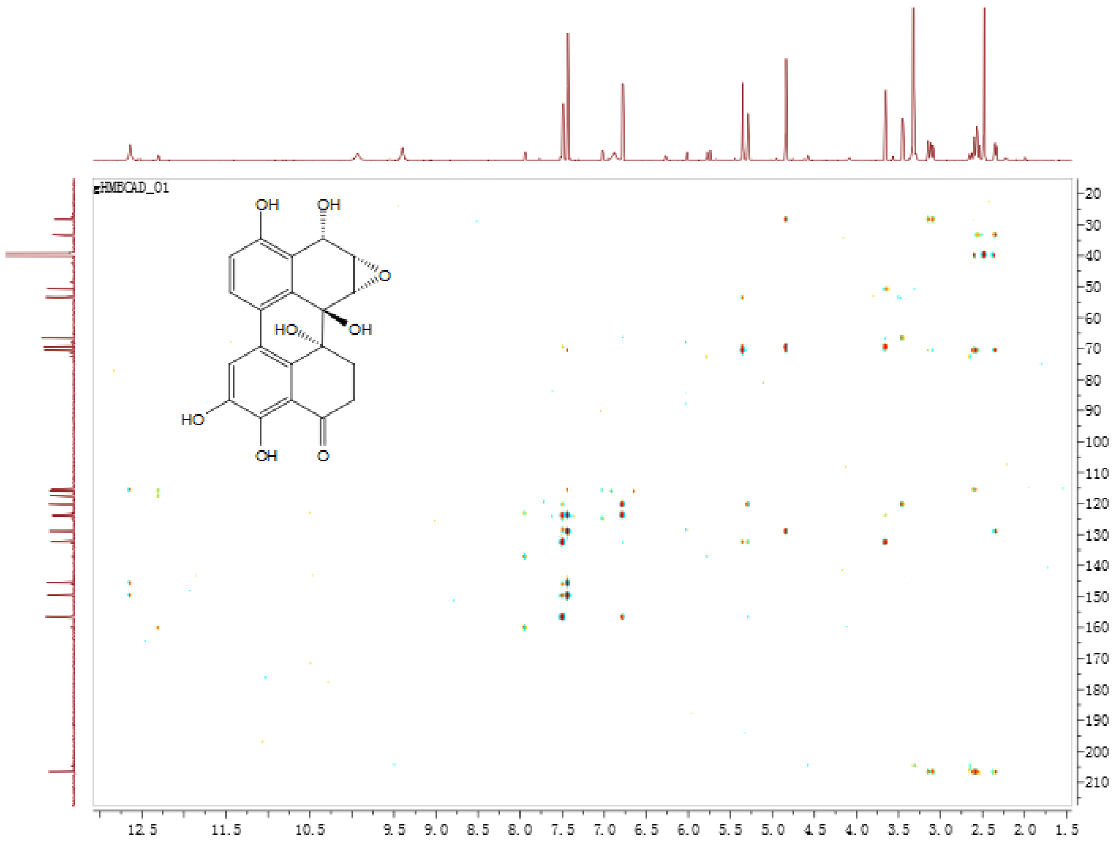

Figure S44. HMBC spectrum (600 MHz) of **8** in DMSO-d_6.

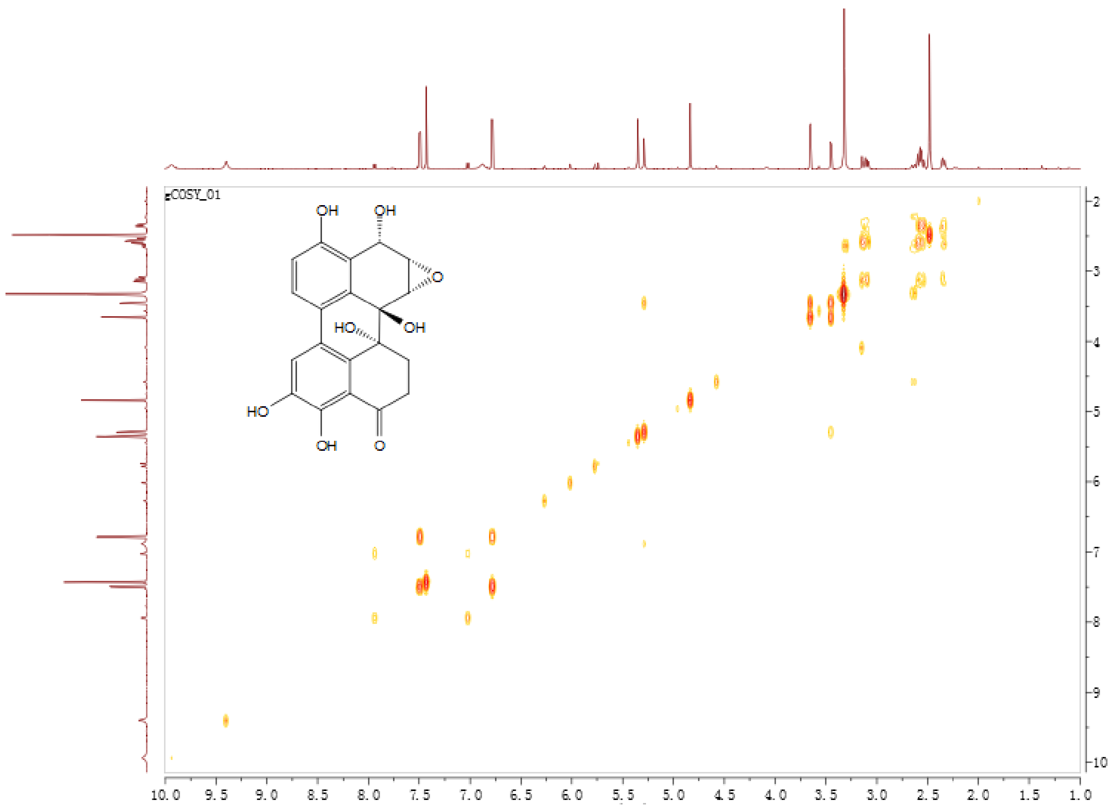

Figure S45. ^{1}H-^{1}H COSY spectrum (600 MHz) of **8** in DMSO-d_6.

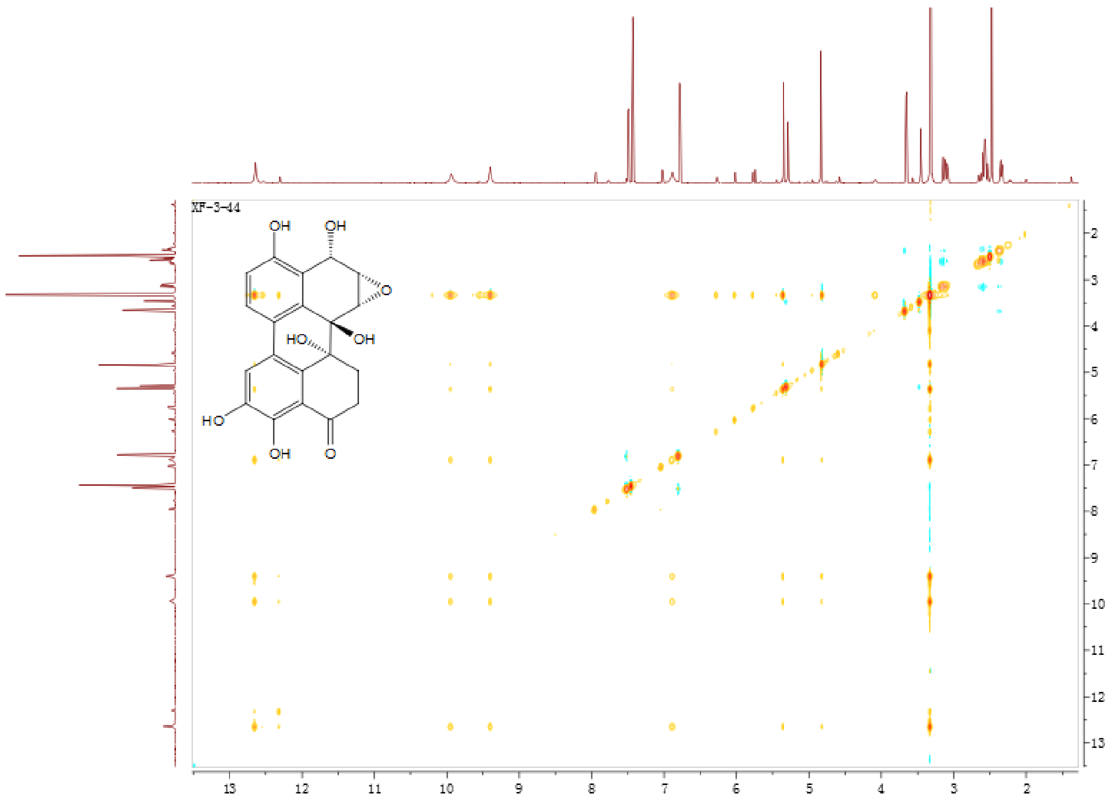

Figure S46. NOESY spectrum (600 MHz) of **8** in DMSO-d_6.

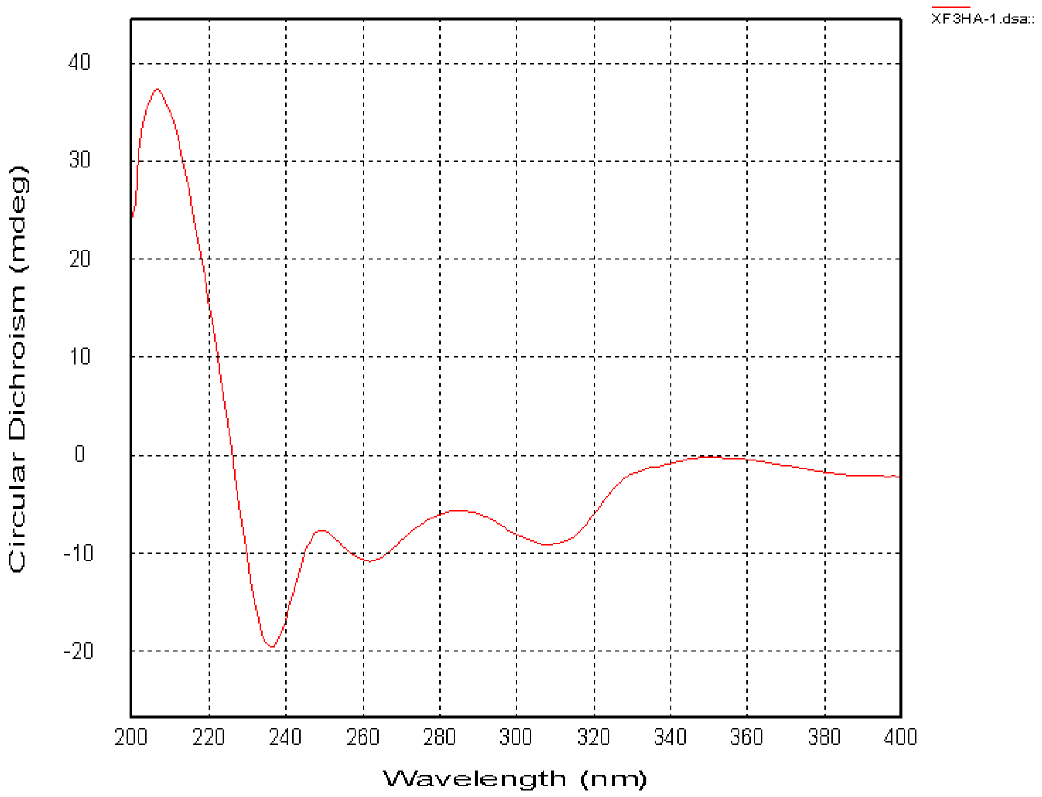

Figure S47. CD spectrum of **8**.

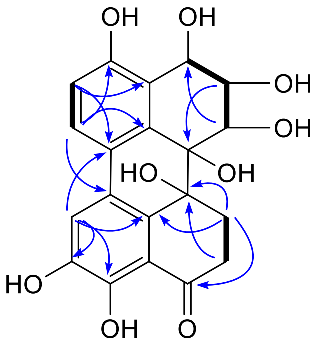

Figure S48. Key ^1H–^1H COSY (bold lines), HMBC (H→C) correlations for compound **9**.

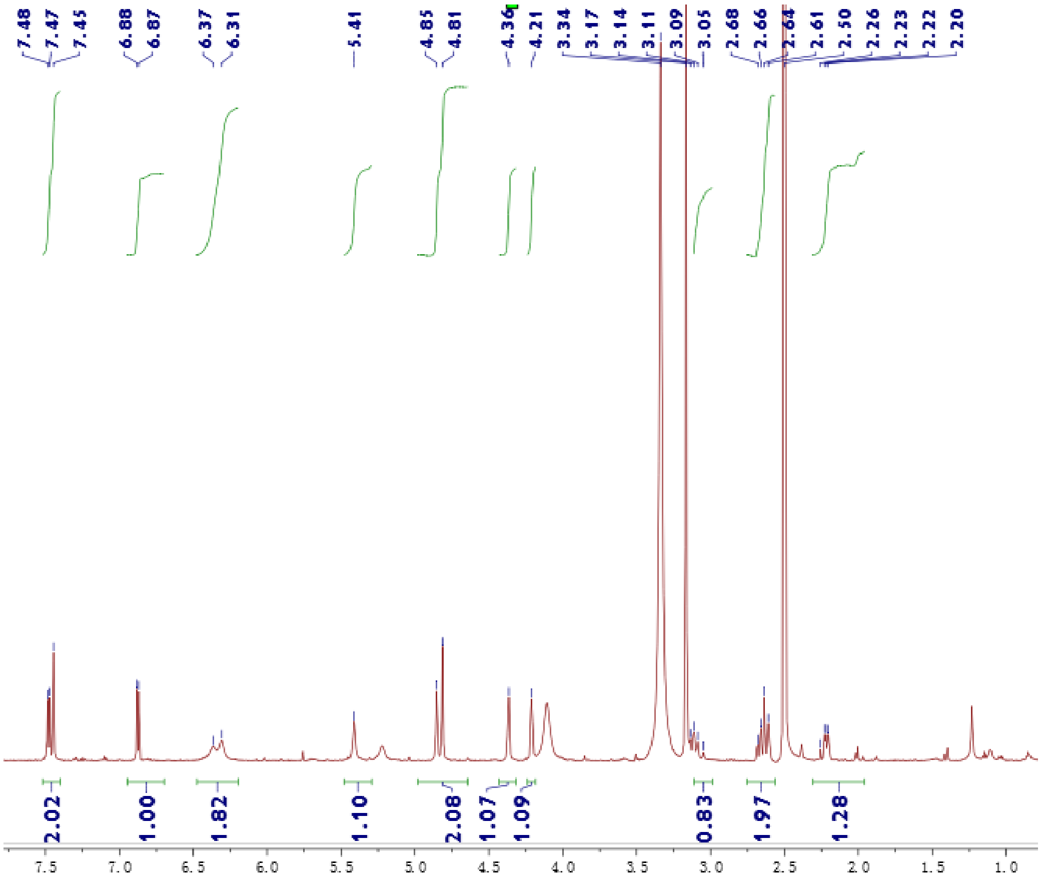

Figure S49. ^1H NMR spectrum (600 MHz) of **9** in DMSO-d_6.

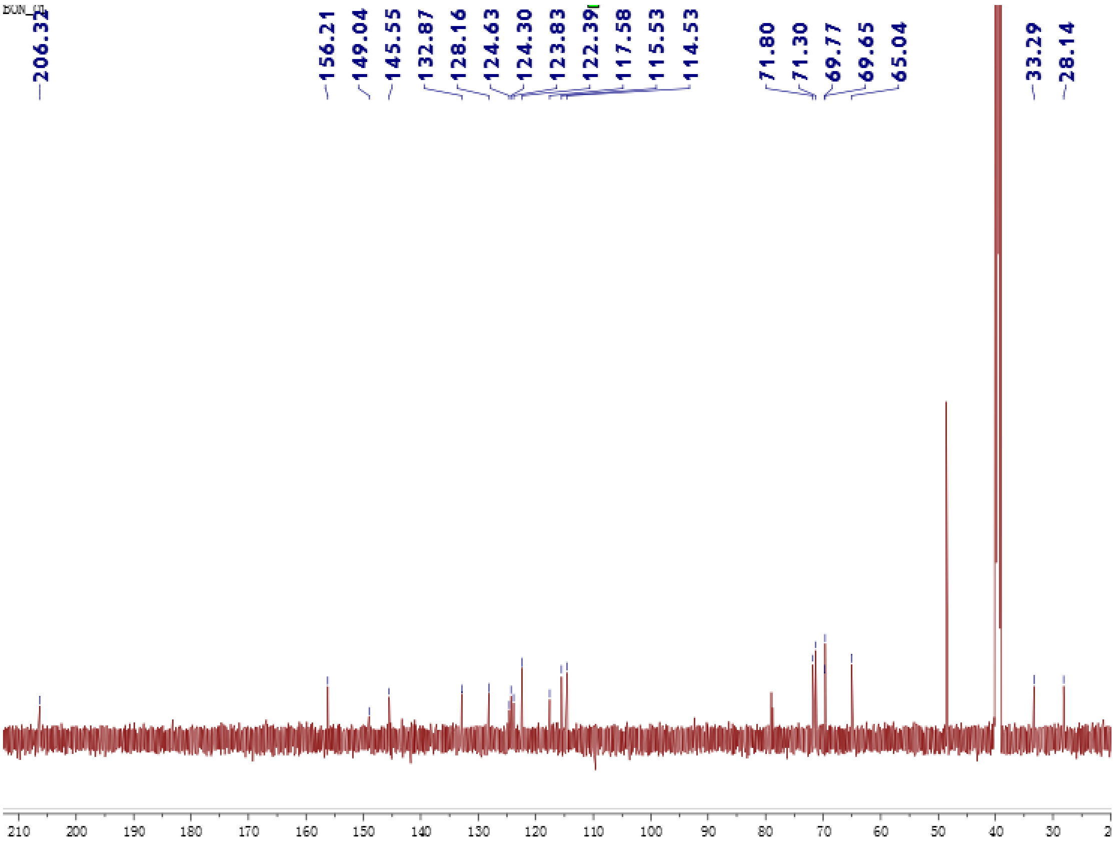

Figure S50. ^{13}C NMR spectrum (150 MHz) of **9** in DMSO-d_6.

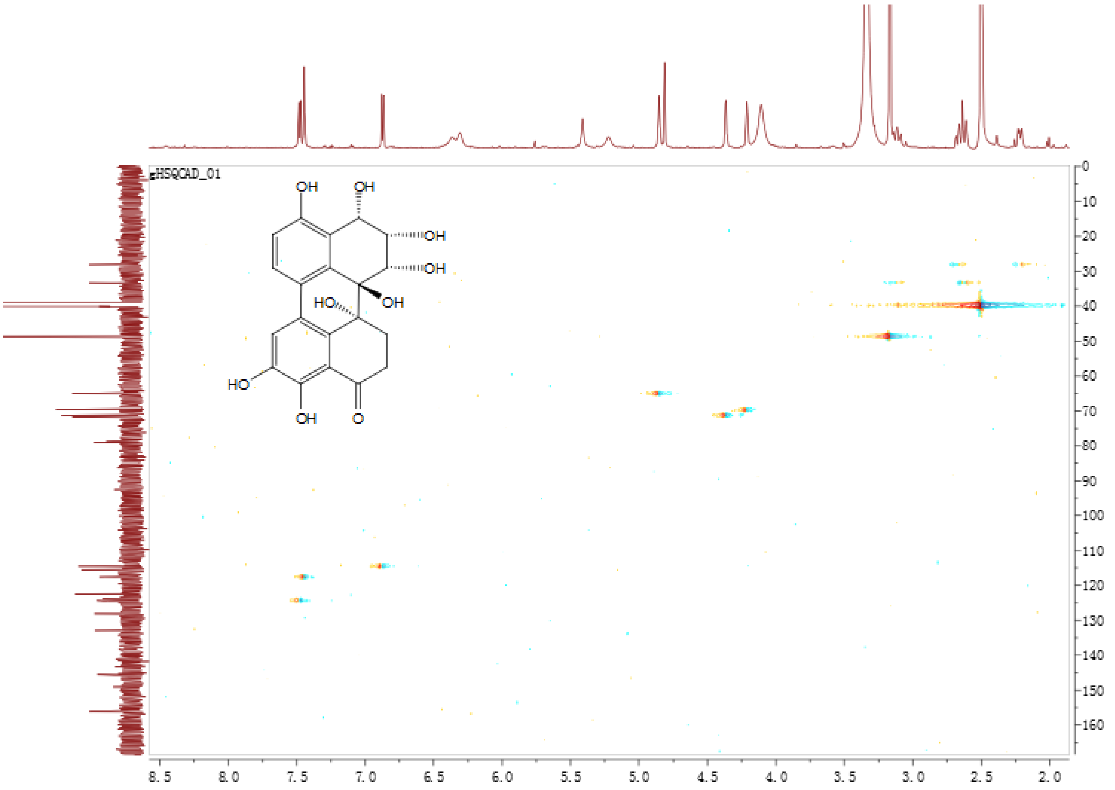

Figure S51. HSQC spectrum (600 MHz) of **9** in DMSO-d_6.

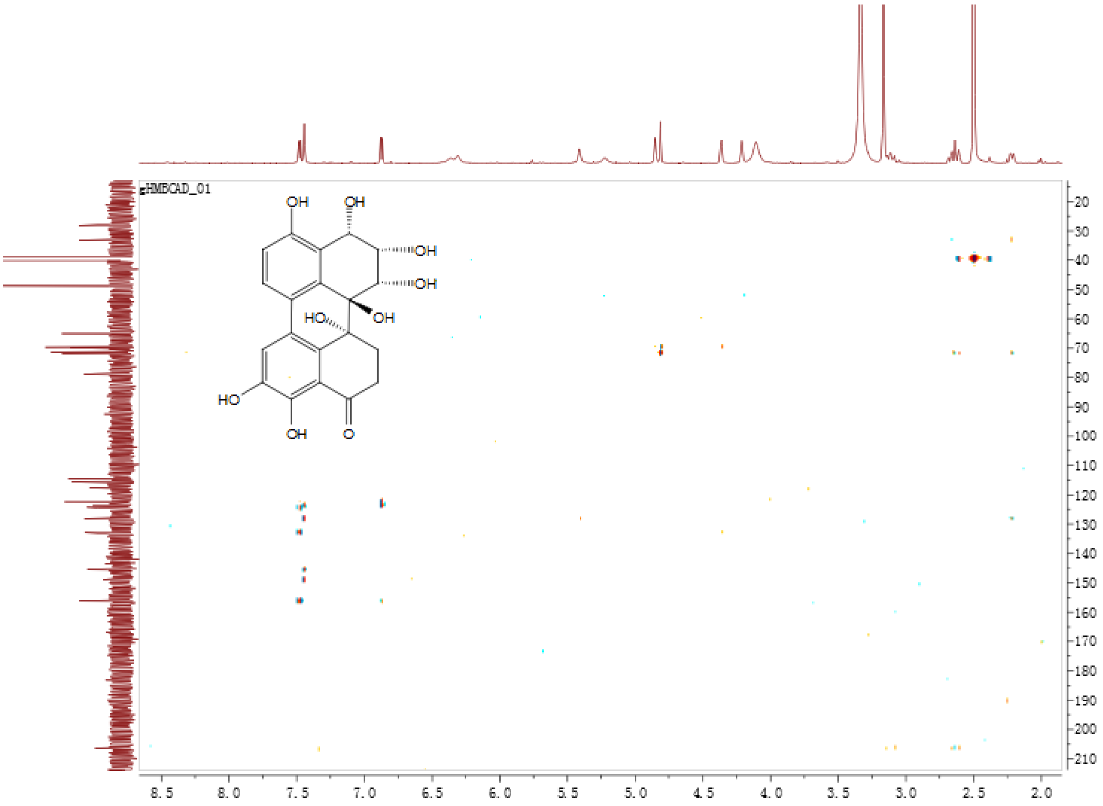

Figure S52. HMBC spectrum (600 MHz) of **9** in DMSO-d_6.

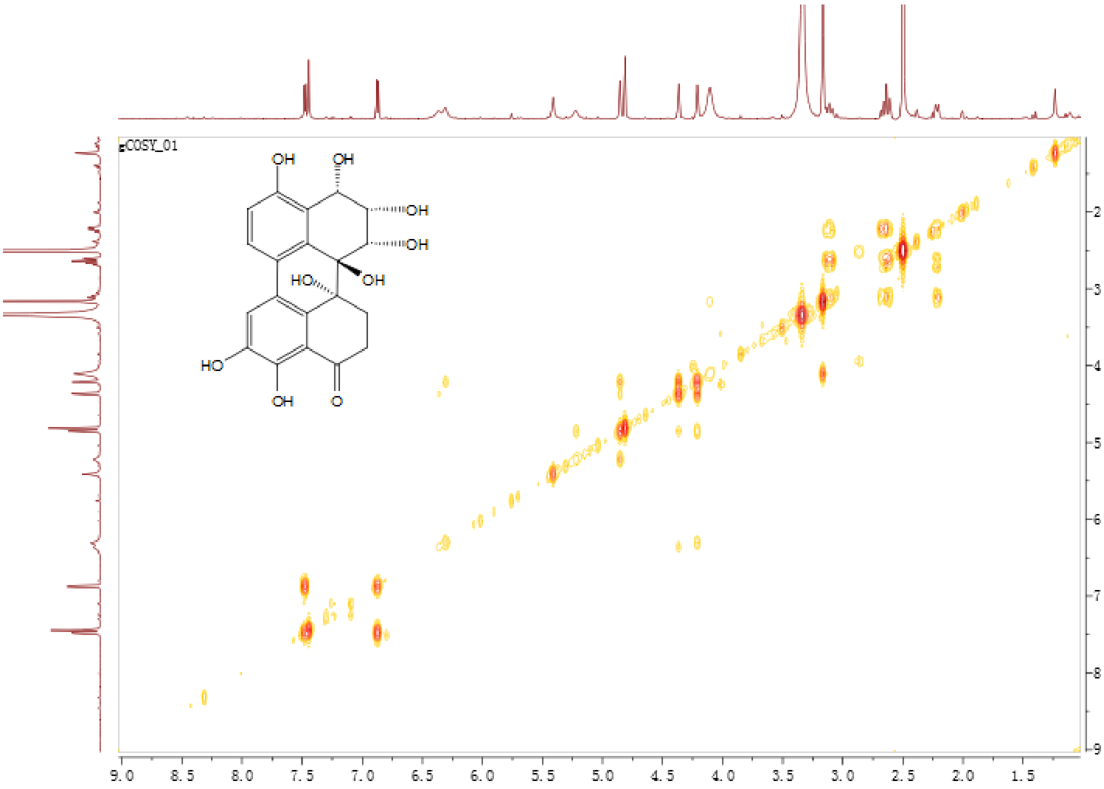

Figure S53. ^{1}H-^{1}H COSY spectrum (600 MHz) of **9** in DMSO-d_6.

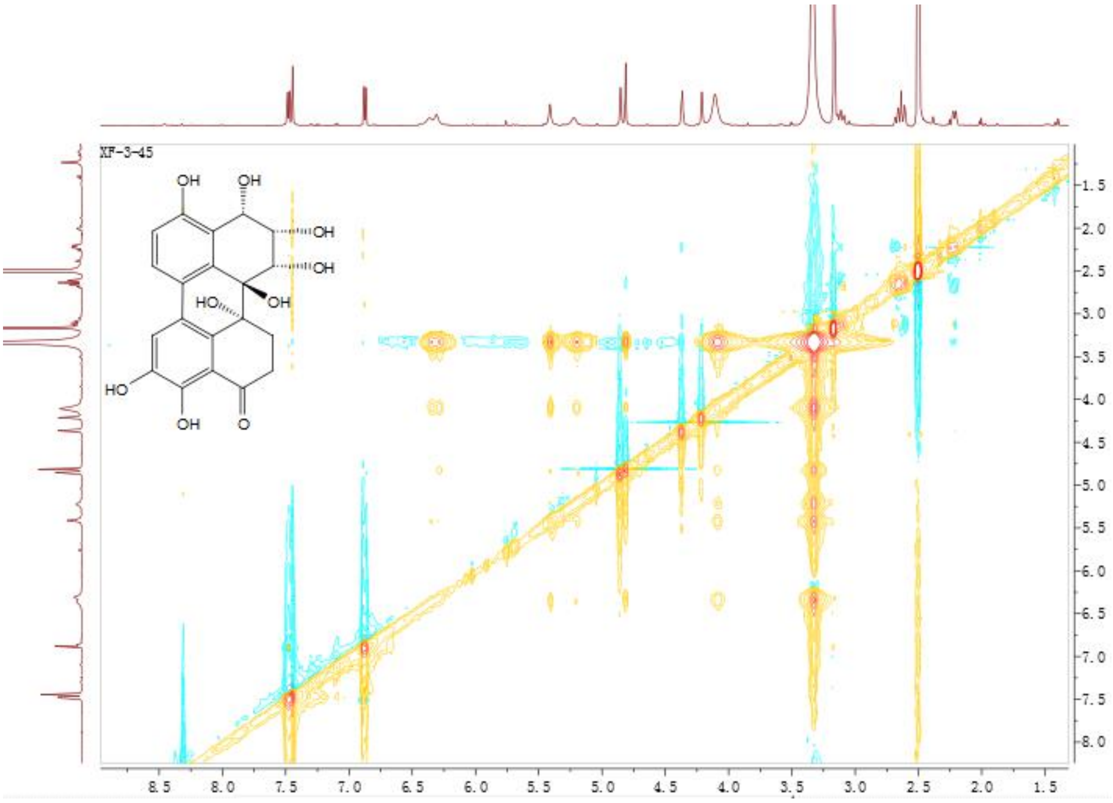

Figure S54. NOESY spectrum (600 MHz) of **9** in DMSO-d_6.

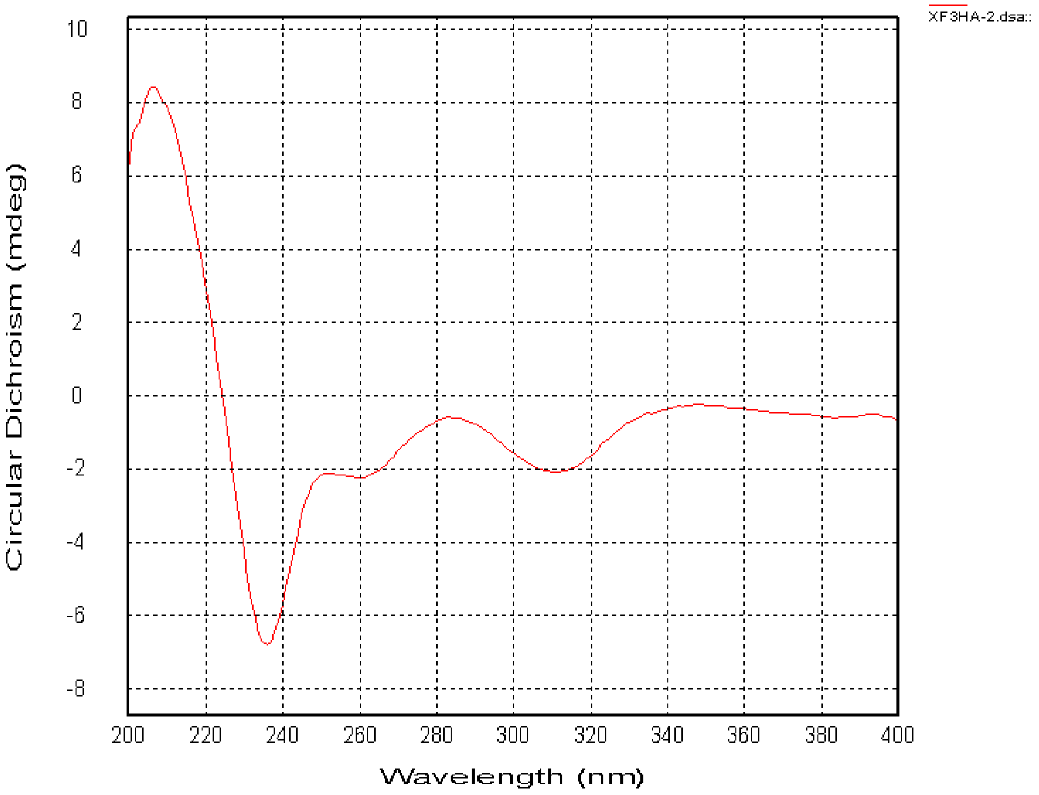

Figure S55. CD spectrum of **9**.

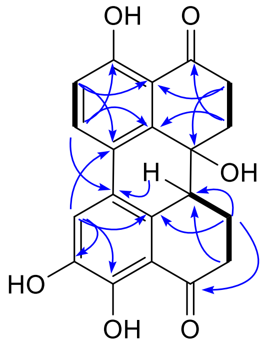

Figure S56. Key ${}^{1}H$–${}^{1}H$ COSY (bold lines), HMBC (H→C) correlations for compound **10**.

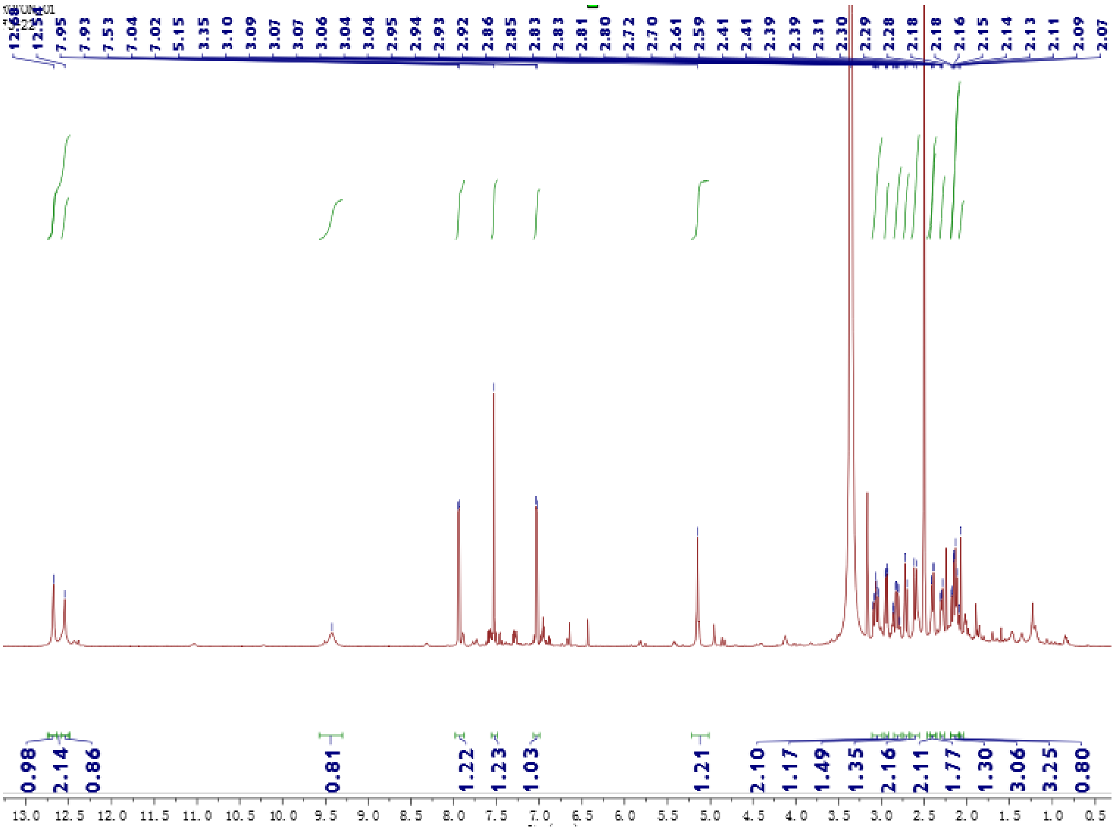

Figure S57. ${}^{1}H$ NMR spectrum (600 MHz) of **10** in DMSO-d_6.

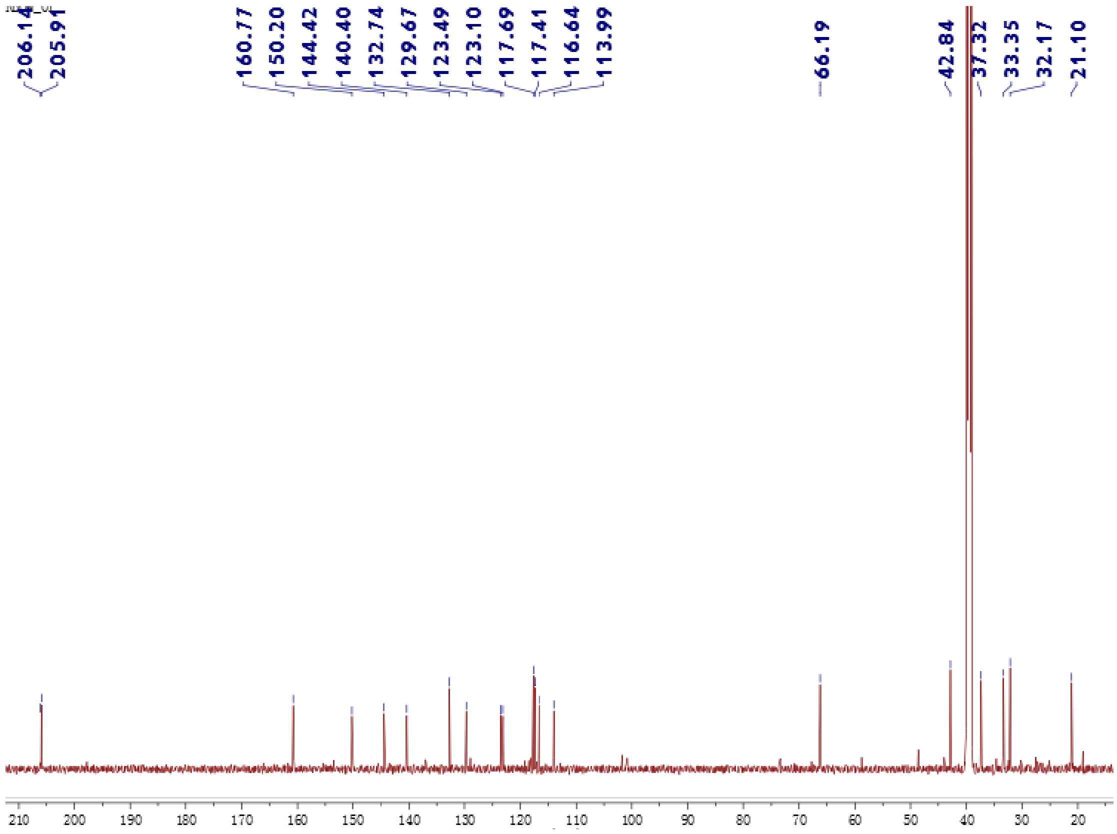

Figure S58. ^{13}C NMR spectrum (150 MHz) of **10** in DMSO-d_6.

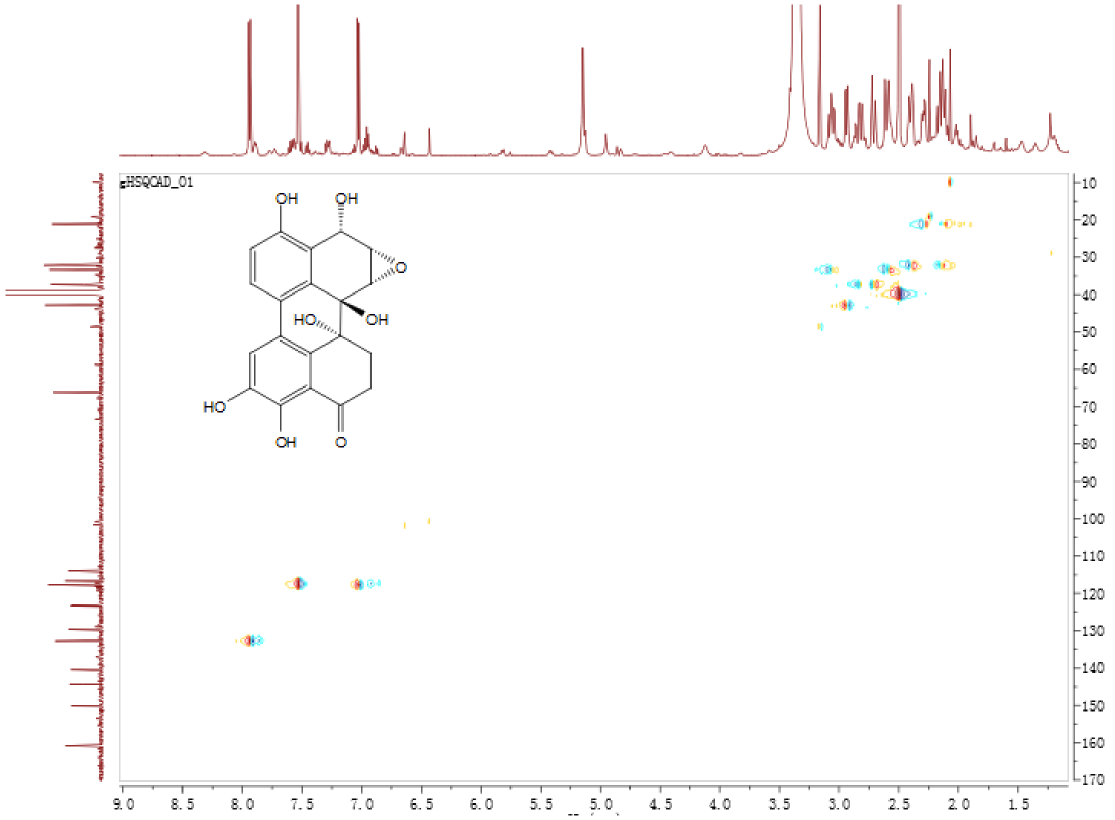

Figure S59. HSQC spectrum (600 MHz) of **10** in DMSO-d_6.

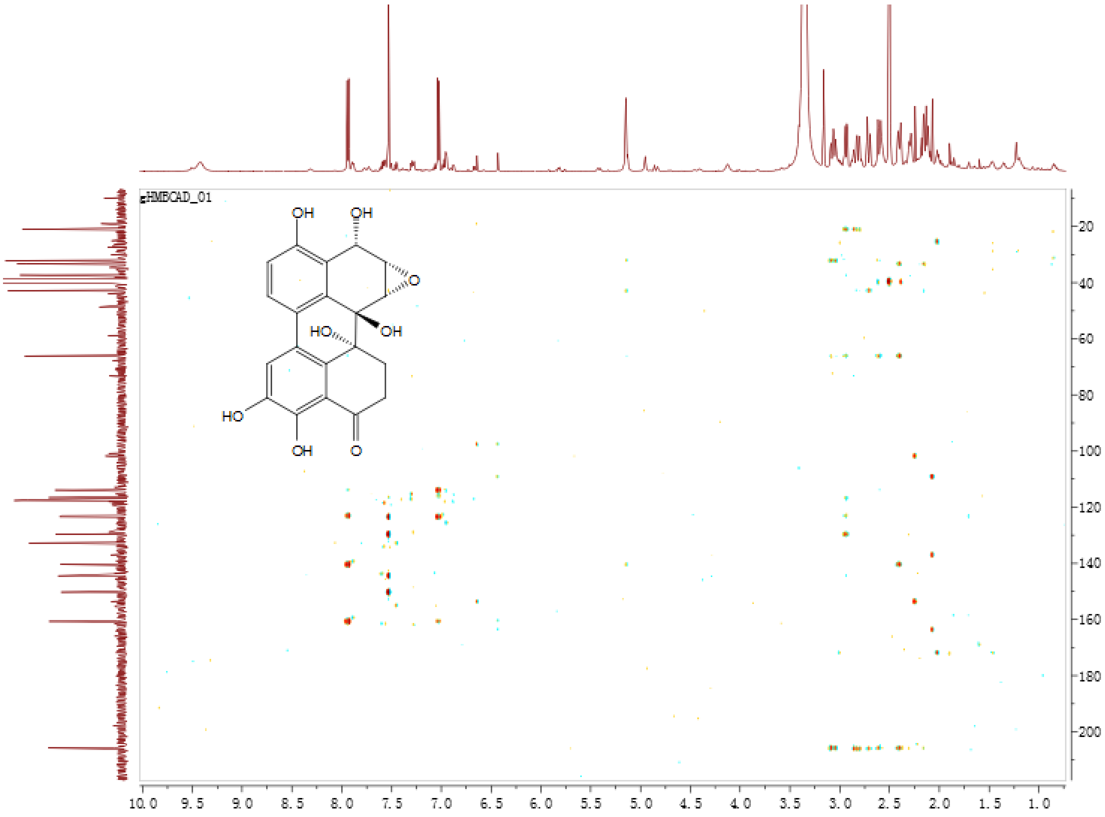

Figure S60. HMBC spectrum (600 MHz) of **10** in DMSO-d_6.

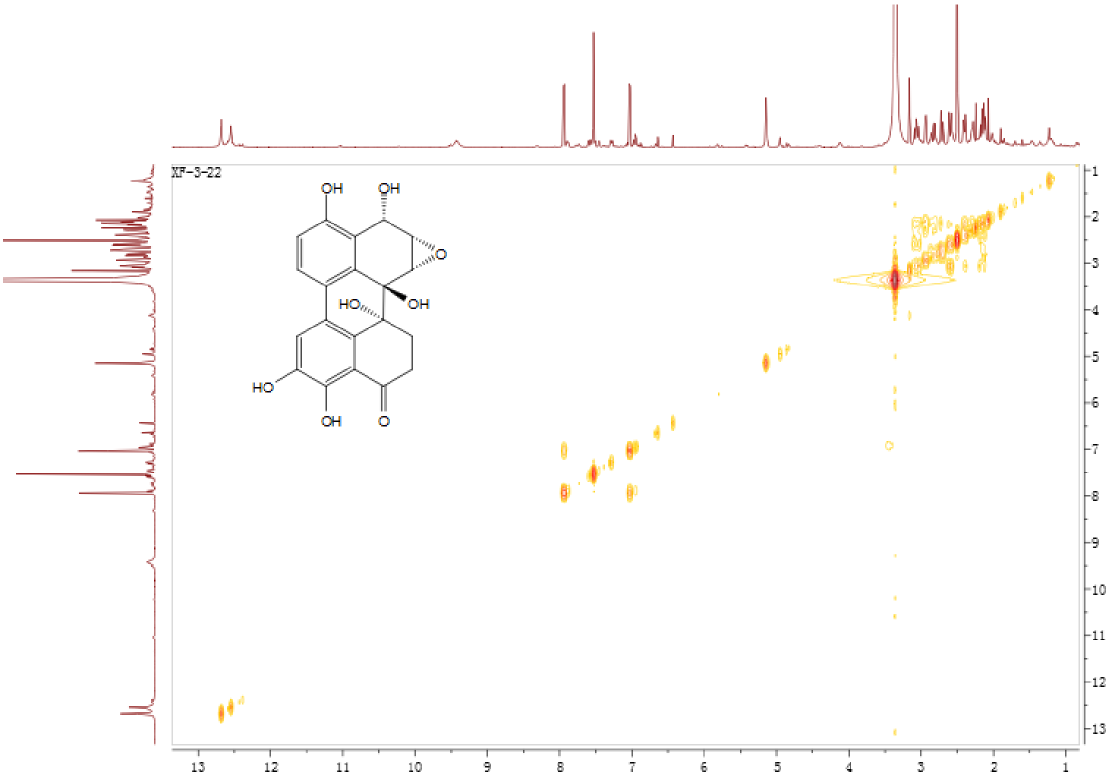

Figure S61. ^{1}H-^{1}H COSY spectrum (600 MHz) of **10** in DMSO-d_6.

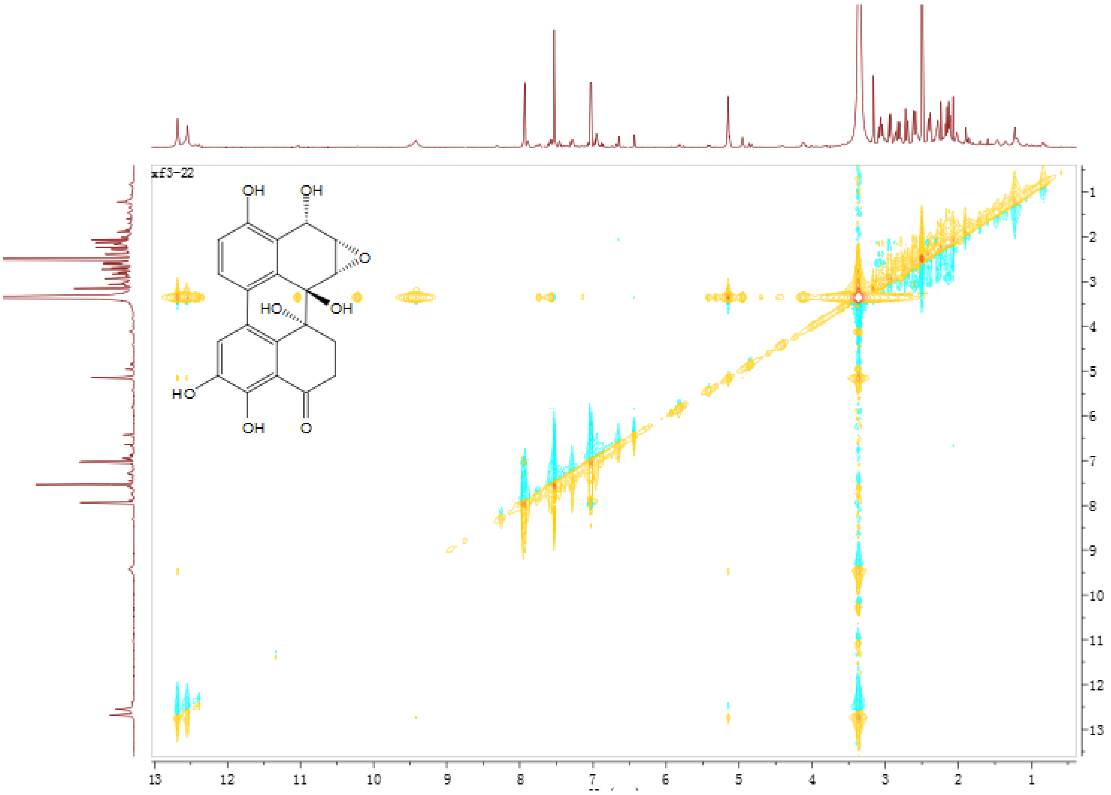

Figure S62. NOESY spectrum (600 MHz) of **10** in DMSO-d_6.

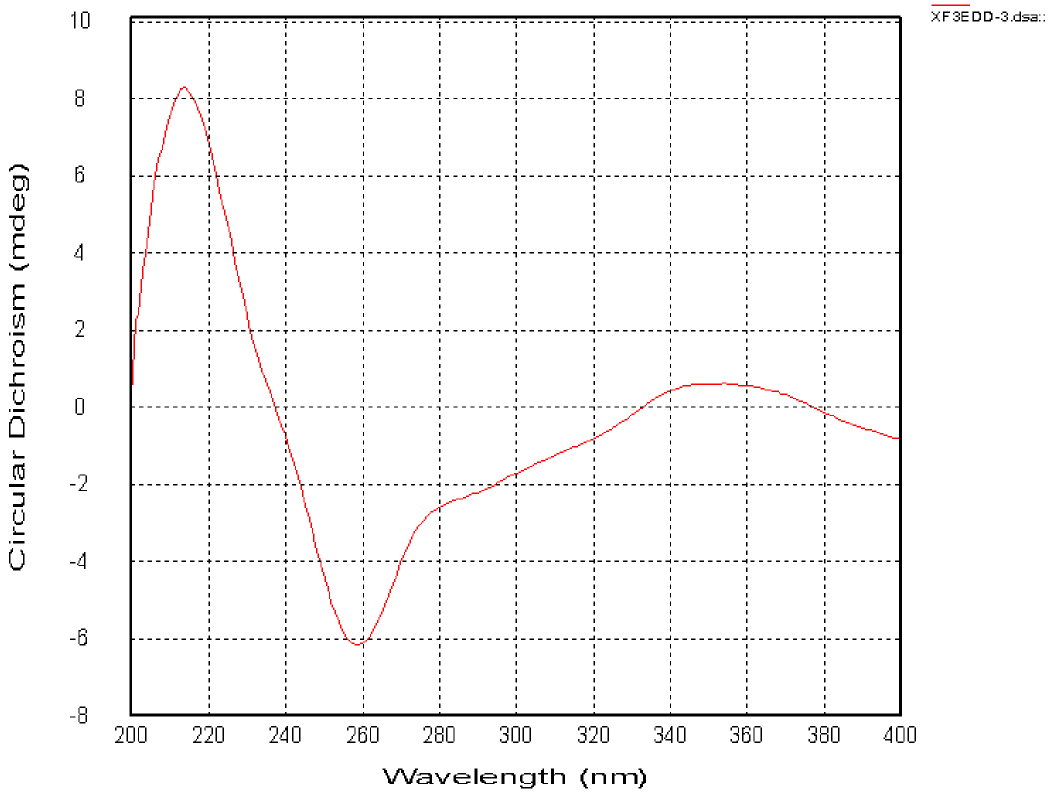

Figure S63. CD spectrum of **10**.

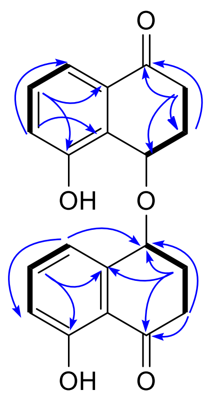

Figure S64. Key ${}^{1}H-{}^{1}H$ COSY (bold lines), HMBC (H→C) correlations for compound **11**.

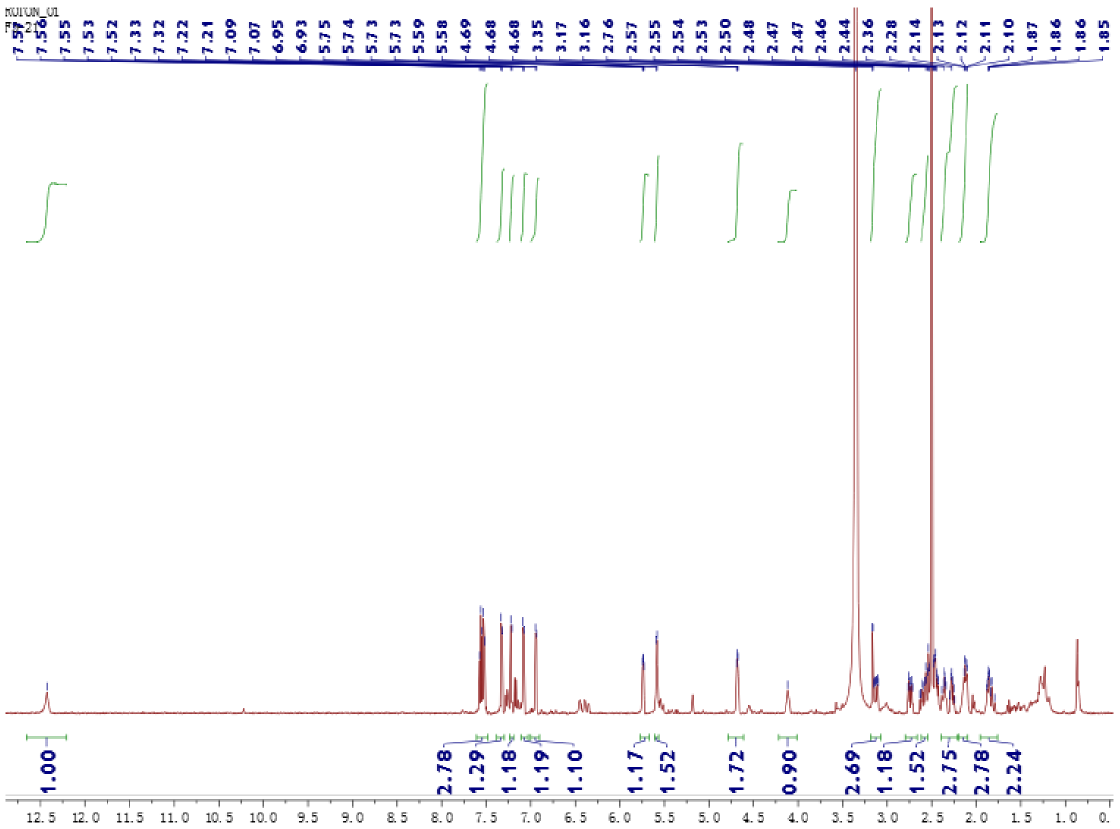

Figure S65. ${}^{1}H$ NMR spectrum (600 MHz) of **11** in DMSO-d_6.

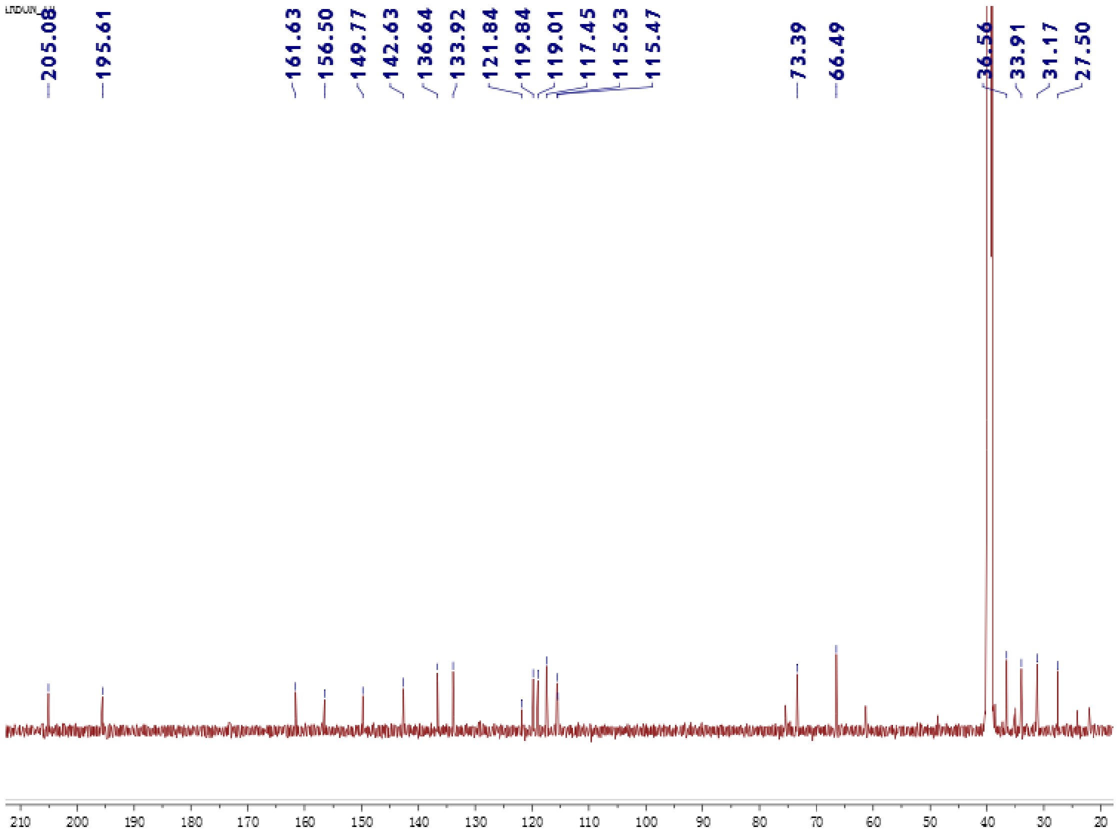

Figure S66. ^{13}C NMR spectrum (150 MHz) of **11** in DMSO-d_6.

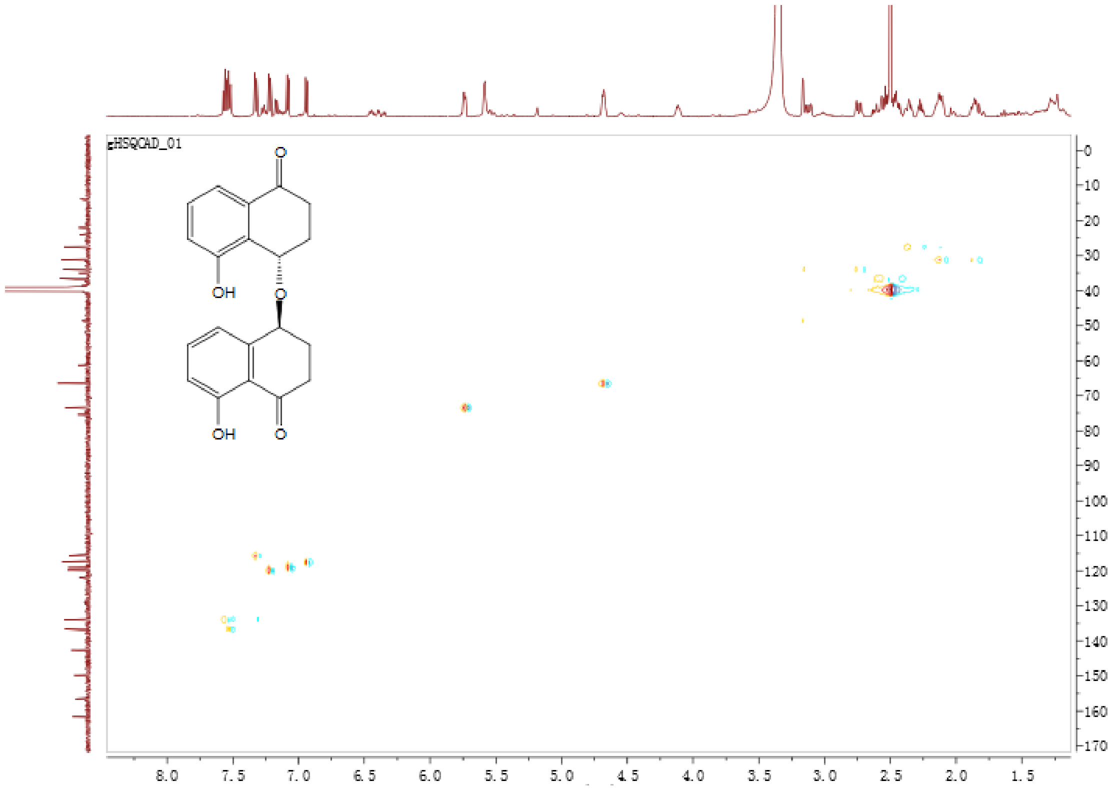

Figure S67. HSQC spectrum (600 MHz) of **11** in DMSO-d_6.

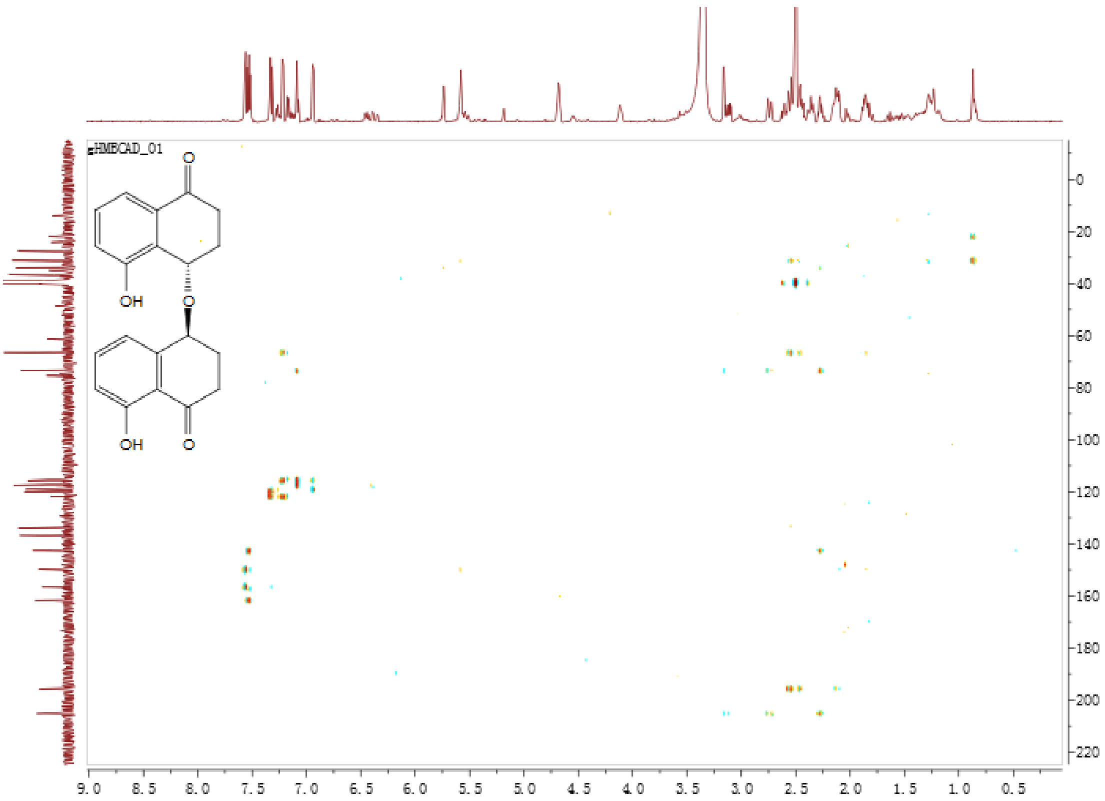

Figure S68. HMBC spectrum (600 MHz) of **11** in DMSO-d_6.

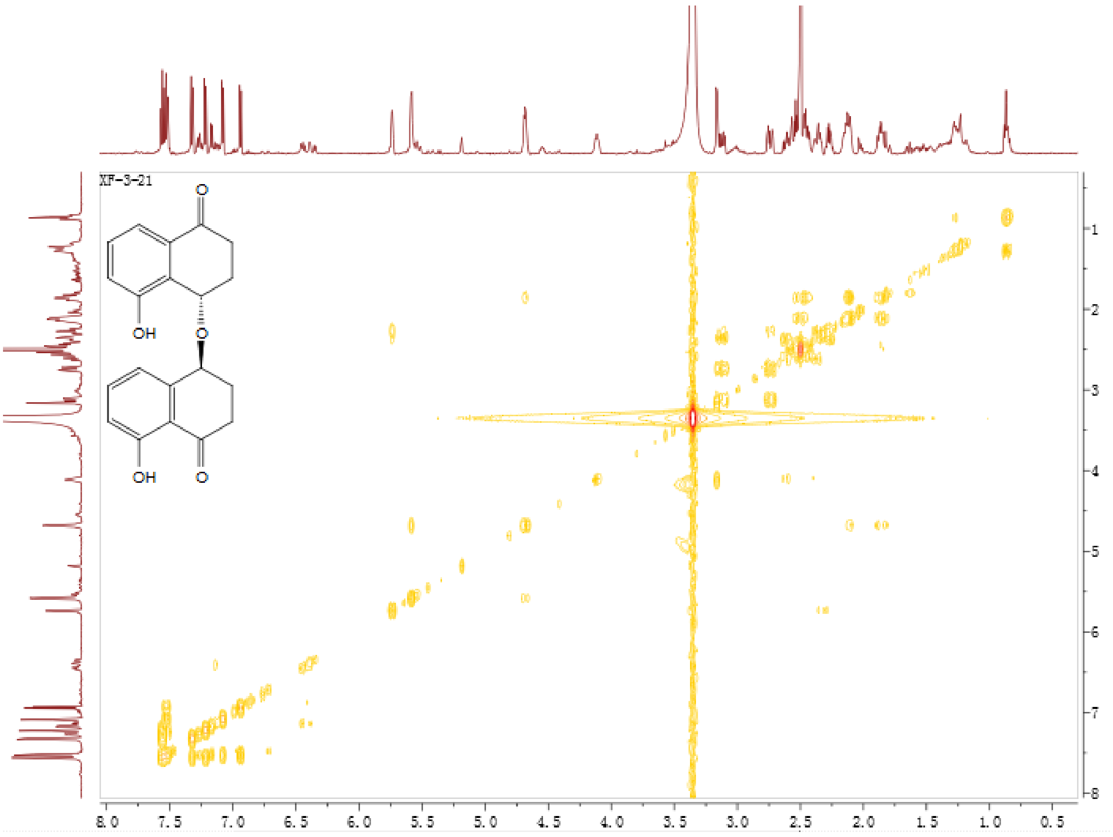

Figure S69. ^{1}H-^{1}H COSY spectrum (600 MHz) of **11** in DMSO-d_6.

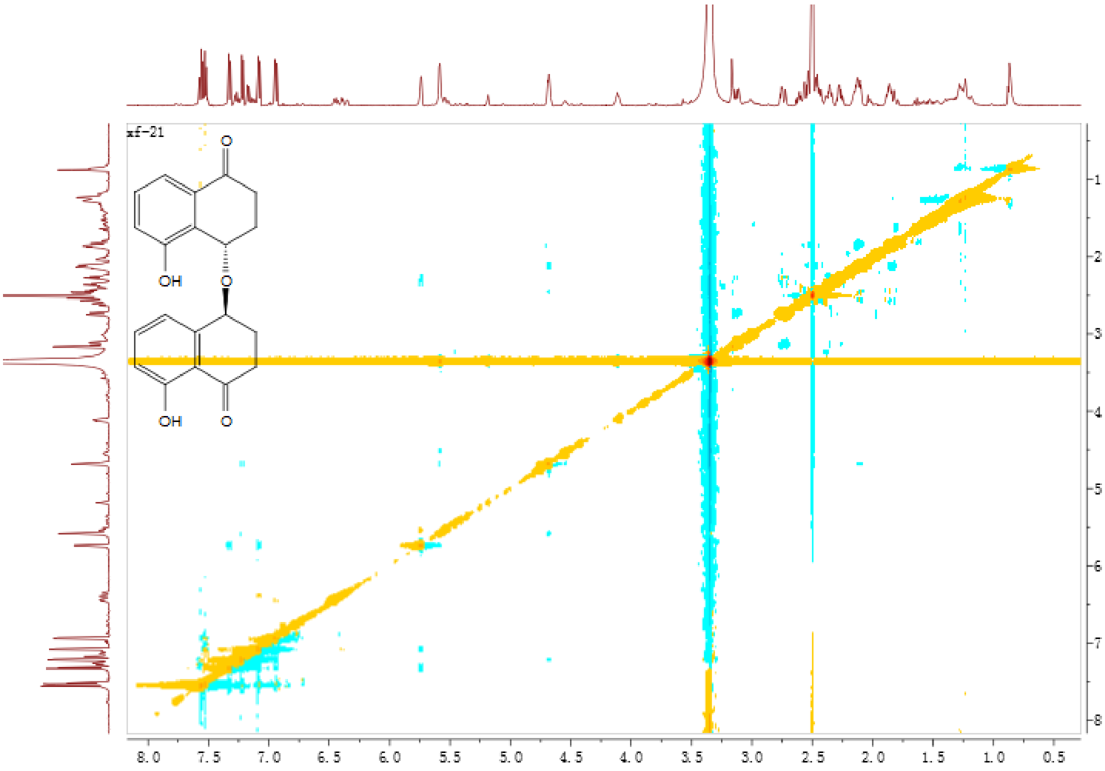

Figure S70. NOESY spectrum (600 MHz) of **11** in DMSO-d_6.

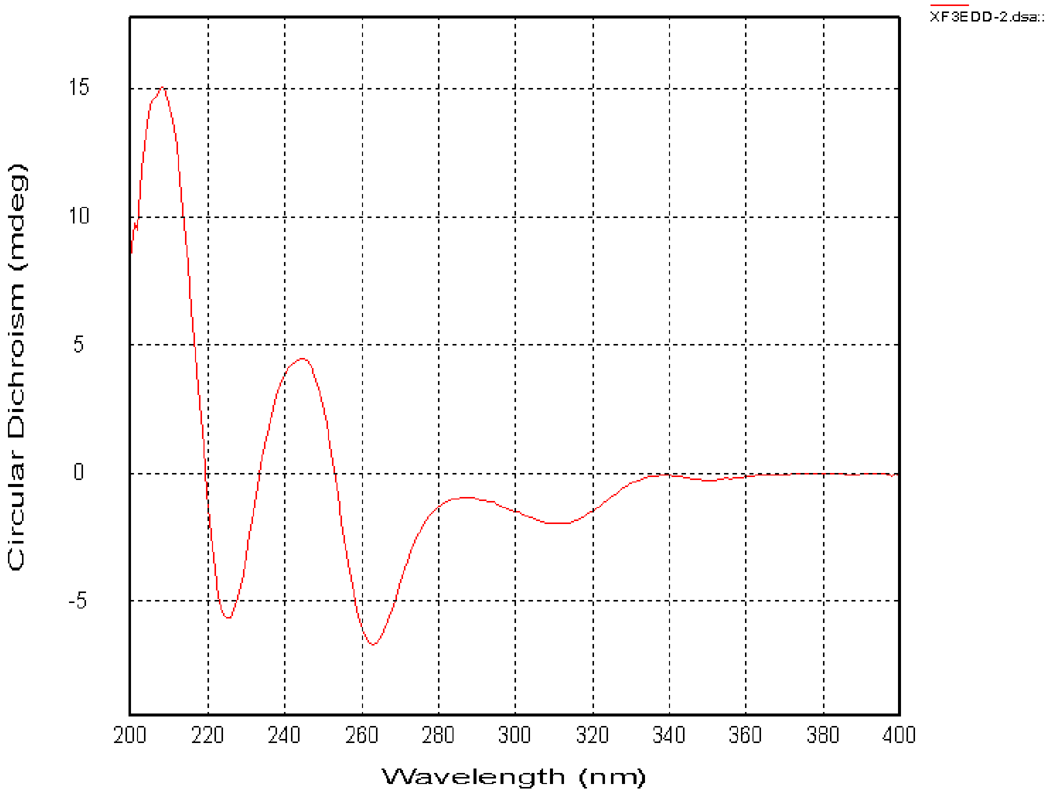

Figure S71. CD spectrum of **11**.

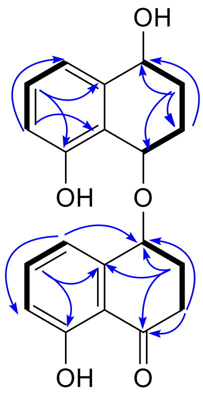

Figure S72. Key ^{1}H–^{1}H COSY (bold lines), HMBC (H→C) correlations for compound **12**.

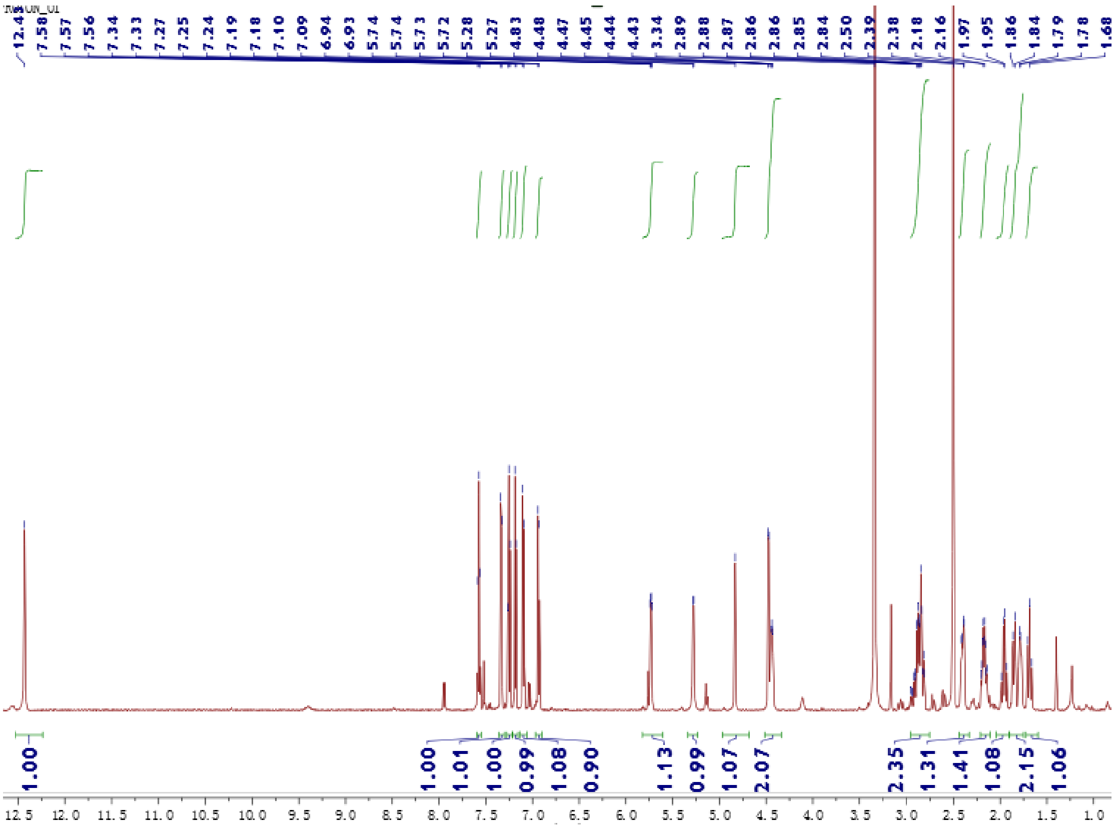

Figure S73. ^{1}H NMR spectrum (600 MHz) of **12** in DMSO-d_6.

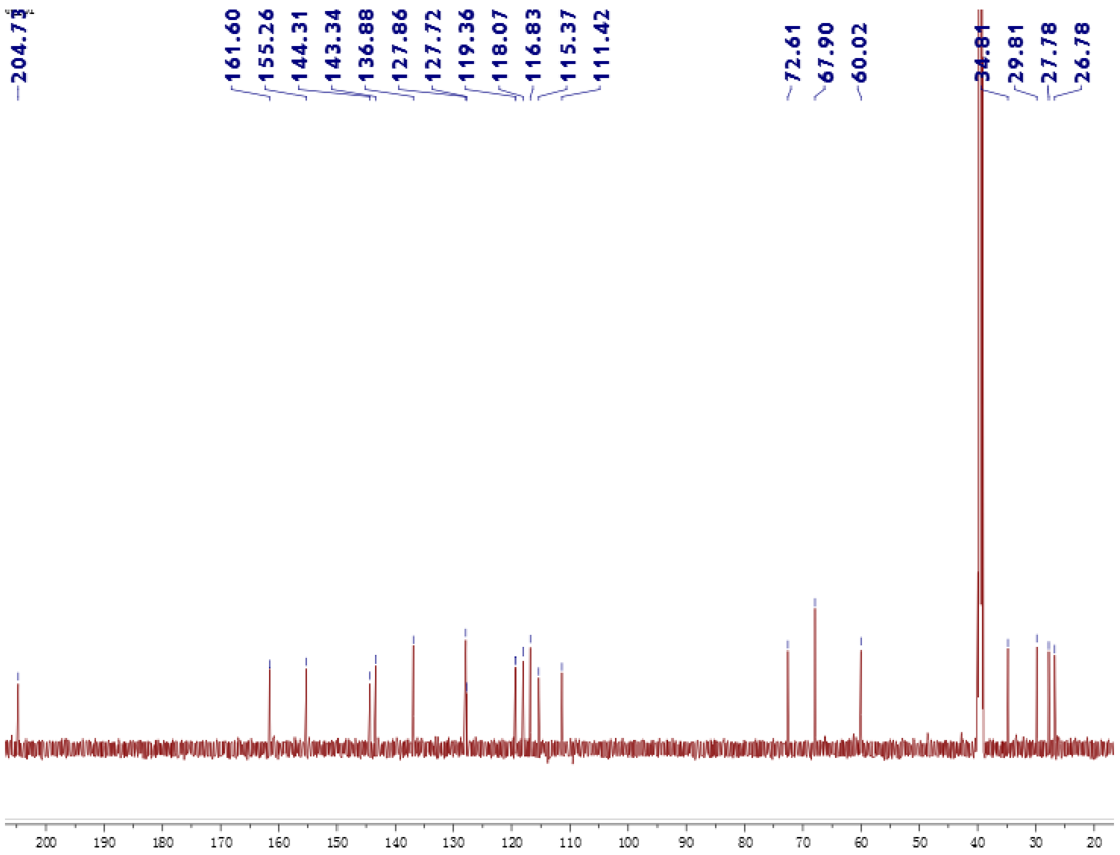

Figure S74. ^{13}C NMR spectrum (150 MHz) of **12** in DMSO-d_6.

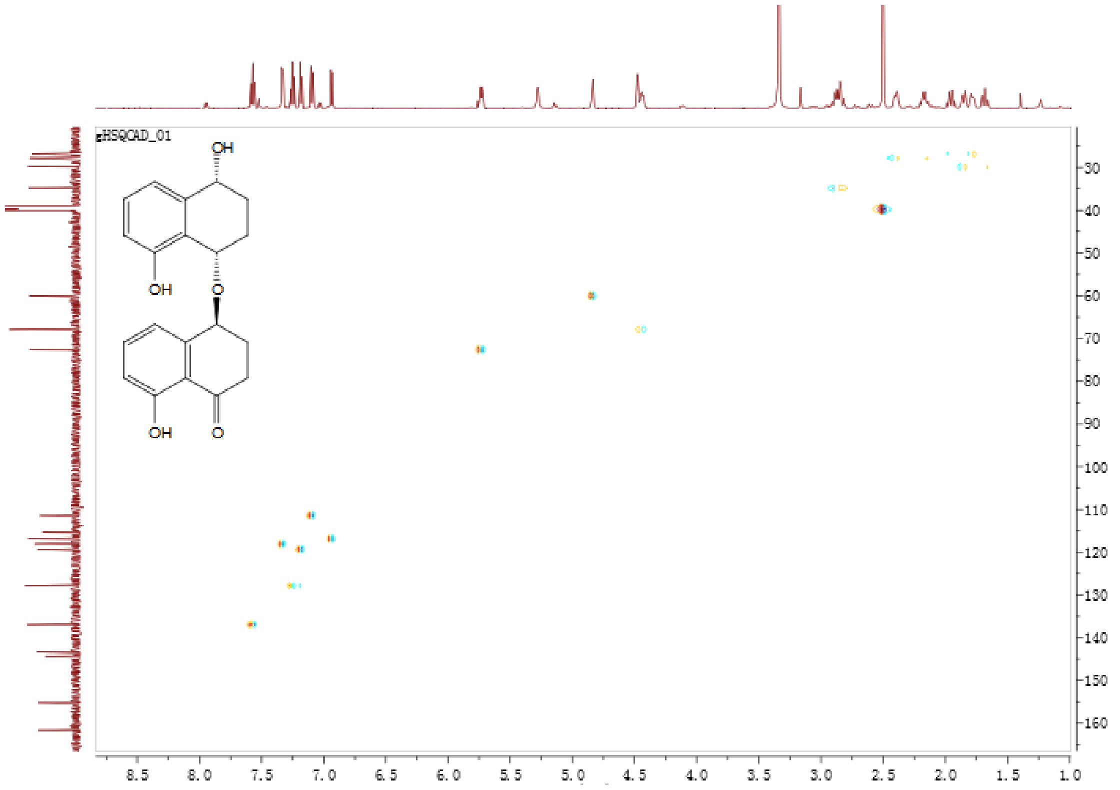

Figure S75. HSQC spectrum (600 MHz) of **12** in DMSO-d_6.

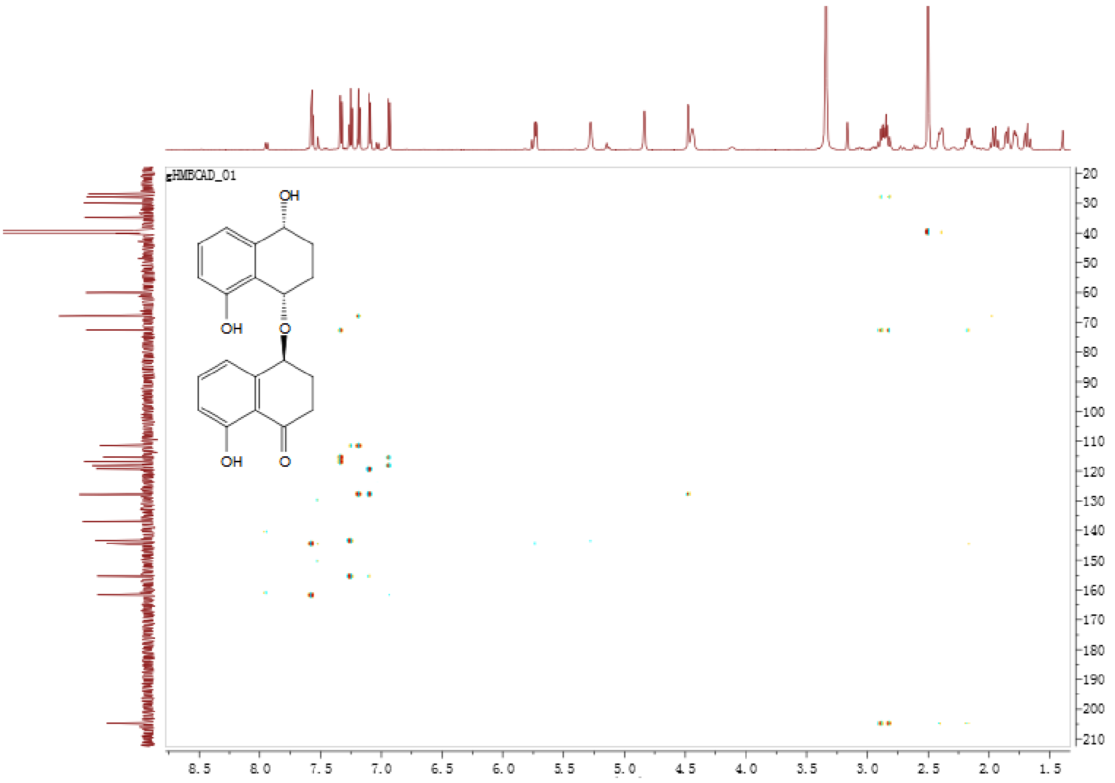

Figure S76. HMBC spectrum (600 MHz) of **12** in DMSO-d_6.

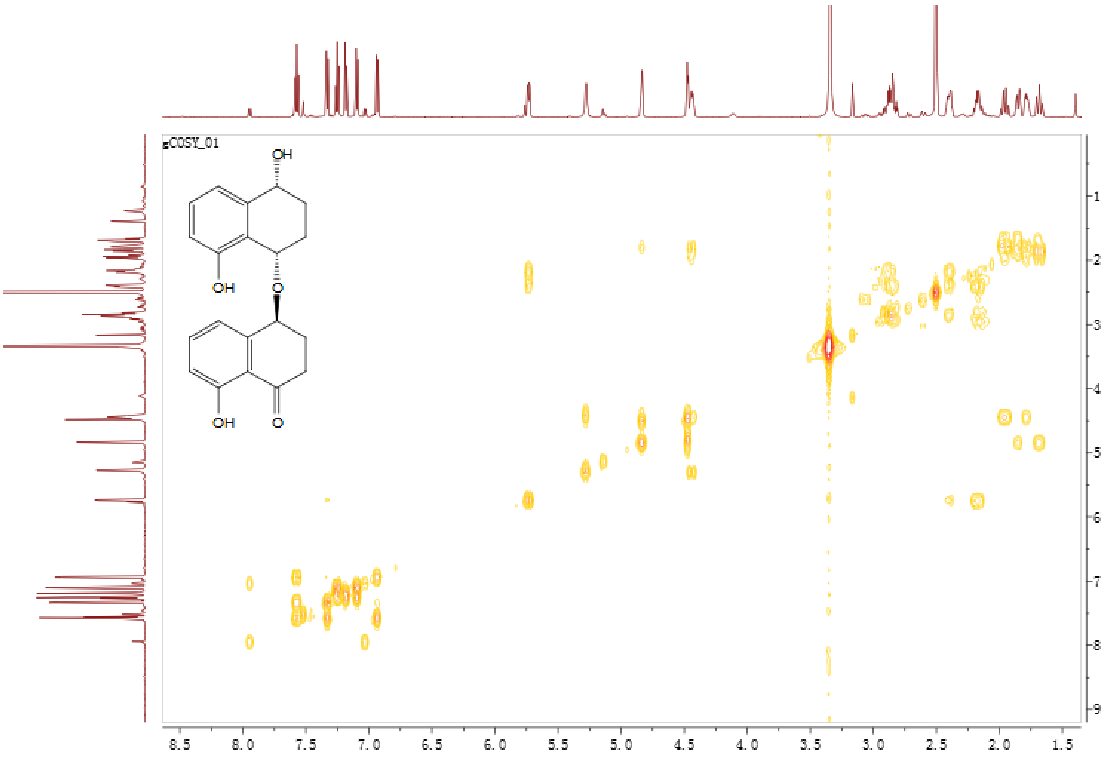

Figure S77. ^{1}H-^{1}H COSY spectrum (600 MHz) of **12** in DMSO-d_6.

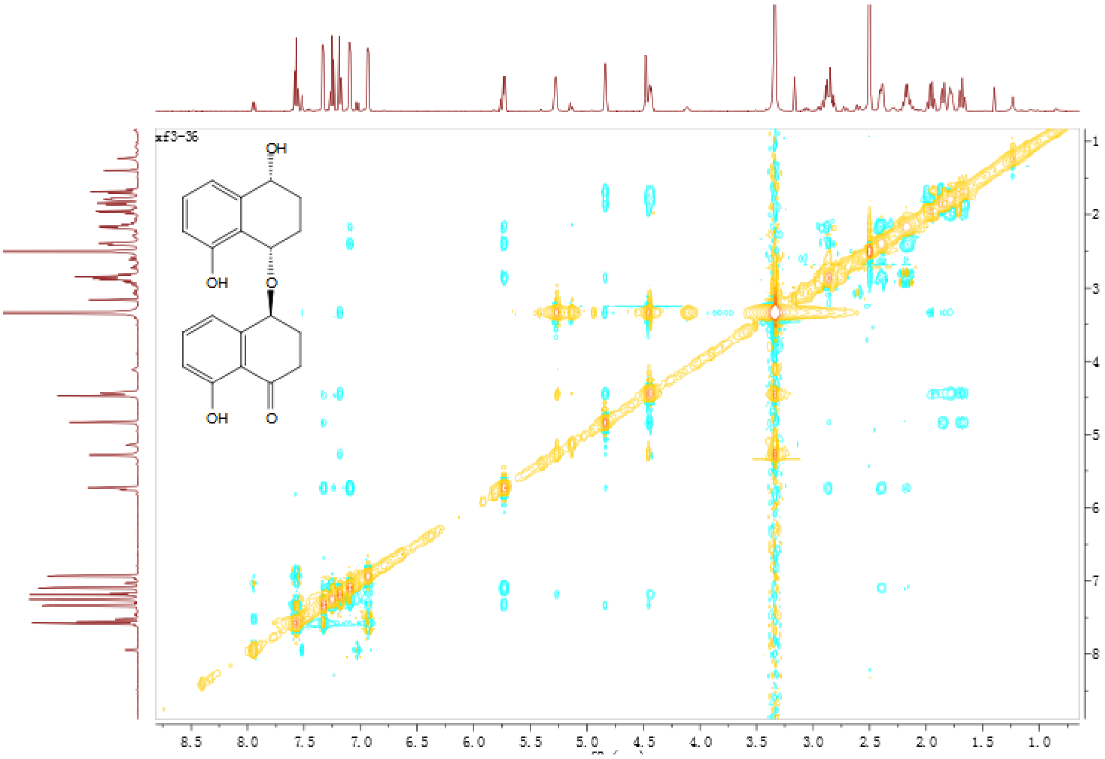

Figure S78. NOESY spectrum (600 MHz) of **12** in DMSO-d_6.

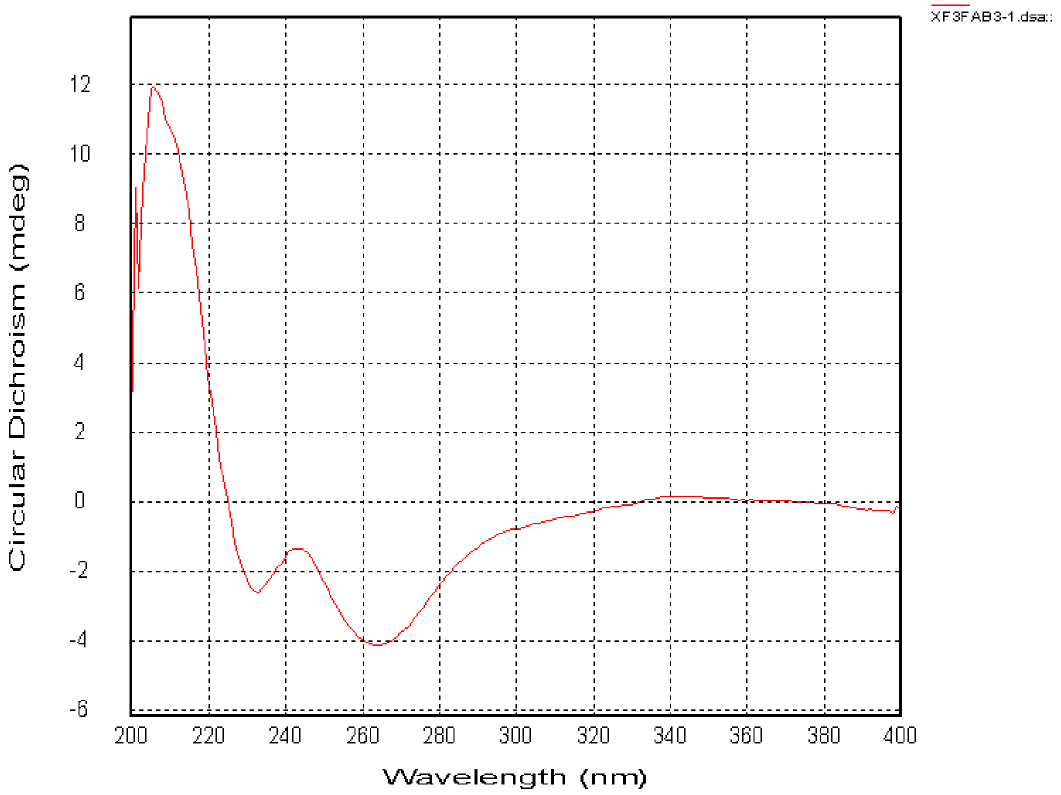

Figure S79. CD spectrum of **12**.

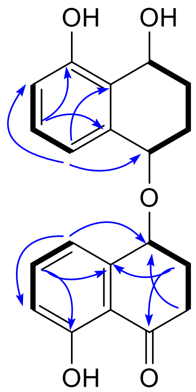

Figure S80. Key $^{1}H-^{1}H$ COSY (bold lines), HMBC (H→C) correlations for compound **13**.

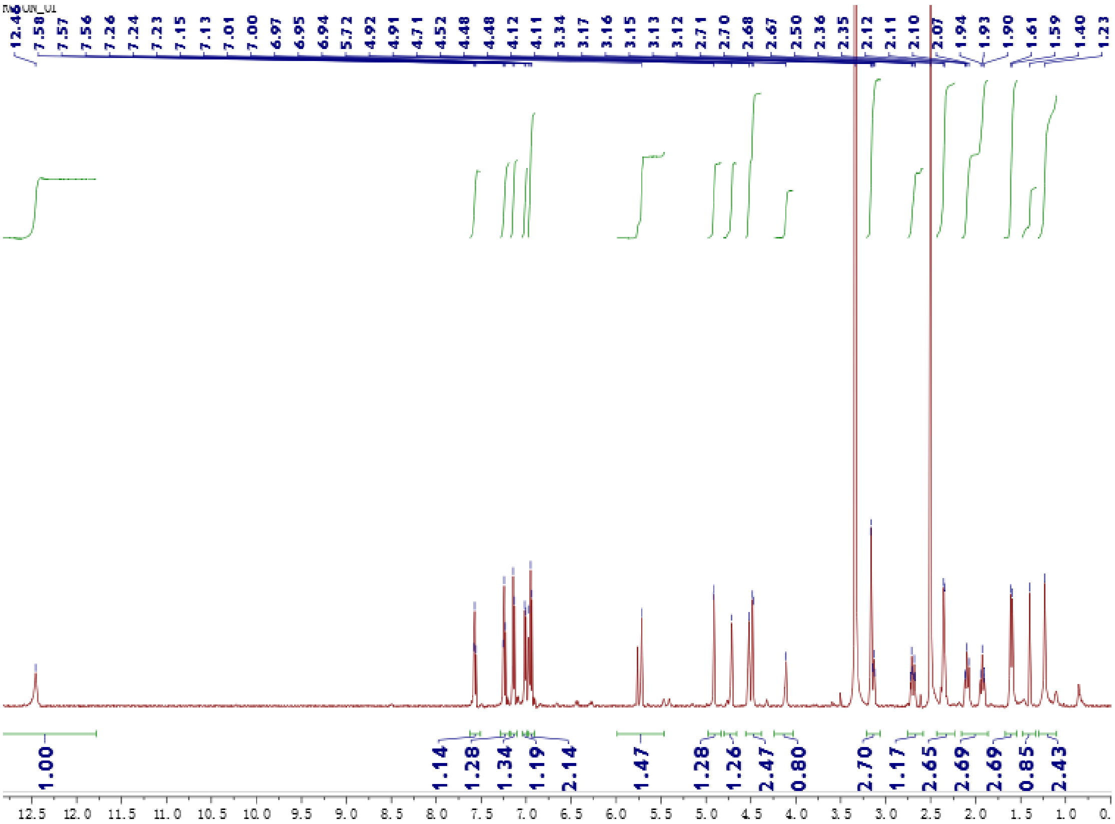

Figure S81. ^{1}H NMR spectrum (600 MHz) of **13** in DMSO-d_6.

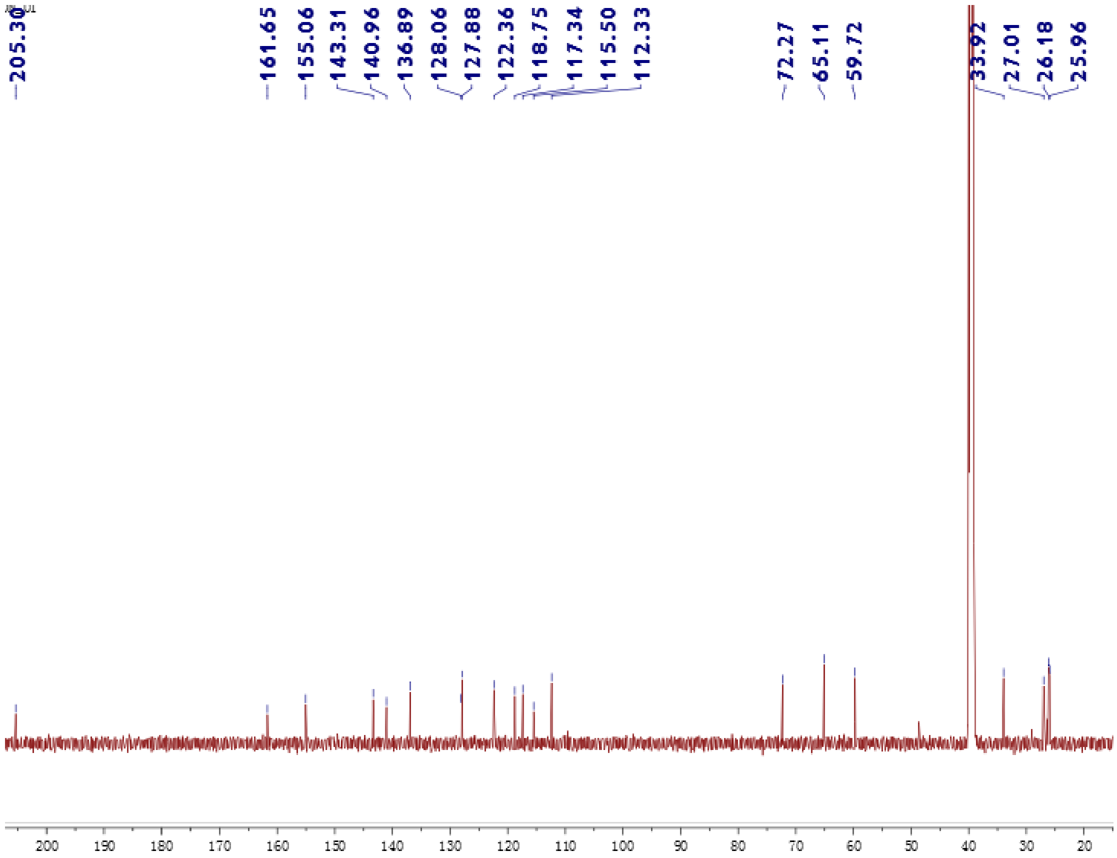

Figure S82. ^{13}C NMR spectrum (150 MHz) of **13** in DMSO-d_6.

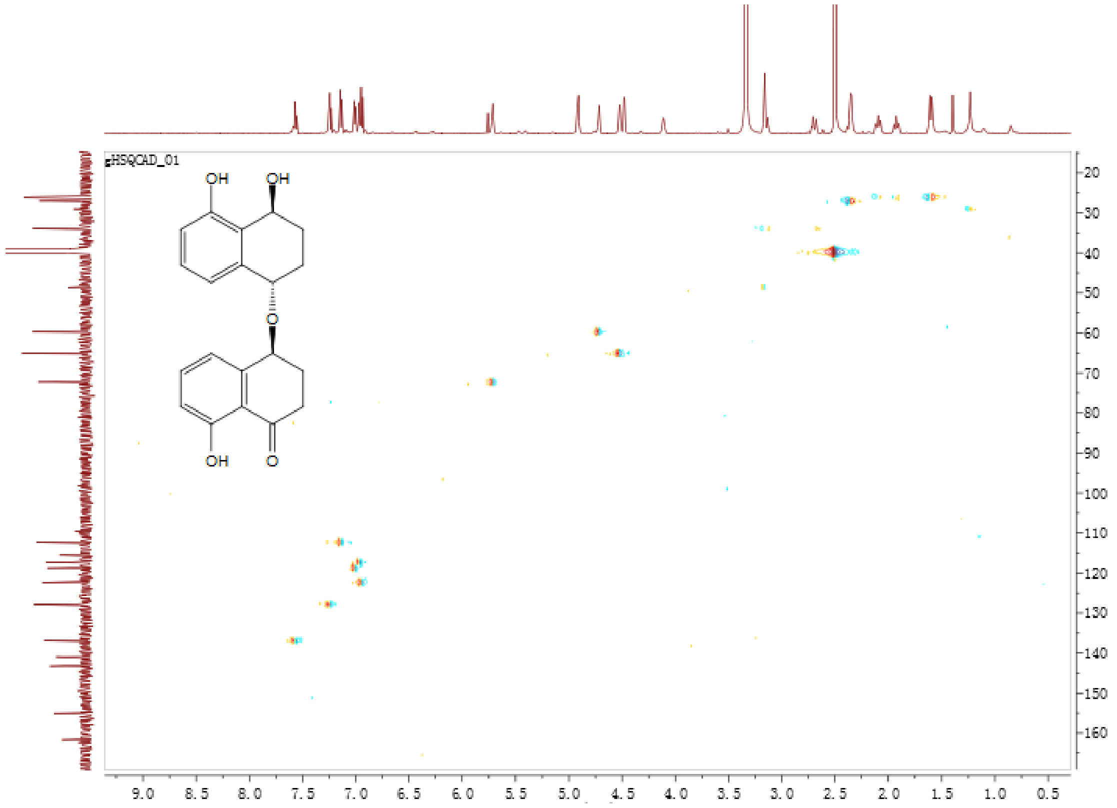

Figure S83. HSQC spectrum (600 MHz) of **13** in DMSO-d_6.

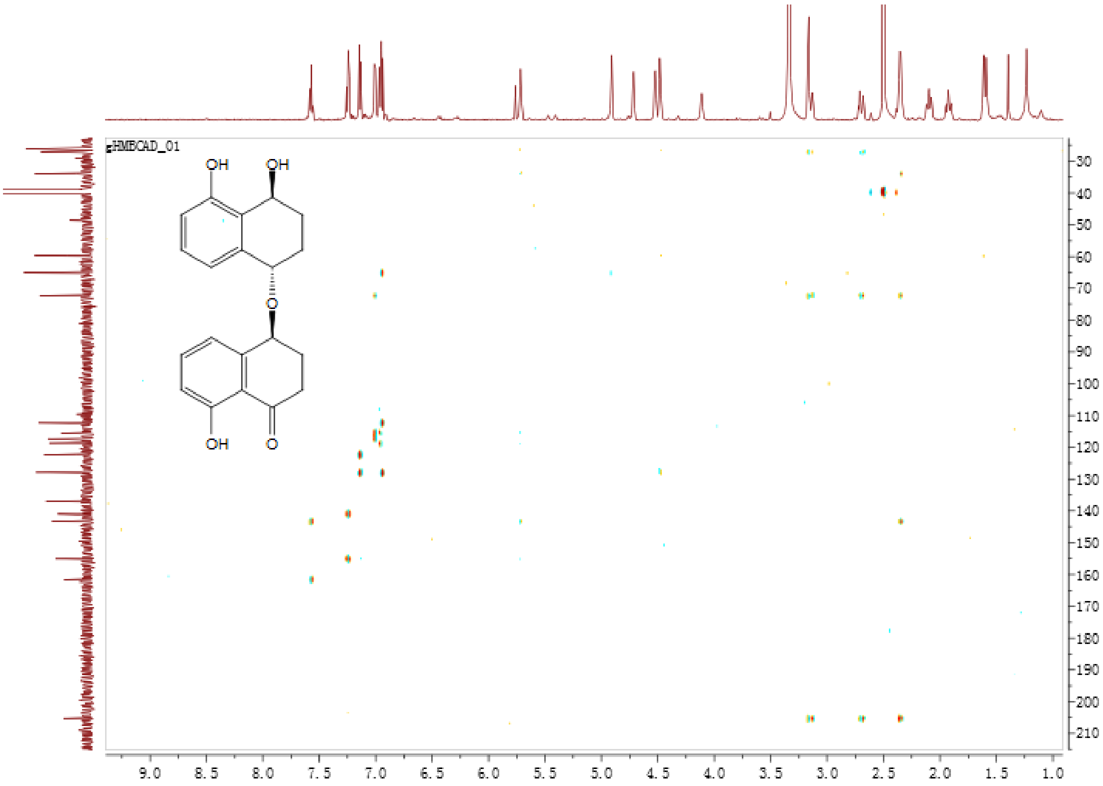

Figure S84. HMBC spectrum (600 MHz) of **13** in DMSO-d_6.

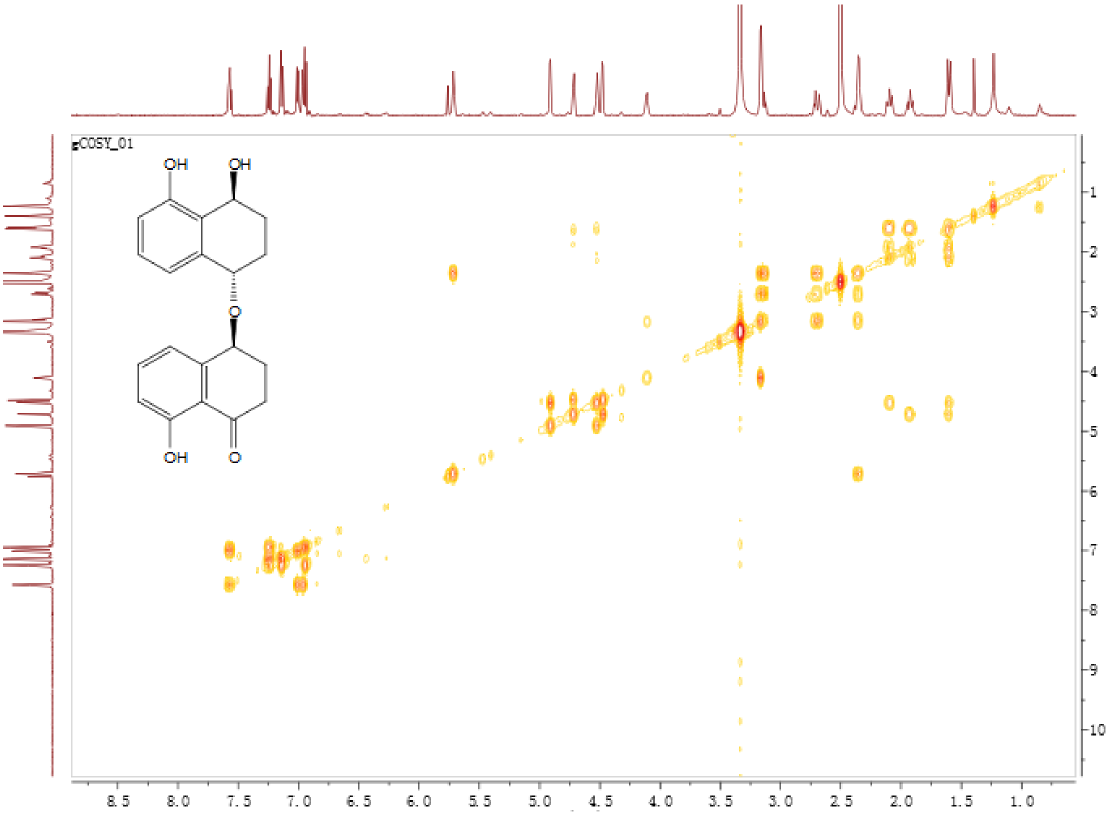

Figure S85. ^{1}H-^{1}H COSY spectrum (600 MHz) of **13** in DMSO-d_6.

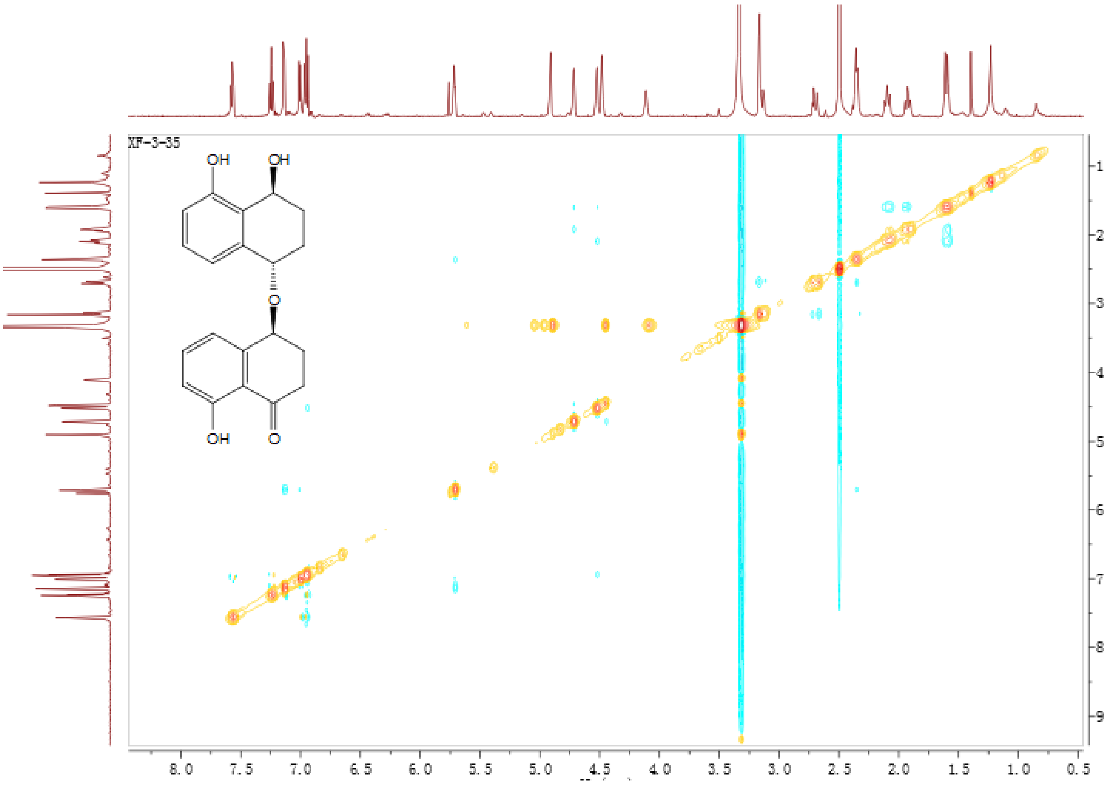

Figure S86. NOESY spectrum (600 MHz) of **13** in DMSO-d_6.

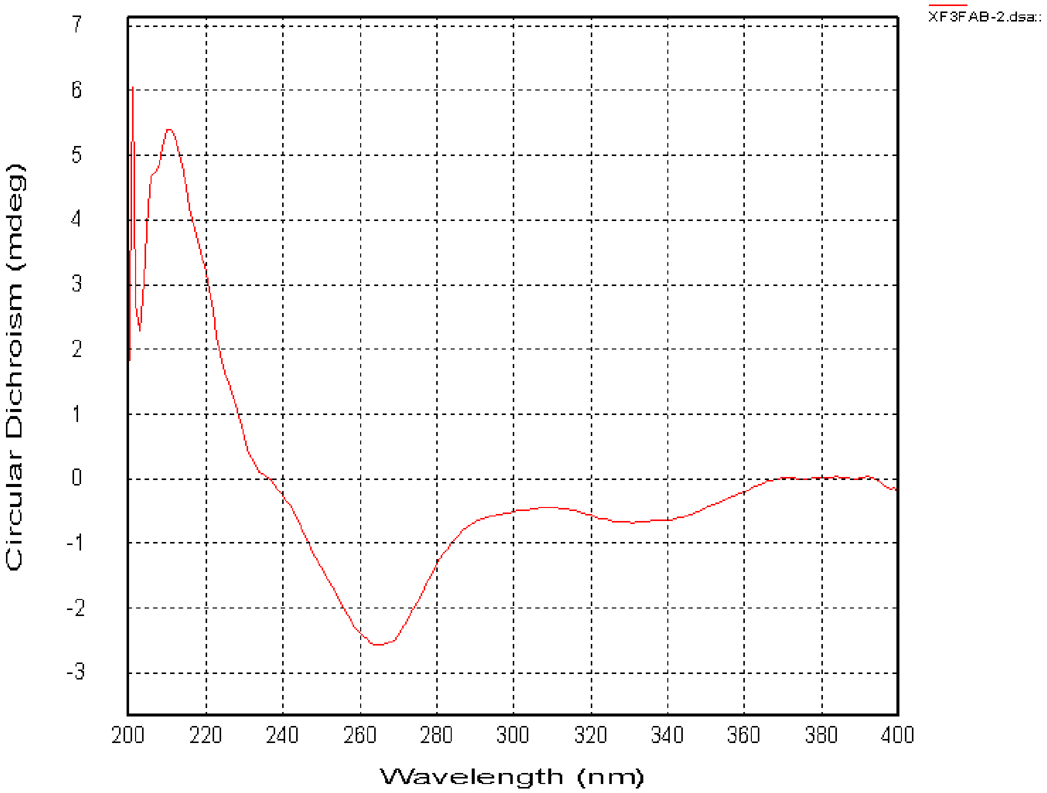

Figure S87. CD spectrum of **13**.

Reference for Supporting Information

1. Shinichi S., Taisuke I. *et al*. CJ-12,371 and CJ-12,372, Two novel DNA gyrase inhibitors fermentation, isolation, structural elucidation and biological activities. *J Antibiot* **48**, 134-142 (1995).

2. Li, C. Y. *et al*. Juglanones A and B: Two Novel Tetralone Dimers from Walnut Pericarp (*Juglans regia*). *Helv Chim Acat* **96**, 1031-1035 (2013).

3. Tetsuo K., Nigel C. V., Paul D. B., Monique S. J. S. Dihydroisocoumarins and a tetralone from *Cytospora eucalypticola*. *Phytochemistry* **62**, 779-782 (2003).

4. Zhu Y. H. *et al.* Screening and isolation of antinematodal metabolites against *Bursaphelenchus xylophilus* produced by fungi. *Ann. Microbiol.* **58**, 375-380 (2008).

5. Sanglard, D. *et al.* Susceptibilities of *Candida albicans* multidrug transporter mutants to various antifungal agents and other metabolic inhibitors. *Antimicrob. Agents Chemother.* **40**, 2300-2305 (1996).

6. Sanglard, D. *et al.* Cloning of *Candida albicans* genes conferring resistance to azole antifungal agents: characterization of *CDR2*, a new multidrug ABC transporter gene. *Microbiology* **143**, 405-416 (1997).

7. Mukherjee, P. K. *et al.* Mechanism of fluconazole resistance in *Candida albicans* biofilms: phase-specific Role of efflux pumps and membrane sterols. *Infection and immunity* **71**, 4333-4340 (2003).

8. Otzen, T. *et al.* Folate-synthesizing enzyme system as target for development of inhibitors and inhibitor combinations against *Candida albicans* synthesis and biological activity of new 2,4-diaminopyrimidines and 4‘-substituted 4-aminodiphenyl sulfones. *J. Med. Chem.* **47**, 240–253 (2004).
